# A systematic review of the nature and efficacy of Rational Emotive Behaviour Therapy interventions

**DOI:** 10.1371/journal.pone.0306835

**Published:** 2024-07-09

**Authors:** Ailish M. King, Carolyn R. Plateau, Martin J. Turner, Paul Young, Jamie B. Barker

**Affiliations:** 1 School of Sport, Exercise and Health Sciences, Loughborough University, Loughborough, Leicestershire, United Kingdom; 2 Department of Psychology, Institute of Sport, Manchester Metropolitan University, Manchester, United Kingdom; Universiti Pertahanan Nasional Malaysia, MALAYSIA

## Abstract

In the absence of a single comprehensive systematic review of Rational Emotive Behaviour Therapy interventions across all settings, we reviewed the methodological quality, effectiveness and efficacy of Rational Emotive Behaviour Therapy interventions on irrational/rational beliefs. We explored the impact of Rational Emotive Behaviour Therapy on wider outcomes (e.g., mental health) and identified the characteristics of successful interventions. PsycARTICLES, PsycINFO, Scopus, SPORTDiscus, and PubMed were systematically searched up to December 2023 with 162 Rational Emotive Behaviour Therapy intervention studies identified which included a validated measure of irrational/rational beliefs. Where possible, effect size for irrational/rational belief change was reported and data was analysed through a qualitative approach. Using the Mixed Methods Appraisal tool, methodological quality within the Sport and Exercise domain was assessed as good, whilst all other domains were considered low in quality, with insufficient detail provided on intervention characteristics and delivery. Most studies were conducted in the United States, within the Education domain, and assessed irrational beliefs in non-clinical adult samples. Overall, studies reported significant reductions in irrational beliefs, increases in rational beliefs and improvements in mental health outcomes (e.g., depression). More successful interventions were delivered by trained Rational Emotive Behaviour Therapy practitioners, adopted the ABC framework and were longer in duration. We highlight the importance of designing and conducting rigorous future Rational Emotive Behaviour Therapy research to generate clearer insights as to its impact on irrational/rational beliefs and mental health outcomes.

## Introduction

Rational Emotive Behaviour Therapy (REBT) was developed by Albert Ellis in the 1950s [e.g., 1], as arguably the first cognitive behavioural therapy (CBT; [2]). REBT was initially devised as a psychotherapeutic approach for use within clinical settings. It has since been applied across numerous domains, such as education [e.g., 3], counselling [e.g., 4], health [e.g., 5], occupational [e.g., 6], sport [e.g., 7] and exercise [e.g., 8]. Given the expansion of empirical research detailing the potential effectiveness and efficacy of REBT interventions globally and across domains, there is a need to provide a comprehensive and systematic synthesis of REBT interventions. In doing so, it is hoped to drive innovation and rigour in methods and REBT intervention development across research and applied practice globally and across domains.

A key premise of REBT is that one’s beliefs are at the heart of their emotional and behavioural reactivity. Beliefs are tacit and evaluative notions or ideas regarded as true, which are triggered in response to an event [9]. In REBT, there are two superordinate categories of belief, namely irrational and rational beliefs. Irrational beliefs are rigid, illogical and incongruent with reality. They underpin unhealthy negative emotions, dysfunctional cognitions, maladaptive behaviours and sabotage goal achievement [9–11]. In contrast, rational beliefs are flexible, logical and congruent with reality. They result in healthy negative emotions, functional cognitions, adaptive behaviours and facilitate goal achievement [9, 11]. Irrational and rational beliefs are the proposed primary mechanisms of change within REBT interventions [12]. In REBT, irrational beliefs are identified, disputed and weakened, whilst rational beliefs are developed and strengthened [13, 14] for the ultimate goal of emotional, cognitive and behavioural health and functionality thereby facilitating goal achievement (see [9, 15] for further information).

To date, the effectiveness (i.e., studies conducted in real-world naturalistic settings [16]) and efficacy (i.e., studies conducted in ideal and controlled circumstances, such as randomised control trials [16]) of REBT interventions have been summarised by five meta-analyses, all of which reported REBT interventions to be an effective form of psychotherapy for non-clinical, sub-clinical and clinical populations across a range of outcomes including irrational and/or rational beliefs, performance and mental health (see [12, 17–20]). The most recent review and meta-analysis conducted by David et al. [12], included eighty-two empirical studies spanning a 50-year period and a specific mechanism of change inquiry. Overall, the meta-analysis reported medium, significant effect sizes for REBT interventions on a range of outcomes including behavioural, cognitive, emotional, health, psychophysiological, quality of life, school performance and social skills at post-intervention and follow-up. While David et al.’s [12] was more comprehensive than previous reviews, there are two notable shortcomings. First, the mechanism of change inquiry on irrational and/or rational beliefs was limited given the large number of studies included that did not adopt a measure of irrational and/or rational beliefs. Consequently, this limits the ability to conclude that changes to outcome variables are a direct result of REBT interventions and specifically, via a change to irrational and rational beliefs. Second, there has been significant growth in REBT intervention studies in specific parts of the world (i.e., Africa) and within specific domains (i.e., sport and exercise) since David et al.’s [12] review. Indeed, David et al.’s [12] systematic review [12] largely omitted the sport and exercise domain (only one study was included), despite significant growth in REBT’s application within this domain since 2011 [15].

Accordingly, there is a pressing need to systematically synthesise and examine the growing body of empirical REBT research, specifically with regards to (1) the inclusion of studies which adopt a validated measure of irrational and/or rational beliefs, (2) to be inclusive of all domains within which REBT interventions are being conducted and (3) be reflective of the recent increase in REBT interventions across the globe (see [21], special issue). Further, it is prudent to comprehensively capture the current and exciting state of the research field as it grows in popularity across different therapeutic/non-therapeutic contexts. For clarity, the aim of our systematic review is to review the effectiveness and efficacy of REBT interventions on irrational and rational beliefs as well as additional outcomes, (e.g., wellbeing), through (a) synthesising and critiquing existing REBT interventions, (b) identifying and reviewing the characteristics of successful REBT interventions, and (c) comparing the methodological quality of REBT intervention research across domains. In doing so, our landmark review will generate a comprehensive knowledge base to facilitate the accurate transmission of scientific knowledge and guide researchers and practitioners in enhancing the design, delivery and reporting of future REBT interventions globally and across domains.

## Method

The systematic review followed Preferred Reporting Items for Systematic Reviews and Meta-analyses (PRISMA) guidelines [22, 23] and the PICO framework. The systematic review protocol was registered on Open Science Framework (https://doi.org/10.17605/OSF.IO/3CTGP). Ethical approval was not required given the nature of the study (i.e., systematic review).

### Search strategy

To ensure a comprehensive systematic literature search, four search strategies were employed. First, a preliminary literature search was conducted by the first author in August 2020 using the following electronic databases (and platform): PsycARTICLES (EBSCOhost), PsycINFO (EBSCOhost), Scopus (Elsevier), SPORTDiscus (EBSCOhost) and PubMed (National Library of Medicine; See S1 File for search strategy).The search strategy consisted of three concepts: (1) Intervention type (e.g., terms: REBT, RET, and Rational Coaching); (2) mechanism of change (e.g., terms: Irrational belief and dysfunction thought); and (3) outcome of interest (e.g., terms: Mental wellbeing and performance) using Boolean operators (i.e., AND and OR). The specific search terms used varied across each database to reflect their unique MeSH (Medical Subject Headings) and Index Terms. Truncation and wildcards were used with stem words to ensure variant words and spelling were identified. Searches were re-run at regular intervals during the review process; the most recent search was conducted in December 2023. Second, existing REBT theoretical reviews and meta-analyses were searched to identify further studies. Third, cited and citing reference searches (backward and forward citation searching) were conducted by the first author on the included articles. Finally, experts in the field were contacted to retrieve any additional published works that may have been missed.

### Inclusion and exclusion criteria

Studies were required to have delivered an REBT intervention based on Ellis’ theory of REBT [1]. They were required to be written in English; authors of non-English citations were contacted to request access to English versions. Only studies published in peer-reviewed scholarly journals were included. Studies were excluded if they did not include a validated outcome measure of either irrational and/or rational beliefs and if they did not measure change from at least pre-to post-intervention. To reiterate, it is of utmost importance to measure irrational and/or rational beliefs to be able to conclude REBT made an impact on the intended mechanisms, therefore contributing to the validation of REBT theory.

### Selection process

**Fig 1:**
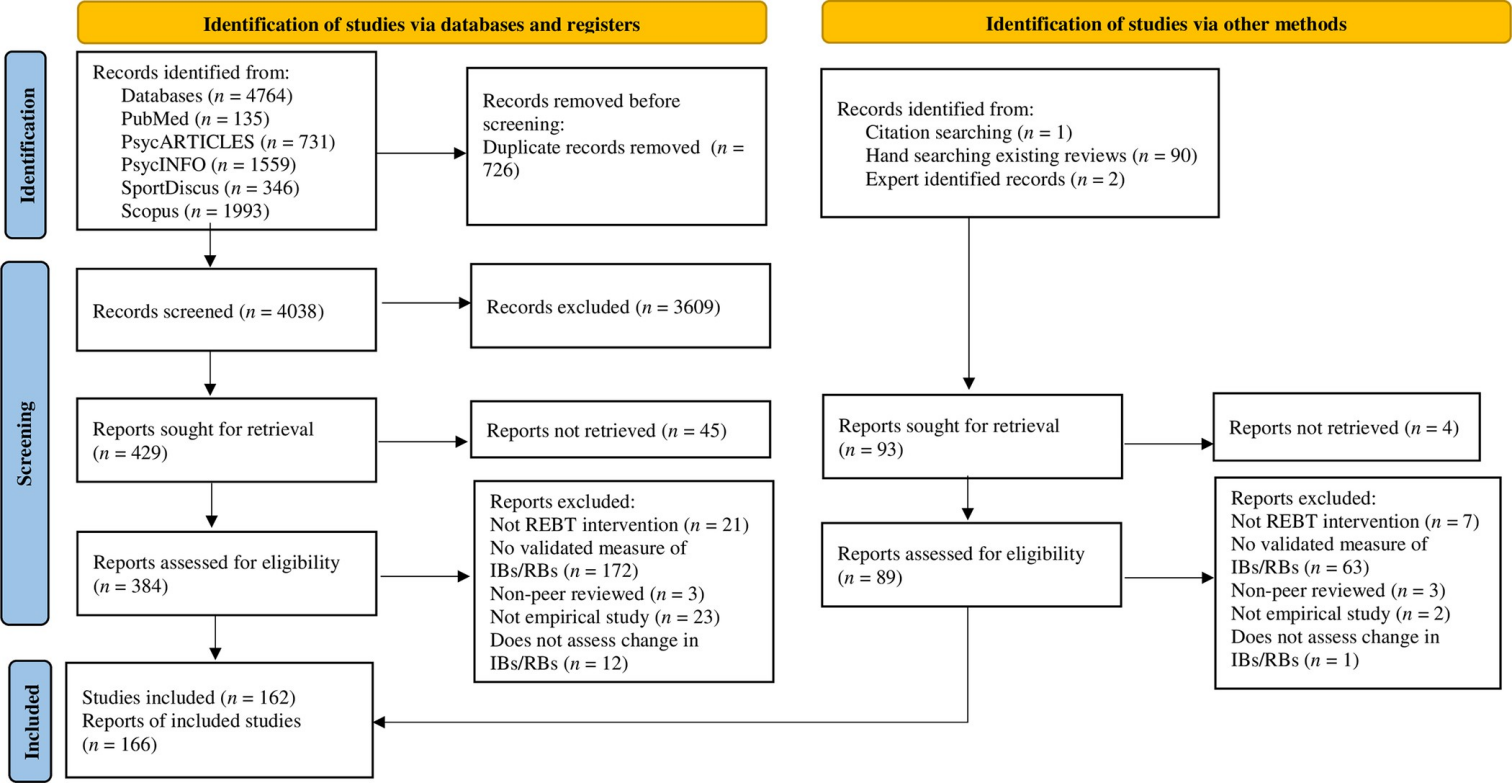
A PRISMA flowchart to represent the selection process. Note: Adapted to fit the new version of the flow diagram as the initial search began prior to publication of the new guidelines.

The article selection process is presented in Fig 1. Following searches, all records (*n* = 4,857) were imported to Endnote. Duplicates were identified and reviewed before removal (*n* = 726). Title and abstract screening was completed manually by the first author on all records (*n* = 4,131) and any uncertainties regarding inclusion were discussed by the research team and resolved by consensus. Forty-nine records were not retrievable, 39 of which were not available in English (21 articles were pre-2000) and 10 were not available through the inter-library loan service or other methods (i.e., where possible, authors were contacted to retrieve the article; 7 articles were pre-2000). All remaining retrievable records (*n* = 473) underwent full-text screening by the first author. The fourth author reviewed a random sample (10%) of the papers at the full-text screening stage for consistency. At the full-text screening stage, inter-rater agreement was 99%. Discrepancies were resolved through discussion until a consensus was reached.

### Data extraction and analysis

A data extraction form was developed and piloted based on the Template for Intervention Description and Replication (TIDieR [24]) and the REBT Competency Scale for Clinical and Research Applications [25]. The following information was extracted from each study: (a) Identification data (author[s], publication year, country); (b) sample (N, gender, age, type of sample including clinical status and retention); (c) study design; (d) outcome measures (mechanism of change [irrational beliefs and/or rational beliefs], additional outcome measures and assessment points); (e) intervention characteristics (type, frequency, duration, mode of delivery, intervention delivery personnel, setting, components of the intervention, materials, tailoring); (f) descriptive information related to treatment integrity (procedural reliability, adherence and fidelity); and (g) main study outcomes, which included process evaluation. Where possible, effect sizes were calculated and interpreted using Cohen’s *d* [26] which are reported in S2 File. In incidences of limited information, authors were contacted for additional information. If this method did not prove fruitful (generally, it was unsuccessful), the information was identified as ‘not reported’. The methodological quality of studies was assessed using the Mixed Methods Appraisal Tool Version 18 (MMAT [27]). This tool has been used successfully in systematic reviews evaluating interventions [e.g., 28], and enables the critical appraisal of qualitative, quantitative and mixed methods studies. Studies were rated with a ‘yes’, ‘no’ or ‘can’t tell’ for each criterion resulting in an overall quality score.

The fourth author reviewed the same random sample (10%) of papers at the full-text screening stage to check for correctness and consistency in the data extraction and MMAT process. Inter-coder reliability was calculated by dividing the number of agreed items from data extraction and methodological quality appraisal by the total number of items reviewed by both authors. Inter-rater agreement was 89% for data extraction and methodological appraisal. Discrepancies were resolved through discussion until a consensus was reached. Data was analysed through a qualitative synthesis adopting descriptive methods. A quantitative synthesis (e.g., meta-analysis) was not possible due to wide diversity of included studies and lack of homogenous samples [29].

## Results

Please refer to S2 File for a summary of study characteristics and S3 File for methodological appraisal grouped by domain.

### Study characteristics

Please see Fig 1 for the article selection process. A total of 166 reports, reporting on 162 studies met the inclusion criteria and were included in the review (see S4 File for a reference list of included studies). Reports on the same study were combined for data extraction (i.e., (a) [30, 31], (b) [32, 33], (c) [34, 35] (d) [36, 37] and (e) [38, 39]. One report detailed two separate intervention studies and therefore, was split into ‘Study 1’ and ‘Study 2’ for data extraction [40].

Included studies were published between 1972 and 2023 and interventions were conducted in 22 countries across 6 continents. Studies were mainly from the United States (*n* = 48), Romania (*n* = 24) and the United Kingdom (*n* = 24). The studies were classified into seven domains: Education (*n* = 59; e.g., school and university contexts with students), hospital and community healthcare (*n* = 25; e.g., hospital and residential care facility contexts with patients), organisational (*n* = 24 e.g. business contexts with employees), sport and exercise (*n* = 24; e.g., sport/exercise contexts with athletes/exercisers), self-identified healthcare need (*n* = 17; e.g., health care contexts with those who self-reported health concerns), relationships (*n* = 12; e.g., family and relationship support contexts with families or those in romantic relationships) and forensic (*n* = 1; e.g., custodial settings with those who were incarcerated).

The majority of studies included assessments pre- and post-intervention (*n* = 75), with the remaining studies also including a follow-up assessment which ranged from 2 weeks to 4 years post-intervention (*n* = 52). A few studies included two follow-up assessments, with the second follow-up ranging from 4 months to 1.5 years (*n* = 9) after intervention completion. Mid-intervention assessment points were adopted by 11 studies. Some studies assessed participants throughout the intervention, hence had multiple assessment points (*n* = 15), of which seven included a follow-up assessment.

### Methodological quality

Methodological quality was assessed by study design in line with MMAT criteria [27]. Quality scores varied across domains. For example, the average quality score (range: 0–100%) across domain was 44% for education, 40% for forensic, 49% for hospital and community healthcare, 49% for organisational, 48% for relationships, 45% for self-identified healthcare need and 71% for sport and exercise. This suggests higher quality studies were conducted within the sport and exercise domain; with studies typically adopting appropriate randomisation methods and validated measures as opposed to other domains where these aspects were less apparent S3 File.

The 162 included studies were categorised as follows: Randomised control trials (*n* = 82), non-randomised control trials (*n* = 77) and mixed method studies (*n* = 3). The average quality score was 47% for randomised control trials, 51% for non-randomised control trials and 93% for mixed method studies. Quality scores are summarised by study design below. Please refer to S3 File for the MMAT scores for individual studies.

#### Randomised control trials (n = 82)

Quality scores ranged from 0% to 100%, with studies scoring 0% *(n* = 5), 20% *(n* = 13), 40% *(n* = 27), 60% *(n* = 23), 80% *(n* = 12) and 100% *(n* = 2). The two criteria most commonly met from the MMAT were (1) complete outcome data was provided with an acceptable value of 80% (72% of studies met this criterion; *n* = 59) and (2) participants were comparable across groups at baseline (68% of studies met this criterion; *n* = 56) thereby suggesting good quality for those criteria. The quality criteria most commonly *not* met were (1) blinding of outcome assessors to the intervention condition *(*60% of studies did not meet this criterion; *n* = 49), (2) appropriate randomisation (63% of studies did not met this criterion; *n* = 52), and (3) if participants adhered to the assigned intervention (80% of studies did not meet this criterion; *n =* 66) thereby suggesting lower quality for those elements. In summary, 45% of randomised control trials scored 60% and above for quality.

#### Non-randomised control trials (n = 77)

Quality scores ranged from 0% to 100%, with studies scoring 0% *(n* = 4), 20% *(n* = 11), 40% *(n* = 21), 60% *(n* = 24), 80% *(n* = 13) and 100% *(n* = 4). The two criteria most commonly met from the MMAT were (1) participants represented the target population (75% of studies met this criterion; *n* = 58), and (2) measurements were appropriate regarding both the outcome and intervention (81% of studies met this criterion; *n* = 62) thereby suggesting good quality for those criteria. The criteria not met were (1) complete outcome data with an acceptable value of 80% (45% of studies did not meet this criterion; *n* = 35), (2) confounders accounted for in design and analysis (71% of studies did not meet this criterion; *n* = 55) and (3) if the intervention was administered as intended (83% of studies did not meet this criterion; *n* = 64) thereby suggesting lower quality for those elements. In summary, 53% of non-randomised control trials scored 60% and above for quality.

#### Mixed methods study (n = 3)

Quality scores ranged from 80% to 100%, with studies scoring 80% *(n* = 1) and 100% *(n* = 2). The mixed method study, quantitative and qualitive categories suggested good quality as each criterion was met by all or most of the included studies S3 File. In summary, 100% of mixed methods studies scored 80% and above for quality.

### Measures of the mechanism of change

A total of 61 distinct measures were identified, of which 58 were self-report questionnaires and three involved content analysis of participant’s thoughts to imagined/role play scenarios which were rated by assessors for irrational tendencies. Of the 61 measures, 51 assessed irrationality-only, two rationality-only and eight measured both irrationality and rationality. The measures used were broad in nature (*n* = 33) or specific to certain rational/irrational beliefs (e.g., Unconditional Self-Acceptance Questionnaire [41]; *n* = 3) participant age (e.g., Child and Adolescent Survey of Irrational Belief Scale [42]; *n* = 5), participant role (e.g., Parent Rational and Irrational Beliefs Scale [43]; *n* = 7) or domain (e.g., irrational Performance Beliefs Inventory [44]; *n* = 6). Some studies modified validated measures by only using specific subscales (*n* = 5) or reducing the number of items included (*n* = 6). Most studies adopted one measure to assess the alleged mechanism of change (*n* = 150) although some studies used two measures (*n* = 13). The most used scales were The Irrational Beliefs Test ([45]; *n* = 29), General Attitudes and Beliefs Scale–Short Form ([46] 1999; *n* = 18) and the irrational Performance Beliefs Inventory (44; *n* = 14). In summary, most measures adopted were self-report and broadly assessed irrational beliefs.

#### Participants

A review of participant characteristics was conducted; however, it is noted that 115 studies (71%) did not report complete participant information; thus, the following details are based upon information reported in the articles or provided by the author when contacted. Missing participant characteristics are noted in S2 File. Of the 162 studies, a total of 10,147 participants were recruited. A total of 5,507 participants received an REBT/REBT-related intervention and of the studies which employed a non-REBT related control/comparison group (*n* = 110), 3,482 were control participants (pre-intervention assignment numbers were not reported by 16% of studies; *n* = 26). The percentage of participants retained ranged from 54% to 100% across all studies with a mean of 96% (retention was not reported by 33% of studies; *n* = 53). The age of the participants ranged from nine to 74 years with a mean age of 30 years (mean age was not reported by 31% of studies; *n* = 50). Regarding gender, 116 studies sampled both male and female participants, 19 studies sampled male participants only and 13 studies sampled female participants only (gender was not reported by 9% of studies; *n* = 14). Of the 162 studies, recruited participants were deemed to be clinical (*n* = 26), sub-clinical (*n* = 17) or non-clinical (*n* = 119). In summary, participants were typically non-clinical adult males and females.

#### The nature of REBT interventions

One hundred and eighteen studies (73%) did not report at least one aspect related to the nature of the interventions. The following information is based upon information reported in the articles or provided by the author. Most interventions were based on REBT and associated principles (*n* = 137); some studies combined REBT interventions with other interventions hence were multimodal in nature (*n* = 25; e.g., REBT combined with personal disclosure mutual sharing; [47]). Of note, some multimodal studies included an REBT-only comparison group or designs (i.e., single-case ABC between-groups design) which enabled the effects of REBT and the multimodal intervention to be determined separately [e.g., 47]. Of the 162 studies, 46 were classed as counselling/ psychotherapy (i.e., if the intervention was designed to treat diagnosed mental health conditions and delivered by trained clinicians), 104 were classed as psychoeducation (i.e., if the intervention was largely informational in nature), eight as coaching (i.e., if the intervention was labelled as this, the intervention was delivered to non-clinical populations and the primary focus was goal achievement; [48]) and four as brief self-statement interventions (i.e., if the intervention involved participants reading cards which displayed irrational/rational beliefs with no practitioner interaction). Most interventions adopted a face-to-face format (*n* = 145), whilst a small number used telephone/video calls (*n* = 4), a mixture of face-to-face and telephone/video calls (*n* = 6) or computer-based delivery (*n* = 7). Further, the interventions were typically delivered in a group setting (*n* = 99), or one-to-one (*n* = 38) with some interventions combining both formats (*n* = 4). Twenty-one studies did not report this information. The interventions were mainly delivered by one practitioner (*n* = 73), some were co-delivered (*n* = 17) and one intervention assigned a named practitioner to each participant who they could contact while completing the intervention [49]. Some studies did not use a practitioner due to the nature of the intervention (*n* = 10; e.g., self-statements or computer-based delivery). There was no clear trend for frequency and duration of the interventions. The number of sessions delivered ranged from one to 70, with durations from one day to nine months with a range of 15 minutes to 140 hours delivery time. In summary, most studies were psychoeducational in nature and were delivered face-to-face in group settings by one practitioner. Frequency and duration were highly variable.

With regards to the specific REBT components of the interventions, most studies referred to using at least the ABC framework (*n* = 103; [1]) of these, only eight studies explicitly referred to the G (goal) aspect of the GABCDE framework [14]. Three studies referred to an ‘F’ in the framework representing ‘functional emotions’ which is not part of the original framework [e.g., 50]. Fifty-five studies did not report any detail on the framework used and four studies did not include the framework. Most studies included disputation of irrational beliefs (*n* = 136). The vast majority of studies reported focusing on developing and/or strengthening rational beliefs (*n* = 118). Most studies reported setting homework (*n* = 102) which varied in task and frequency (e.g., ranging from one breathing practice to daily reading, completion of forms and skill practices). Seven studies did not set homework (e.g., single session interventions); Fifty-three studies did not include details with regards to homework tasks. In summary, most studies adopted the ABC framework, disputed irrational beliefs, strengthened rational beliefs and set homework.

#### Primary outcomes

Changes in irrational and rational beliefs from pre-to-post intervention (and follow-up where appropriate) were assessed. Results are grouped by domain and study design. Of note, studies with no statistically significant findings also includes studies which did not report on belief change and/or conduct formal statistical analysis.

#### Education (n = 59)

Participants from the education domain were from schools, colleges and universities. In most studies (*n* = 28), students were non-clinical adults who were attending college /university with the intervention delivered within the educational setting (e.g., classroom).

*Randomised controlled trials (n = 35)*. Twenty-three studies reported statistically significant reductions in irrational beliefs from pre- to post- for intervention participants in comparison to controls (*n* = 20 studies employed a control group). Small (*n* = 1 [51]), medium (*n* = 3) and large (*n* = 9) effect sizes were reported. Of the ten studies that included a follow-up assessment (ranged from 6 weeks to 8 months), eight reported reductions in irrational beliefs to be maintained. Three studies reported statistically significant increases in rational beliefs from pre- to post- for intervention participants [52] in comparison to controls (*n* = 2 [53, 54]). Only one study included a follow-up assessment and reported no between group differences in rational beliefs at follow-up [54]. Two studies did not report any statistically significant reductions in irrational beliefs, or increases in rational beliefs, from pre- to post-intervention in comparison to controls [55, 56]. Four studies adopted a multimodal REBT intervention whereby REBT was combined with other interventions (e.g., hypnotherapy [57]). All studies reported statistically significant reductions in irrational beliefs from pre- to post- for REBT multimodal intervention participants in comparison to controls (large effect size [57]) with sustained change for two studies.

*Non-randomised control trials (n = 24)*. Seventeen studies reported statistically significant reductions in irrational beliefs from pre- to post- for intervention participants in comparison to controls (*n* = 8 studies included a control group). Medium (eight studies) and large (four studies) effect sizes were reported. Two studies included a follow-up assessment (ranging from 1 to 4 years [38, 39, 58]); effects were maintained in one study [38, 39]. One study reported statistically significant increases in rational beliefs from pre- to post- for intervention participants in comparison to controls with a medium effect size [59]. Six studies did not report any statistically significant reductions in irrational beliefs from pre- to post-intervention in comparison to controls.

Overall, studies from the education domain reported statistically significant reductions in irrational beliefs from pre- to post-intervention which typically were of medium and large effect size and statistically significant increases in rational beliefs from pre- to post-intervention. The maintenance of irrational and rational belief change beyond the intervention was mixed. Interventions successful at reducing irrational beliefs and/or increasing rational beliefs were of a longer duration (i.e., > 4 weeks) and delivered by trained REBT practitioners (e.g., licensed psychologists). Moreover, they involved strengthening rational beliefs, homework following each session (e.g., reflective tasks to practice the skills learned) and the ABC framework.

#### Forensic (n = 1; randomised controlled trial)

The single RCT in the forensic domain included eighty-three non-clinical adults who were deemed extremists/terrorists. The study identified statistically significant reductions in irrational beliefs in the REBT intervention participants compared with controls from pre- to post-intervention (large effect size [60]). Rational beliefs were not assessed within this domain. The intervention was characterised by moderate duration (i.e., > 12-weeks) and included homework although no further details were provided.

#### Hospital and community healthcare need (n = 25)

Participants from the hospital and community healthcare need domain were in-/out- patients who had a physical/mental health diagnosis and were typically referred to the intervention by medically trained personnel. In general (*n* = 18 studies), the participants were clinical adults, and interventions were delivered within a hospital/medical centre.

*Randomised controlled trials (n = 14)*. Six studies reported statistically significant reductions in irrational beliefs from pre- to post- for intervention participants in comparison to controls. Medium [61, 62] and large effect sizes [63] were reported. Two studies included a follow-up assessment (1 month) and reported that effects were maintained ([63, 64] with large effect size [63]). Four studies did not report any statistically significant decreases in irrational beliefs from pre- to post-intervention. Three studies adopted a multimodal REBT intervention and noted statistically significant reductions in irrational beliefs from pre- to post-intervention [e.g., 65, 66] and in comparison to controls (large effect size) with sustained effects for one study [65].

*Non-randomised control trials (n = 11)*. Seven studies reported statistically significant reductions in irrational beliefs from pre- to post- for intervention participants (*n* = 4 studies included a control group [67–69]. Small [70] and large effect sizes were reported [69, 71]. Three studies did not report any statistically significant decreases in irrational beliefs from pre- to post- which included one multimodal intervention [72].

Overall, studies in this domain reported mixed findings. When statistically significant changes in irrational belief existed, these were mostly of medium to large effects, and many were maintained at follow-up. Rational beliefs were not assessed within this domain. Interventions successful at reducing irrational beliefs within this domain included a range of delivery materials (e.g., manual and audio tapes), strengthening of rational beliefs, daily homework tasks and the ABC framework.

#### Organisational (n = 24)

Participants were employees from a range of sectors including bankers, construction workers and emergency service personnel. The participants were non-clinical adults, and interventions were typically delivered in the organisation’s building (e.g., room within police headquarters).

*Randomised controlled trials (n = 10)*. Seven studies reported statistically significant reductions in irrational beliefs from pre- to post- for intervention participants in comparison to controls. Five studies reported effect sizes of which large effect sizes. Eight studies included a follow-up assessment (ranging from 10 weeks to 1 year) and reported significant findings were maintained across all studies with small [73, 74], medium [75, 76] and large [77] effect sizes. One study did not conduct formal statistical analysis and/or report on irrational belief change [78]. Two studies involved multimodal REBT interventions and statistically significant pre- to post- reductions in irrational beliefs were reported [36, 37, 79] Of note, only one study adopted an REBT-only comparison intervention with no statistically significant difference observed between the multimodal and comparison group [36, 37].

*Non-randomised control trials (n = 13)*. Twelve studies reported statistically significant reductions in irrational beliefs from pre- to post- for intervention participants in comparison to controls (five studies included a control group). Small [80], medium (five studies [e.g., 6] and large (five studies [e.g., 81] effect sizes were reported. Four studies included a follow-up assessment (ranging from 3 to 18 months); only one study reported maintenance of effects [82]. Four studies reported statistically significant increases in rational beliefs from pre- to post- for intervention participants with medium [81] and large [6, 83] effect sizes reported.

*Mixed methods study (n = 1)*. One study delivered an REBC intervention to fifty senior police personnel [84]. Statistically significant reductions in irrational beliefs were observed in the intervention group compared with controls from pre- to post-intervention (large effect size) which was maintained at six-month follow-up (medium effect size).

In general, studies reported statistically significant reductions in irrational beliefs and increases in rational beliefs from pre- to post-intervention which typically were of medium and large effect size. Typically, effects were maintained at follow-up. Successful interventions were characterised by face-to-face delivery by trained practitioners which involved one-to-one formats. Daily homework was set and reviewed and the ABCDE framework was used in conjunction with elements to enhance procedural reliability (e.g., manual).

#### Relationships (n = 12)

Participants were typically non-clinical and were either involved in a romantic relationship or were a parent/guardian–child dyad.

*Randomised controlled trials (n = 7)*. Three studies reported statistically significant reductions in irrational beliefs from pre- to post- for intervention participants (only one study included a control group [85]. Large effect sizes [86] were reported. Only one study included a follow-up measure (10-months) and reported maintained effects [86]. Two studies did not report significant changes in irrational beliefs [87, 88]. Two studies involved multimodal REBT interventions and noted statistically significant reductions in irrational beliefs from pre- to post-intervention (small effect sizes–[89]; large effect sizes–[90]). Notably, only one study included an REBT-only comparison group with no statistically significantly difference observed between groups [89].

*Non-randomised control trials (n = 5)*. Three studies reported statistically significant reductions in irrational beliefs from pre- to post- for intervention participants with medium [91] and large effect sizes [92] and in comparison to controls when adopted [91, 93]. One study reported statistically significant increases in rational beliefs from pre- to post- for intervention participants in comparison to a control [93]. Two studies did not report any statistically significant reductions in irrational beliefs from pre- to post-intervention [94, 95].

Overall, studies reported statistically significant reductions in irrational beliefs and increases in rational beliefs from pre- to post-intervention which typically were of medium and large effect sizes. Generally, effects were maintained at follow-up. Interventions successful at reducing irrational beliefs and/or increasing rational beliefs were characterised by high attendance, a longer duration (i.e., > 6 weeks), longer sessions (i.e., ≥ 1.5 hours), employed the ABC framework and included daily homework.

#### Self-identified healthcare need (n = 17)

Participants in this domain were those who self-identified as having a physical/mental health need (e.g., anxiety, phobias and headaches). Typically (*n* = 10 studies), adults did not have a pre-existing diagnosis and referred themselves to the intervention.

*Randomised controlled trials (n = 11)*. Six studies reported statistically significant reductions in irrational beliefs from pre- to post- for intervention participants in comparison to controls (five studies included a control group). Large effect sizes were reported (five studies). One study reported significant reductions in irrational beliefs for an intervention group, that was not statistically significant to an active control condition [96]. Three studies included a follow-up assessment (ranging from 2 weeks to 6 months [97–99]); two studies reported maintained effects [97, 98 –medium effect size].

Three studies involved multimodal REBT interventions and reported statistically significant reductions in irrational beliefs from pre- to post- for intervention participants in comparison to controls (large effect sizes); findings were maintained at follow-up for two studies [5, 100]. One multimodal study did not conduct formal statistical analysis and report on irrational belief change [101].

*Non-randomised control trials (n = 6)*. Four studies reported statistically significant reductions in irrational beliefs from pre- to post- for intervention participants in comparison to controls (two studies included a control group; [49, 102]). One study reported a large effect size [49]. Two studies involved multimodal REBT and reported statistically significant reductions in irrational beliefs for intervention participants [103] in comparison to an active and true control (large effect size [104]).

Overall, studies from the self-identified healthcare need domain reported statistically significant reductions in irrational beliefs from pre- to post-intervention which typically were of large effect size. Generally, effects were maintained at follow-up. Rational beliefs were not assessed within this domain. Interventions successful at reducing irrational beliefs employed the ABC framework, were delivered by REBT trained practitioners, included elements to enhance procedural reliability (e.g., manual) and involved homework.

#### Sport and exercise (n = 24)

Participants from the sport and exercise domain were athletes, athlete support personnel and exercisers from the general population.

*Randomised controlled trials (n = 4)*. Two studies reported statistically significant reductions in irrational beliefs from pre- to post- for intervention participants in comparison to controls (large effect size [105]) with sustained effects at follow-up (ranging from 1 month to 4 months [105, 106]). One study did not conduct formal statistical analysis [107]. One multimodal intervention reported statistically significant reductions in irrational beliefs and significant increases in rational beliefs from pre- to post-intervention for REBT-only participants. The greatest benefits were noted for multimodal intervention participants [108].

*Non-randomised control trials (n = 18)*. Six studies reported statistically significant reductions in irrational beliefs from pre- to post- for intervention participants with sustained effects at follow-up assessment (range of 3 months to 1 year) for four studies. Six studies reported effect sizes of which large effect sizes. Three studies measured rational belief change and one study reported statistically significant increases in rational beliefs from pre- to post- intervention (large effect size) which was maintained at follow-up [8]. Eleven studies did not conduct formal statistical analysis and report on irrational belief change. One multimodal intervention reported statistically significant reductions in irrational beliefs from pre- to post-intervention (large effect sizes [47]).

*Mixed methods studies (n = 2)*. Two studies [109, 110] reported significant reductions in irrational beliefs from pre- to post-intervention with large effect sizes. One study also reported significant increases in rational beliefs (large effect size) and noted that changes were maintained at follow-up [110].

In summary, studies from the sport and exercise domain reported statistically significant reductions in irrational beliefs from pre- to post-intervention which typically were of large effect size and were generally maintained at follow-up. Findings in relation to rational beliefs were mixed. Many studies did not conduct formal statistical analysis in favour of visual analysis and reporting effect sizes for single-case designs [e.g., 111]. Interventions successful at reducing irrational beliefs and/or increasing rational beliefs were delivered in-person by trained REBT practitioners which sometimes involved one-to-one formats, the GABCDE framework was employed and a variety of regular homework tasks were set (e.g., cognitive and behavioural).

### Secondary outcomes

A total of one-hundred and forty-four studies (89%) included at least one additional outcome measure. The secondary outcomes assessed by studies included in the review were grouped into eight categories: (1) Cognitive (*n* = 19), (2) individual differences (*n* = 42), (3) mental health/ill-health (*n* = 111), (4) parenting (*n* = 7), (5) performance (n = 21), (6) physical-related (*n* = 22) and (7) social skills (*n* = 19) and (8) miscellaneous (*n* = 14). The most common secondary outcomes included were mental health/ill-health (*n* = 111) and individual differences (*n* = 42). Secondary outcomes were mostly measured via self-report measures (*n* = 143).

### Cognitive

Nineteen studies measured the impact of REBT on cognitive-related outcomes, including thoughts, problem solving and decision making. Three studies were multimodal interventions. Of the sixteen REBT-only interventions, twelve reported statistically significant improvements from pre- to post- for REBT intervention participants in comparison to controls (five studies included a control group) with sustained effects for two studies [112, 113]. Four studies reported non-significant findings. Two multimodal interventions reported statistically significant improvements in cognitive outcomes from pre- to post-intervention in comparison to controls with sustained effects [100]. Overall, most studies reported significant improvements on cognitive outcomes from pre- to post-intervention.

### Individual differences

Forty-two studies measured the impact of REBT interventions on individual differences, such as distinguishing characteristics and traits, including self-efficacy, personality characteristics and locus of control; seven were multimodal interventions. Of the thirty-five REBT-only interventions, thirty-one reported significant improvements from pre- to post- for REBT intervention participants in comparison to controls (24 studies included a control group) with sustained effects for eight studies. Four studies did not find any significant effects [e.g., 82]. Seven studies involved multimodal interventions and findings were mixed; where significant improvements were noted, these were largely not maintained at follow-up. Overall, most studies reported significant improvements on individual difference outcomes from pre- to post-intervention.

### Mental health/ill-health

One hundred and eleven studies measured the impact of REBT on mental health/ill-health secondary outcomes, including outcomes such as anxiety and depression; eighteen of which were multimodal interventions. Of the ninety-three REBT-only interventions, sixty-six studies reported statistically significant improvements from pre- to post- for REBT intervention participants in comparison to controls (48 studies included a control group) with sustained effects for eighteen studies. Five studies reported statistically significant improvements from pre- to post-intervention for both REBT intervention participants and controls. Eighteen studies involved multimodal interventions and fourteen studies reported statistically significant improvements from pre- to post-intervention for multimodal intervention participants. Twenty-five studies reported non-significant findings, which included three multimodal intervention studies [36, 37, 104]. In summary, two thirds of studies reported significant improvements on mental health/ill-health from pre- to post-intervention.

### Parenting

Seven studies measured the impact of REBT on parenting-related outcomes, such as problems related to the child, parent competency and parent-child interactions; one of which was a multimodal intervention [89]. Of the six REBT-only interventions, five studies reported statistically significant improvements from pre- to post-for REBT intervention participants in comparison to controls when adopted (three studies) with sustained effects for one study [85]. The multimodal intervention reported significant improvements from pre- to post-for the multimodal REBT group and an REBT-only comparison group [89]. Overall, most studies reported statistically significant improvements from pre- to post-intervention.

### Performance

Twenty-one studies measured the impact of REBT on performance related outcomes, such as academic grade point average, achieving work-based targets and sport competition results; five of which were multimodal interventions. Of the sixteen REBT-only interventions, nine studies reported statistically significant improvements from pre- to post- for REBT intervention participants in comparison to controls (five studies included a control group) with sustained effects for five studies. Seven studies did not identify any significant findings. Five studies involved multimodal interventions whereby REBT was combined with alternate interventions, such as motivational interviewing [114]; two studies identified statistically significant improvements for the intervention groups. In summary, over half of studies reported statistically improvements in performance from pre- to post-intervention.

### Physical-related

Twenty-two studies measured the impact of REBT on physical-related secondary outcomes, such as cortisol, pain and vitality; eight of which were multimodal interventions. Of the fourteen REBT-only interventions, four studies reported statistically significant improvements from pre- to post- for REBT intervention participants in comparison to controls (three studies included a control group) with sustained effects for two studies [63, 115]. Ten studies did not find any significant outcomes. Eight studies involved multimodal interventions and three studies reported statistically significant improvements, while five studies did not find any improvements. In summary, most studies did not report statistically significant improvements from pre- to post-intervention.

### Social skills

Nineteen studies measured the impact of REBT on social-skills outcomes such as assertiveness and conflict resolution, five of which were multimodal interventions. Of the fourteen REBT-only interventions, eleven studies reported statistically significant improvements from pre- to post- for REBT intervention participants in comparison to controls (six studies included a control group [e.g., 112]. One study reported sustained effects at follow-up [116]. Three studies did not find any significant outcomes. Five studies involved multimodal interventions and four studies reported statistically significant improvements from pre- to post- for multimodal intervention participants in comparison to controls. In summary, most studies reported statistically improvements in social skills from pre- to post-intervention.

### Miscellaneous

A myriad of eclectic outcomes were assessed in fourteen studies. Within this category, outcomes included aspects such as mindfulness, intervention-related outcomes (e.g., REBT knowledge) and life satisfaction. One study was a multimodal intervention [72]. Of the thirteen REBT-only interventions, seven studies reported statistically significant improvements from pre- to post- for REBT intervention participants in comparison to controls (five studies included a control group).

### Participant experiences

Forty-five of the studies included social validation which involves seeking participant experiences of the intervention to determine satisfaction and to understand, evaluate and document the impact of the intervention [117]. A range of methods were used with the most popular format being questionnaire (*n* = 17) and semi-structured interviews (*n* = 9). Three studies triangulated the findings with other key stakeholders (e.g., the athlete’s coach [118]). Overall, the interventions were reported as enjoyable, and useful as it enhanced participants’ motivation, psychological wellbeing, self-awareness and management of emotions, cognitions and behaviours. Some participants reported enhanced performance although these findings were mixed. Regarding mode of delivery, group formats were found to be most supportive. In some studies, participants highlighted that they would have liked additional time to practice the skills, understand the framework more and share experiences with group members.

## Discussion

The aim of our systematic review was to comprehensively review the efficacy and effectiveness of REBT interventions on irrational and/or rational beliefs and additional outcomes, such as health and performance. In total, 162 studies fulfilled the inclusion criteria. Our review highlights that REBT interventions have been delivered across the globe, with clinical, sub-clinical and non-clinical participants across a wide variety of domains, including education and healthcare [e.g., 40, 64]. The interventions were typically psychoeducational in nature, adopted the ABC framework, included homework and were delivered face-to-face in group settings by a trained practitioner. Intervention duration and frequency of delivery were found to be highly variable. Overall, studies reported significant decreases in irrational beliefs [e.g., 97] and increases in rational beliefs [e.g., 8] from pre- to post-intervention which were generally maintained at follow-up. Most studies reported statistically significant improvements on secondary outcomes, from pre- to post-intervention. Successful interventions were characterised by a longer duration (i.e., > 4 weeks), delivered by trained REBT practitioners, adopted the ABC framework and involved daily homework. Methodological quality was deemed to be adequate across domains and highest in sport and exercise.

### Efficacy

Most studies reported statistically significant reductions in irrational beliefs [e.g., 97] and increases in rational beliefs [e.g., 8] from pre- to post-intervention. The findings were generally maintained at follow-up. Specifically, statistically significant reductions in irrational beliefs from pre- to post-intervention were reported by the educational, forensic, organisational, relationships, self-identified healthcare need and sport and exercise domains with medium to large effect sizes. The findings were maintained for the organisational, relationships, self-identified healthcare need and sport and exercise domains. Rational beliefs were measured to a lesser extent and only within the educational, organisational, relationships and sport and exercise domains. Specifically, statistically significant increases in rational beliefs from pre- to post-intervention were reported by the educational, organisational and relationships domains with medium to large effect sizes. Findings were mixed for the sport and exercise domain. The findings were maintained for the organisational and relationships domains. These results are in-line with previous systematic reviews/meta-analyses which also reported REBT to be effective in reducing irrational and increasing rational beliefs [e.g., 12].

Most studies reported statistically significant improvements in secondary outcomes from pre- to post-intervention (e.g., parenting [85]). Specifically, most studies reported statistically significant improvements from pre- to post-intervention for outcomes related to cognitive, individual differences, mental health/ill-health parenting and social skills. Over half of studies reported statistically significant improvements in outcomes related to performance and miscellaneous from pre- to post-intervention. Further, most studies did not report statistically significant improvements from pre- to post-intervention for physical-related outcomes. These results corroborate the findings of previous systematic reviews/meta-analyses which found REBT to be effective in improving a range of additional outcomes [e.g., 12].

It is worth highlighting that some studies were multimodal in nature whereby REBT was combined with another intervention, such as personal disclosure mutual sharing [e.g., 47]. Unfortunately, these findings are difficult to interpret given that many of these studies did not include an REBT-only comparison group or designs which did not enable examination of separate intervention components or those that were integral to the improvements observed. Psychotherapy integration is on the rise [119]. It is clear that REBT can be successfully integrated with other approaches which align to REBT’s key principles, such as mindfulness [120]. An important next step when using multimodal REBT interventions is to compare these to an REBT-only comparison group.

### Nature of REBT

The articles included in this systematic review are underpinned by REBT but have been labelled a variety of names due to an evolution from Rational Therapy to Rational Emotive Therapy to REBT [1, 121] as well a result of its domain, such as Rational Emotive Education (REE). More recently, the ‘therapy’ aspect of the intervention is increasingly being omitted, such as studies that delivered Rational Emotive Behaviour Coaching [e.g., 82]. It has been proposed that the renaming of interventions when they are not delivered as a form of therapy to patients within a clinical setting reflects the participant and their context more accurately [122]. Regarding labelling, it is also noted that several studies failed to identify the intervention as REBT and instead, labelled the intervention as ‘CBT’ or ‘Cognitive Restructuring’ despite the intervention’s grounding within REBT and use of key principles such as the ABC framework [e.g., 87]. Ellis alludes to this in earlier works citing that REBT is not always acknowledged or given due credit [13].

Nevertheless, the inconsistent and inaccurate labelling of REBT interventions presents multiple challenges identified throughout this review, such as confusion regarding the theoretical position and technique used in the intervention and issues when attempting to identify, collate and synthesise REBT intervention research [123]. We urge researchers to correctly label interventions as REBT and avoid using CBT given that CBT represents a family of therapies as opposed to being a monolithic approach it is often assumed to be [123]. Using a correct and consistent label serves to acknowledge and credit the underlying theory, assists with the comprehensive identification and synthesis of REBT related research and enhances accuracy of the field. We propose that the labelling of REBT interventions should only be altered in discussions with participants to enhance intervention acceptance [122]. In doing so, it is hoped to provide clarity regarding REBT intervention research.

Within the included studies, the core framework of REBT, namely the GABCDE framework was often omitted [124]. Many studies reported on using at least the ABC framework but only few studies included the ‘G’ (i.e., goals) of the GABCDE framework. It is paramount that goals are considered when delivering REBT interventions given their contribution to the A’s, B’s and C’s [15]. To illustrate, goals influence the extent to which an activating event is relevant and determine the consequences. Thus, practitioners should utilise the GABCDE framework in full given its interdependent, reciprocal nature and useful point of intervention (i.e., modification of goals) to bring about functional emotional, cognitive, physiological and behavioural responses [14, 47, 125].

The GABCDE framework can be reinforced by homework which contributes to the participant’s understanding and ability to use REBT autonomously [126, 127]. Most studies reported using homework although it was omitted or not reported in approximately one third of the included studies. Research has demonstrated that participants who embrace homework, derive the most benefit from REBT interventions [e.g., 128]. Therefore, we encourage practitioners and researchers to set innovative behavioural, emotional and cognitive tasks and implement methods to enhance homework adherence. One suggestion is to use smartphones given their popularity, portability and programmability. Namely, The Smarter Thinking App could be used which encourages the autonomous use of REBT [129, 130] and has been used by REBT practitioners working within performance settings [131]. The app enables in-the-moment digitised disputation of irrational beliefs and promotes the adoption of rational beliefs which therapists can review. The app meets several proposed guidelines for mobile phone apps to maximise CBT homework compliance such as congruency with theory, in the moment self-assessment and opportunity for therapist feedback [132]. Automating and digitising routine components of therapy saves practitioner time and enables mass participation therefore, could be an attractive option [133].

One issue encountered throughout the systematic review was the quality of research and intervention descriptions within the included articles. Over two thirds of studies (70%) did not report on at least one aspect related to participant information, such as age and gender with similar findings observed for the reporting of intervention aspects (72%), such as mode of delivery. Moreover, of the intervention details reported, some were poorly described and contained errors. This incomplete, inaccurate and inadequate practice leads to issues when trying to replicate studies, refine theories, identify common characteristics of effective interventions, apply knowledge in real-world settings, build upon extant research and evaluate methods, ultimately hindering scientific progression [24, 134]. A shared responsibility approach should be adopted whereby authors, reviewers, and journal editors are all responsible for accurate, transparent, and complete reporting [134]. To assist in the completeness of intervention description, reporting checklists and guidelines should be utilised [135], such as TIDieR [24] or Intervention Mapping [136]. Further, to bridge the communication gap between researchers and practitioners, it is important that the reporting of interventions also includes implementation guidelines given that these peer-reviewed articles are often the primary source consulted by practitioners [137]. While we appreciate, journal publishing restrictions (i.e., format and length), authors should endeavour to make additional intervention material available using online supplementary materials or websites [24]. The adoption of complete intervention description is necessary to enhance scientific rigour, efficiently move the field forward and enable empirical evidence-based practice [134, 138].

Overall, interventions were delivered face-to-face and typically adopted a group format. Social validation data revealed the supportive nature of the group and how it served to normalise responses. However, REBT has also been found to be more effective in one-to-one modes as it enables the intervention to be appropriately tailored and the development of a strong working alliance, the latter of which is often deemed central to the effectiveness of interventions [14, 15]. More recently, technology has been used to deliver REBT interventions, such as computer modules or online games [e.g., 49, 139]. Given that technology saves practitioner time and facilitates large scale delivery, the use of online methods to supplement an in-person intervention, may prove to be an accessible, cost-effective and environmentally friendly adjunct [133]. A combined approach is preferable whereby technology complements and contributes to the delivery of REBT alongside the practitioner as opposed to a replacement method to retain a strong working alliance between client and practitioner [14, 133]. We encourage practitioners and researchers to explore how in-person group and one-to-one formats could be successfully combined with technology-based elements to deliver effective interventions to populations on a wider scale while maintaining essential clinician/practitioner judgement, expertise and interpersonal skills.

### Measures

One inclusion criterion for our systematic review was the inclusion of a validated outcome measure of irrational and/or rational beliefs change. Notably, a large proportion of studies failed to meet this criterion (*n* = 248) and were thus excluded despite delivering REBT interventions. Failure to measure the underlying mechanistic processes limits our understanding and means we cannot validate REBT theory [140]. Therefore, we encourage the inclusion of irrational and/or rational beliefs measures when delivering REBT interventions.

The measures used by the studies included in the review vary. Overall, there is a lack of exclusive measurement of rational beliefs. This is interesting as rational beliefs are also considered a mechanism of change and are not orthogonal to irrational beliefs [9]. Included interventions were instead more commonly reporting on the disputation of irrational beliefs as opposed to strengthening and developing rational beliefs. However, given that rational beliefs result in healthy emotions, functional cognitions and adaptive behaviours [9], REBT could be applied as a preventative and positive approach to promote rationality and positive wellbeing [141]. By doing so, it is hoped that positive mental health outcomes will arise and REBT will not merely be viewed as a form of treating dysfunction.

The systematic review also highlighted issues regarding the lack of clarity, transparency, validity and at times, inaccuracy of measurement of irrational and/or rational beliefs adopted. Some studies amended validated measures of irrational/rational beliefs by removing various items and thus using a non-validated version of the measure. Moving forwards, we urge those within the field to accurately label and reference the measures used, avoid selecting some items from the measure unless necessary and clearly outline any modifications made. By doing so will contribute to research transparency, valid interpretations and meaningful comparisons. If researchers are developing new measures, it is prudent to publish the description and validation of the measure in a timely fashion to enable transparency, increase confidence and aid in clear referencing.

A significant shortcoming of the field is the absence of a psychometrically sound “gold standard” measure of irrational/rational beliefs [142]. Issues in the measurement of irrational/rational beliefs has plagued the REBT field with validity and reliability issues widely documented [142, 143]. A review of irrational belief measures conducted in 2009 concluded that the existing measures should be used with caution given that none demonstrated adequate psychometric properties [144]. More recently, existing measures have been deemed “unsatisfactory” [142, p. 122]. Thus, we reaffirm calls to action to develop valid, reliable and robust measurements of irrational/rational beliefs to assess, affirm and advance the theory of REBT as well as accurately measuring client progress [142, 144].

### Methodological quality

The methodological quality across domains (except the sport and exercise domain) was generally comparable, scoring below 50% on our measure of quality. The methodological quality for sport and exercise was approximately 70% thereby indicating higher quality. The methodological quality for randomised control trials and non-randomised control trials was also around 50% with mixed methods studies scoring 93% thereby suggesting higher quality. Accordingly, those developing interventions should look to enhance the rigour, validity and reliability of their interventions.

### Participant experiences

Social validation methods have been used to supplement statistics by revealing details on effective intervention components, experiences of the intervention and outcome change [145]. Our review revealed that social validation is often employed for studies within the sport and exercise domain and those that adopt single case designs [117]. The findings revealed that the interventions increased participants’ ability to manage adversity and contributed to the development of a rational philosophy which transferred to other facets of their life. Performance outcomes were mixed, and some participants would have preferred a longer intervention. Some studies sought the views of the participants [e.g., 84] as well as the views of those operating near the participant (e.g., the athlete’s coach [118]) to enrich and complement the objective data. It would be useful for researchers and practitioners to adopt social validation methods as routine practice where possible and triangulate these findings with other notable individuals related to the participant. To illustrate, the views of parents and teachers could be a useful source of additional information. If social validation methods are not incorporated, we miss out on uncovering acceptability and effectiveness data [15, 117].

### Lessons learned and ways to move forward

Our review has enabled us to bring to the forefront several pressing issues, which if acted upon, can improve the scientific soundness and rigour of REBT intervention research and practice. Thus, we propose guidelines for the current and next generation of researchers/practitioners to adopt, which serve to address the persistent challenges within our field (see Table 1).

**Table 1:** Challenges and best practice guidelines to the implementation of REBT interventions in research and practice.

Challenge	Best Practice Guidelines
Theoretical	Consistent and accurate labelling of REBT interventions in all discourse
	All components of the GABCDE framework to be utilised
Methodological related to measurement	Inclusion of a valid and reliable measure of irrational/rational beliefs
	Increased frequency in measuring rational beliefs
	Increased frequency in measuring rational beliefs
	Avoid making amendments to validated measurements and clearly indicate if amendments are made
	Development of a “gold standard” measure of irrational/rational beliefs
Homework not reported/included or detailed	Inclusion of regular innovative homework using a variety of methods
Lack of technology used	Introduce forms of technology to complement and contribute to clinician/practitioner delivery
Poor reporting of key components (e.g., participants, intervention)	Accurate, transparent and complete reporting which may be documented in supplementary materials
Lack of social validation data	Inclusion of social validation methods
Multimodal intervention studies omit REBT-only comparison groups	Inclusion of REBT-only comparison group

Future REBT intervention research is needed that upholds scientific integrity and robustness. Indeed, the development of a robust measure of irrational/rational beliefs, longitudinal assessments and well controlled interventions would serve to enhance rigour and impact of REBT.

### Limitations

One limitation of the systematic review relates to the poor reporting of research and intervention characteristics across studies which may have negatively impacted our synthesis, critique and methodological appraisal. Of note, we strived to lessen this limitation by contacting authors for further details and adherence to the PRISMA guidelines. Second, a meta-analysis was not possible due to the highly variable methods, samples and statistical approach. Third, we experienced challenges in locating articles (*n* = 49 not retrievable); a large number of these studies were not published in English (*n* = 39). Finally, we have only included peer-reviewed studies in this review, but acknowledge the presence and value of grey literature, which could offer further insights into the efficacy and effectiveness of REBT interventions.

## Conclusion

Our unique and insightful systematic review has determined the effectiveness and efficacy of REBT interventions on irrational and rational beliefs (i.e., mechanisms of change) as well as a range of additional outcomes including mental health. Furthermore, we reveal novel findings that highlighted how most REBT intervention studies were successful in reducing irrational beliefs, increasing rational beliefs and improving additional mental health outcomes, such as depression and anxiety. Successful interventions were characterised by a longer duration (i.e., > 4 weeks), delivered by trained REBT practitioners, adopted the ABC framework and involved daily homework. Methodological quality within the Sport and Exercise domain was assessed as good, whilst all other domains were considered low in quality. By adhering to our best practice guidelines, researchers and practitioners can further enhance the scientific status of REBT.

## Supporting information

S1 File Search strategy. (DOCX)

S2 File Summary of study characteristics. (DOCX)

S3 File Methodological appraisal. (DOCX)

S4 File References included in the systematic review. (DOCX)
