# Supplementary material for: A systematic review of the nature and efficacy of Rational Emotive Behaviour Therapy interventions
Source: PLoS One. 2024 Jul 9;19(7):e0306835. doi: 10.1371/journal.pone.0306835 (PMC11232995; doi:10.1371/journal.pone.0306835)
Supplement: S1 File — (DOCX) [file pone.0306835.s001.docx]

**S1 File**

**Search Strategy**

**Table 1**

*PubMed Population, Intervention, Comparison and Outcomes (PICO) Search Strategy*

| **#** | **Search Terms** |
| --- | --- |
| #1 | “Psychotherapy, Rational Emotive” [MeSH Terms] |
| #2 | “rational” |
| #3 | #1 OR #2 |
| #4 | (“therapy” OR “emoti*” OR “intervention*” OR “counsel*” OR “education*” OR “effectiveness*” OR “behav*” OR “coach*” OR “RT” OR “RET” OR “RBC” OR “REBT” OR “Ellis” |
| #5 | #3 OR #4 |
| #6 | (“irrational belief*” OR “irrational thought*” OR “irrational think*” OR “dysfunction* belief*” OR “dysfunction* thought*” OR “dysfunction* think*” OR “maladaptive belief*” OR “maladaptive thought*” OR “maladaptive think*” |
| #7 | “Mental Disorders” [MeSH Terms] |
| #8 | “Mental Health” [MeSH Terms] |
| #9 | “perform*” OR “behav*” OR “mental wellbeing” OR “mental well being” OR “mental well-being” OR “dysfunction* behav*” OR “maladaptive behav*” OR “distress” OR “psychological distress” |
| #10 | #7 OR #8 OR #9 |
| #11 | #5 AND #6 AND #10 |
| #12 | Filters: 1968 - Present |
| #13 | Filters: Article Type: Journal Article |

**Table 2**

*PsycARTICLES (EBSCOhost) PICO Search Strategy*

| **#** | **Boolean Operator** | **Search Terms** |
| --- | --- | --- |
| #1 | N/A | “Rational Emotive Behavior Therapy” [MeSH Heading] OR “Ellis (Albert)” [MeSH Heading] |
| #2 | OR | “Rational” |
| #3 | AND | “therapy” OR “emoti*” OR “intervention*” OR “counsel*” OR “education*” OR “effectiveness*” OR “behav*” OR “coach*” OR “RT” OR “RET” OR “RBC” OR “REBT” |
| #4 | AND | “irrational beliefs”  [MeSH Heading] OR “irrational thought*” OR “irrational think*” OR “dysfunction* belief*” OR “dysfunction* thought*” OR “dysfunction* think*” OR “maladaptive belief*” OR “maladaptive thought*” OR “maladaptive think*” |
| #5 | AND | “mental health”  [MeSH Heading] OR “mental disorders” [MeSH Heading] OR “well being” [MeSH Heading] OR “behav*” OR “dysfunction* behav*” OR “maladaptive behav*” OR “distress” OR “psychological distress” |
| #6 | N/A | Filters: 1968 - Present |
| #7 | N/A | Filters: Source Types: Peer Reviewed Journals |

**Table 3**

*PsycINFO (EBSCOhost) PICO Search Strategy*

| **#** | **Boolean Operator** | **Search Terms** |
| --- | --- | --- |
| #1 | N/A | “Rational Emotive Behavior Therapy” [MeSH Heading] OR “Ellis (Albert)” [MeSH Heading] |
| #2 | OR | “Rational” |
| #3 | AND | “therapy” OR “emoti*” OR “intervention*” OR “counsel*” OR “education*” OR “effectiveness*” OR “behav*” OR “coach*” OR “RT” OR “RET” OR “RBC” OR “REBT” |
| #4 | AND | “irrational beliefs” [MeSH Heading] OR “irrational thought*” OR “irrational think*” OR “dysfunction* belief*” OR “dysfunction* thought*” OR “dysfunction* think*” OR “maladaptive belief*” OR “maladaptive thought*” OR “maladaptive think*” |
| #5 | AND | “mental health” [MeSH Heading] OR “mental disorders” [MeSH Heading] OR “well being” [MeSH Heading] OR “perform*” OR “behav*” OR “dysfunction* behav*” OR “maladaptive behav*” OR “distress” OR “psychological distress” |
| #6 | AND | Filters: 1968 - Present |
| #7 | N/A | Filters: Source Types: Academic Journals |

**Table 4**

*SPORTDiscus (EBSCOhost) PICO Search Strategy*

| **#** | **Boolean Operator** | **Search Terms** |
| --- | --- | --- |
| #1 | N/A | “Rational” |
| #2 | AND | “therapy” OR “emoti*” OR “intervention*” OR “counsel*” OR “education*” OR “effectiveness*” OR “behav*” OR “coach*” OR “RT” OR “RET” OR “RBC” OR “REBT” OR “Ellis (Albert)” |
| #3 | AND | “irrational belief*” OR “irrational thought*” OR “irrational think*” OR “dysfunction* belief*” OR “dysfunction* thought*” OR “dysfunction* think*” OR “maladaptive belief*” OR “maladaptive thought*” OR “maladaptive think*” |
| #4 | AND | “mental health” [MeSH Heading] OR “mental illness” [MeSH Heading] OR “psychological stress” [MeSH Heading] OR “well-being” [MeSH Heading] OR “perform*” OR “behav*” OR “mental wellbeing” OR “mental well being” OR “mental well-being” OR “dysfunction* behav*” OR “maladaptive behav*” OR “distress” OR “psychological distress” OR “mental disorders” OR “mental health” |
| #5 | AND | Filters: 1968 - Present |
| #6 | N/A | Filters: Source Type: Academic Journals |

**Table 5**

*Scopus PICO Search Strategy*

| **#** | **Search Terms** |
| --- | --- |
| #1 | Rational AND (“therapy” OR “emoti*” OR “intervention*” OR “counsel*” OR “education*” OR “effectiveness*” OR “behav*” OR “coach*” OR “RT” OR “RET” OR “RBC” OR “REBT” OR “Ellis”) AND (“irrational belief*” OR “irrational thought*” OR “irrational think*” OR “dysfunction* belief*” OR “dysfunction* thought*” OR “dysfunction* think*” OR “maladaptive belief*” OR “maladaptive thought*” OR “maladaptive think*”) AND (“perform*” OR “behav*” OR “mental wellbeing” OR “mental well being” OR “mental well-being” OR “dysfunction* behav*” OR “maladaptive behav*” OR “distress” OR “psychological distress” OR “mental health” OR “mental disorders”) AND NOT INDEX(medline) |
| #2 | Filters: 1968 - Present |
| #3 | Filters: Source Type: Journal |
