# Supplementary material for: A systematic review of the nature and efficacy of Rational Emotive Behaviour Therapy interventions
Source: PLoS One. 2024 Jul 9;19(7):e0306835. doi: 10.1371/journal.pone.0306835 (PMC11232995; doi:10.1371/journal.pone.0306835)
Supplement: S2 File — (DOCX) [file pone.0306835.s002.docx]

**S File 2**

**Summary of Study Characteristics**

**Table 6**

*A Summary of Included Studies that Fall Within the Education Domain (n = 59 studies; 35 RCTs and 24 NRCTs)*

| **Study; countr**y | **Design** | **Participants**  N*^a^*, sex (Mage), % retention; type | **Primary outcomes & measure(s)** | **Time-points** | **Additional outcomes** | **Intervention vs control/ comparison** | **Frequency (duration)** | **Mode of delivery** | **Results: Primary outcomes** | **Results: Additional outcomes** |
| --- | --- | --- | --- | --- | --- | --- | --- | --- | --- | --- |
| Arnkoff (1986); USA | RCT | N = 55 males and females, 89% retention  Students (university); Non-clinical | IBs; IBT | 3 time-points: Pre-, post-intervention and 10-week follow-up | Academic performance;  Emotional (anxiety);  Cognitive;  Social validity | Cognitive Restructuring *^e^*  versus Coping versus Wait-list control group | 4 weekly sessions (4 weeks) | Face-to-face group sessions | No significant pre-/post change observed in IBs for Cognitive Restructuring when compared to Coping Programme and wait-list control group. No significant differences in IBs from post to follow-up between intervention groups. | No significant pre-/post change in test and state anxiety, academic performance and thought listing for cognitive restructuring group or between groups. Anxiety decreased from post to follow-up across both intervention groups but no difference between groups. No significant differences from post to follow-up for academic performance between intervention groups. No significant findings for social validation. |
| Boutin and Tosi (1983); USA | RCT | N = 48 females (20 years), retention not reported  Students (university); Non-clinical | IBs; PBI (1) | 3 time-points: Pre-, post-intervention and 8-week follow-up | Academic performance; Emotional (anxiety); Psychophysiological | Rational Stage Directed Hypnotherapy (RSDH) *^e^* versus Hypnosis only versus Attention Placebo versus Wait-list control group | 6 x 1-hr weekly sessions (6 weeks) | Face-to-face group sessions | Significant decreases in IBs from pre to post for RSDH (*d* = 7.56) and Hypnosis (*d* = 2.53) which were maintained at follow-up (RSDH *d* = .28; Hypnosis *d* = .01). No significant findings for Attention Placebo (*d* = .68; follow-up *d* = -.62) or Wait-list control group (*d* = -.40; follow-up (*d* = -.01). | RSDH was significantly more effective than the hypnosis only group in decreasing anxiety, decreasing sweat and increasing academic performance at post test. Findings were maintained at follow-up. |
| Chukwuma et al. (2023); Nigeria | RCT | N = 105 (*n* = 49 males), retention not reported  Students (university); Non-clinical | IBs; ABS-2-AV | 3 time-points: Pre-, post-intervention and 3-month follow-up | Individual differences (ethical sensitivity) | Rational Career Reflective Training (*n* = 52) versus Waitlist control (*n* = 53) | 12 x 1-hr sessions (12 weeks) | Face-to-face group sessions | Significant decreases in irrational beliefs for REBT in comparison to control which was maintained at follow-up. | Significant improvements in ethical sensitivity for REBT in comparison to control which was maintained at follow-up. |
| Cramer and Fong (1991); UK | RCT | N = 60 (*n* = 30 males) (20.25 years), 100% retention  Students (university); Non-clinical | IBs; IBT (modified – only Demand for Approval subscale used) | 2 time-points: Pre- and post-intervention | Emotional | Irrational Self-statements (*n* = 20) versus Rational Self-statements  *^e^* (*n* = 20) versus Neutral Self-statements (*n* = 20) | 15-min one-off session (1 day) | Face-to-face 1:1 sessions | Significant decreases in IBs pre to post for Rational Self Statements (*d* = .31). Significant increases in IBs pre to post for Irrational Self-statements (*d* = .85). No significant findings for neutral self-statements (*d* = .13). | Irrational Self-statements had significantly higher inappropriate and appropriate negative feelings which was significantly greater than Rational and Neutral Self- statement conditions. |
| Crum and College (2016); USA | RCT | N = 47 (*n* = 20 males) (19.22 years), 100% retention  Students (university); Non-clinical | IBs; GABS (modified - Six out of seven subscales used, Other-Downing subscale omitted) | 2 time-points: Pre- and post-intervention | Emotional (anxiety) | Dialectic Rational Beliefs Priming  *^e^* versus No Priming | One-off session (1 day) | Face-to-face group sessions | Significant decreases in IBs pre to post for Dialectic Rational Beliefs Priming (*d* = 1.10) compared to No Priming condition (*d* = .47). | The effect of the activating event on state anxiety was not significant for either group. |
| David & David (2022); Romania | RCT | N = 69 (*n* = 34 males) (10.10 years), retention not reported  Students (children); Non-clinical | RBs & IBs; E-RIBS | 2 timepoints; Pre- and post-intervention | Affect (anxiety and positive emotions) | roboRETMAN *^e^* (n = 24) versus Psychological Pills  *^e^* (*n* = 19) versus Waitlist control group (*n* = 26) | RoboRETMAN = 12 minute episodes. No information regarding other interventions. | Face-to-face group sessions | Significant improvements in IBs in roboRETman group (*d* = .82; Psychological Pills *d* = -.15; Waitlist Control *d* = -.24). Significant improvements in RBs from pre to post in roboRETman group *(d* = -.87; Psychological Pills *d* = -.20; Waitlist Control *d* = -.06). | Significant changes in positive emotions from pre to post for roboRETMAN group only. |
| David et al. (2019); Romania | RCT | N = 165 males and females (10 - 16 years), 86% retention  Students (children); Non-clinical | IBs; CASI | 3 time-points: Pre-, mid- and post-intervention | Cognitive; Emotional | Rational Emotive Behaviour Education *^e^* (*n* = 55) versus REThink *^e^* (*n*  = 54) versus Wait-list control group (*n* = 56). | Rational Emotive Behaviour Education and REThink intervention: 7 x 50 min modules twice per week (1 month) | Rational Emotive Behaviour Education: Face-to-face group sessions. REThink: computer-based group sessions | Significant decrease of irrational cognitions from pre to post for REThink group only (*d* = .59). No significant differences for Rational Emotive Behaviour Education *d* = .26) or Wait-list control group *d* = .11). | Significant decreases in emotional symptoms and depressed mood from pre to post for the REThink group only. |
| Decker and Russell (1981); USA | RCT | N = 30 (*n* = 16 males) (19 years), 85% retention  Students (university); Non-clinical | Irrational Cognitions; PBI (1) | 3 time-points: Pre-, post-intervention and 10-week follow-up | Academic performance; Behavioural; Emotional (anxiety); Psychophysiological | Cue-controlled Relaxation + Cognitive Restructuring *^e^* (n = 10) versus Study-skills Counselling (*n* = 10) versus Wait-list control group (*n* = 10) | 4 x 90-min weekly sessions (4 weeks) | Face-to-face group sessions | Significant decreases in irrational thinking from pre to post for Cue-controlled Relaxation + Cognitive Restructuring (*d* = .21)  and Study-skills Counselling (*d* = .42) compared to wait-list control group (*d* = -.52). Results were maintained at follow-up (Cue-controlled Relaxation + Cognitive Restructuring *d* = .27; Study-skills Counselling *d* = 1.07). | Significant decreases in debilitative test anxiety from pre to post for Cue-controlled Relaxation + Cognitive Restructuring and Study-skills Counselling compared to wait-list control group. Significant improvements in study skills and state anxiety from pre to post for Cue-controlled Relaxation + Cognitive Restructuring group only. Significant decreases in physiological anxiety from pre to post for Study-skills Counselling only. Follow-up results not clearly reported. |
| Ede et al. (2021); Nigeria | RCT | N = 56 (*n* = 30 males), 100% retention  Students (children) living with blindness; Non-clinical | RBs and IBs; ABS-2-AV | 3 time-points: Pre-, post-intervention, 1-month follow-up | Values | Value-based Rational Emotive Behaviour Programme  *^e^* (*n* = 28) versus No-contact control group (*n* = 28) | 12 x 1-hr weekly sessions (12 weeks) | Not reported | Significant decreases in IBs from pre to post for Value-based Rational Emotive Behaviour Programme (*d* = 8.99)  which was maintained at follow-up (*d* = .22). No-contact control group findings not reported clearly but reductions observed in IBs from pre to post (*d* = 13.64) which were maintained at follow-up (*d* = .53). | Significant increases in personal values scores from pre to post for Value-based Rational Emotive Behaviour Programme only compared to controls. Follow-up results not clearly reported. |
| Horan (1996); USA | RCT | N = 56 participants (*n* = 28 males) (16-19 years), 98% retention  Students (adolescents); Non-clinical | IBs; IBT | 2 time-points: Pre- and post-intervention | Self-perception (self-esteem) | Computer based Cognitive Restructuring  *^e^* versus Relaxation Training | 2 x 50 min sessions (2 consecutive days) | Computer-based group sessions | Significant decreases in IBs for Computer based Cognitive Restructuring (*d* = .55) and control group (*d* = .24) from pre to post which was greater for Computer based Cognitive Restructuring. | Significant increases in self-esteem for both groups from pre to post which was greater for Computer based Cognitive Restructuring. |
| Hymen  and  Warren (1978); USA | RCT | N = 11 (*n* = 3 males) (18-25 years), 91% retention  Students (university); Non-clinical | IBs; IBT | 3 time-points: Pre-, post-intervention, 1-month follow-up | Academic performance; Cognitive; Emotional (anxiety, worry) | RET + REI (*n* = 6) versus RET only (*n* = 5) | 6 x 1-hr group sessions (3 weeks) | Face-to-face group sessions | No significant findings in IBs for both conditions. When the groups were combined, significant decreases in IBs from pre to post were observed which was maintained at follow-up. | No significant findings in additional outcomes for both conditions. When the groups were combined, significant decreases in anxiety, worry, emotionality, social evaluative anxiety from pre to post were observed which was maintained at follow-up. When the groups were combined, significant increases in performance from pre to post which was maintained at follow-up. |
| Ifeanyieze et al. (2021); Nigeria | RCT | N = 61 (*n* = 25 males (16 - 24 years), 100% retention  Students (university); Non-clinical | RBs & IBs; ABS-2-AV | 3 time-points: Pre-, post-intervention and 3-month follow-up | Employee behaviours | REBT (*n* = 30) versus No-contact control group (*n* = 31) | 12 sessions (12 weeks) | Face-to-face group sessions and online group sessions | Significant decreases in IBs from pre to post for REBT group (*d* = 4.45) which was maintained at follow-up (*d* = .44). No significant findings for control group from pre to post (*d* = .12) or follow-up (*d* = .08). | Negative relationship between IBs and career engagement. |
| Jalali et al. (2014); Iran | RCT | N = 60 males and females (20-40 years), 100% retention  Students with late blindness (university); Non-clinical | IBs; IBT | 3 time-points: Pre, post-intervention and 1-month follow-up | Emotional (anxiety, depression, stress); Self-perceptions (self-esteem) | REBT (*n* = 30) versus Control group (*n* = 30) | Not reported | Face-to-face group sessions | Significant decreases in IBs for REBT group (*d* = 2.85) which was not maintained at follow-up (*d* = .04). No significant findings for Control group from pre to post (*d* = .03) or follow-up (*d* = .09). | Significant increases in self-esteem and significant decreases in depression, anxiety and stress for REBT group in comparison to control group which was not maintained at follow-up. |
| Leaf et al. (1986); USA | RCT | N = 83 males and females, retention not reported  Students (university); Non-clinical | IBs; SR | 2 time-points: Pre- and post-intervention | Emotional (anger); Physical health | RET (*n* = 83) | 4 sessions (duration not reported) | Face-to-face group sessions | Significant decreases in IBs from pre to post. | Significant decreases in anger and improvements in general health from pre to post. |
| Mahfar et al. (2014); Malaysia | RCT | N = 90, retention not reported  Students (children); Non-clinical | IBs; SBI | 2 time-points: Pre- and post-intervention | Emotional (stress) | REE (*n* = 60) versus Control group (*n* = 30) | 9 sessions (duration not reported) | Face-to-face group sessions | Significant decreases in IBs from pre to post for REE (Group 1 *d* = 5.68; Group 2 *d* = 5.29). Significant decreases in IBs from pre to post for REE compared to control group (*d* = .04). | Significant decreases in stress from pre to post for REE. Significant decreases in stress from pre to post for REE compared to control group. |
| Miller and Kassinove (1978); USA | RCT | N = 96, retention not reported  Students (children); Non-clinical | IBs; II | 2 time-points:  Pre- and post-intervention | Emotional (anxiety); Neuroticism | REE  *^e^* versus REE + Behaviour Rehearsal  *^e^* versus REE + Behaviour Rehearsal + Written Homework  *^e^* versus No-contact control group | 12 x 1-hr weekly (12 weeks) | Face-to-face group sessions | Significant decreases in IBs from pre to post for all REE groups in comparison to no-contact control group. Greatest improvements observed for REE + Behaviour Rehearsal + Written Homework. | Significant decreases in neuroticism and anxiety for REE + Behaviour Rehearsal + Written Homework. |
| Möller et al. (1993); South Africa | RCT | N = 141 (*n* = 41 males) (20.4 years), retention not reported  Students (undergraduate); Non-clinical | IBs; IBT  Irrational Cognitions Related to Self-concept; SCICS | 3 time-points:  Pre- and post- intervention and 6-week follow-up | Behavioural; Self-perceptions (self-concept and esteem) | RET  *^e^* (*n* = 35) versus Subliminal Stimulation (*n* = 34) versus RET + Subliminal Stimulation  *^e^* (*n* = 34) versus Placebo treatment (*n* = 38) | RET: 8 x 60-90 min twice weekly sessions (4 weeks).  Subliminal Stimulation: 8 x 30 min twice weekly sessions (4 weeks). | Face-to-face group sessions | Significant decreases in IBs from pre to post and at follow-up for RET and RET + Subliminal Stimulation in comparison with Subliminal Stimulation and placebo treatment. | Significant improvements in self-concept (except for the behavioural dimension measured by peers), self-esteem and anxiety from pre to post for RET and RET + Subliminal Stimulation in comparison with Subliminal Stimulation and placebo treatment. At follow-up, significant improvements in self-esteem for RET + Subliminal Stimulation and RET in comparison to Subliminal Stimulation and Placebo treatment. Significant improvements in self-concept from post to follow-up for RET + Subliminal Stimulation only. At follow-up, maintained anxiety reductions for RET + Subliminal Stimulation only. |
| Nielsen et al. (1996); USA | RCT | N = 50 (*n* = 20 males) (16.67 years), 88% retention  Students (adolescents); Non-clinical | IBs; IBT | 2 time-points:  Pre- and post-intervention | Self-perceptions (self-esteem) | REBT with a self-esteem focus versus REBT without a self-esteem focus | 4 x 50 min sessions (duration not reported) | Face-to-face group sessions | Significant decreases in IBs from pre to post for both groups. | Significant increases in self-esteem from pre to post for both groups. |
| Ofoegbu et al. (2020); Nigeria | RCT | N = 60 (*n* = 36 males) (15.27 years), 100% retention  Adolescents; Non-clinical | IBs; CASI | 3 time-points:  Pre-, post-intervention and 8-month follow-up | Emotional (depression) | Rational Digital Storytelling *^e^* (*n* = 30) versus Depression Treatment Programme (*n* = 30) | 24 sessions, delivery of twice weekly (12 weeks) | Face-to-face group sessions | Significant decreases in IBs from pre to post for Rational Digital (*d* = 6.64) Storytelling programme which was maintained at follow-up (*d* = .54). Significantly greater reductions for Rational Digital Storytelling programme in comparison to Depression Treatment Programme (pre to post *d* = .02; follow-up *d* = .06). | Significant decreases in depression from pre to post for Rational Digital Storytelling programme. Significantly greater reductions for Rational Digital Storytelling programme in comparison to Depression Treatment Programme. At follow-up, greater maintenance of decreased depression for Rational Digital Storytelling programme in comparison to Depression Treatment Programme. |
| Ogbuanya et al. (2018); Nigeria | RCT | N = 173 (*n* = 120 males) (18.41 years), 100% retention  Students (university); Non-clinical | IBs regarding careers; CBPS-2 | 4 time-points:  Pre -, post-intervention, 2-month follow-up and 6-month follow-up | N/A | REBT (*n* = 87) versus Wait-list control group (*n* = 86) | 20 x 80 min sessions (12 weeks) | Face-to-face group sessions | Significant decreases in irrational career beliefs from pre to post for REBT (*d* = 4.62) which was not maintained at follow-ups (post to follow-up 1 *d* = -.17; post to follow-up 2 *d* = -.27). Compared to waitlist control (*d* = -.26), REBT group had significantly reduced irrational career beliefs from pre to post. | N/A |
| Onyemaechi et al. (2023); Nigeria | RCT | N = 110 (*n* = 55 males), 100% retention  Students (children); Non-clinical | IBs; CASI | 3 timepoints: Pre, post and 3- month follow-up | Individual differences (self-esteem) | REBT (*n* = 55) versus Waitlist control (*n* = 55) | 12 x 1-hr sessions (12 weeks) | Face-to-face group sessions | Significant decreases in irrational beliefs for REBT in comparison to control which was maintained at follow-up. | Significant increases in self-esteem beliefs for REBT in comparison to control which was maintained at follow-up. |
| Popa et al. (2022); Romania | RCT | N = 77 males and females (age information not provided), 83% retention  Students (university); Sub-clinical | RBs; USAQ | 2 timepoints; Pre- and post-intervention | Affect (anxiety, worry); Cognitive (automatic thoughts, dysfunctional attitudes) | CBT *^e^* (*n* = 38) versus Integrative and Multimodal CBT augmented with VR *^e^* (*n* = 39) | Both: 10 x 1-hour weekly sessions | Face-to-face group sessions  IM-VRCBT: addition of virtual reality | Significant increases in unconditional self-acceptance from pre to post for CBT (*d* = -1.82)  and Integrative and Multimodal CBT augmented with VR (*d* = -1.02). No significant differences observed between groups. | Significant decreases in worry, anxiety and dysfunctional thoughts from pre to post for both groups. No significant differences observed between groups. |
| Roman (2011); Romania | RCT | N = 52 (*n* = 4 males) (20.63 years), 100% retention  Students (university); Non-clinical | RBs and IBs; SGABS | 2 time-points:  Pre- and post-intervention | Cognitive; Emotional (anger, anxiety, depression & distress); Religious orientation | REBT (*n* = 19) versus Religiously Oriented REBT (*n* = 18) versus Control group (*n* = 15) | 8 x 1.3-hr twice weekly sessions (4 weeks) | Face-to-face group sessions | Significant decreases in IBs from pre- to post for REBT (*d* = 2.74) and Religiously Oriented REBT (*d* = 2.72) in comparison to control group (*d* = .84). | Significant decreases in depression, anxiety, anger and distress from pre- to post for both REBT groups in comparison to control group. Similar efficacy for REBT and Religiously Oriented REBT. |
| Rosenbaum et al. (1991); Australia | RCT | N = 36 females (9.08 years), 89% retention  Students; Non-clinical | Rationality; CSRB-B | 3 time-points: Pre-, post-intervention and six-month follow-up | Emotional (anxiety); Self-perceptions (locus of control) | REE (*n* = 22) versus Attention Control (*n* = 14) | 10 x 55 min weekly sessions (14 weeks) | Face-to-face group sessions | Significant increases in rational beliefs from pre- to post for REE (internal locus of control *d* = -1.32; external locus of control *d* = -1.40) in comparison to Attention Control (internal locus of control *d* = -.26; external locus of control *d* = .30). At follow-up, there were no differences between the groups (REE internal locus of control *d* = -.06; REE external locus of control *d* = .23; Attention Control internal locus of control *d* = -.46; Attention Control external locus of control *d* = -.69). | Significant increases in perception of internal control from pre- to post for REE in comparison to Attention Control which were maintained at follow-up. No significant decreases in anxiety for REE or Attention Control at post or follow-up. |
| Sahin and Türk (2021); Turkey | RCT | N = 29 participants (*n* = 14 males) (14-15 years), retention not reported  Students; Non-clinical | IBs; IBS (2) AF | 3 time-points:  Pre-, post-intervention and 5- month follow-up | Emotional (wellbeing); Resilience | Cognitive Behavioural Psycho-education Program *^e^* (*n* = 15) versus No-contact control (*n* = 15) | 10 weekly sessions (10 weeks) | Face-to-face group sessions | Significant decreases in IBs from pre- to post for Cognitive Behavioural Psycho-education Program in comparison to control group which was maintained at follow-up. | Significant increases in resilience from pre- to post for Cognitive Behavioural Psycho-education Program in comparison to control group which was maintained at follow-up. No statistically significant findings observed for subjective wellbeing at post or follow-up. |
| Sheehy and Horan (2004); USA | RCT | N = 22 (*n* = 6 males) (median age 30), retention not reported  Students (university); Non-clinical | IBs; IBT | 2 time-points: pre- and post-intervention | Academic performance; Emotional (anxiety, stress) | Stress Inoculation Training *^e^* (*n* = 11) versus Wait-list control *(n* = 11) | 4 x 1.5 weekly sessions (4 weeks) | Face-to-face group sessions | Significantly greater decreases in IBs from pre to post for Stress Inoculation Training (*d* = .49) in comparison to control (*d* = .44). | Significant decreases in stress and anxiety from pre- to post for Stress Inoculation Training only. Significant increases in academic performance from pre- to post for Stress Inoculation Training only. |
| Sousa and Padovani (2021); Brazil | RCT | N = 25 (*n* = 1 males) (19.68 years), 100% retention  Students (university); Non-clinical | IBs; IBQ | 3 time-points: Pre-, post-intervention and 6-week follow up | Emotionality (affect); Life satisfaction; Social skills; Social validity | REBT (*n* = 12) versus Psychoeducation (*n* = 9) versus Control (*n* = 4) | 10 x 1-hr weekly sessions (10 weeks) | Face-to-face group sessions | Significant decreases in IBs from pre to post for REBT (*d* = .84), Psychoeducation (*d* = .92) and Control (*d* = .00)  which was maintained for control (*d* = .42) at follow-up (REBT *d* = .00; Psychoeducation *d* = -.47). | Significant differences in social skills and affect from pre to post to follow-up for all three groups (not clear which group improved the most). No significant findings observed for wellbeing or satisfaction. Participants reported that cognitive change and generalisation of learning to other environments. They also reported that the meetings were ‘small’. |
| Tomoiagã et al. (2022); Romania | RCT | N = 139 (*n* = 17 males) (26.27 years), 89% retention  Students (university); Non-clinical | RBs & IBs; ABS-2-AV | 3 timepoints;  Pre-, mid- and post-intervention | Affect (emotional distress) | Game-based CBT with guidance *^e^* (n = 46) versus Game-based CBT without guidance *^e^* (*n* = 44) versus Control group (*n* = 49) | 3 x 10-20 minute levels (≈ 4.5 days) | Online | No significant change from pre to post for irrational (Game-based CBT with *d* = 1.05; Game-based CBT without guidance *d* = .87; Control group *d* = .41) or rational beliefs (Game-based CBT with *d* = -.51; Game-based CBT without guidance *d* = -.55; Control group *d* = -.11) for any groups although means demonstrated improvement. | Significant decreases in negative dysfunctional emotions from pre to post for both groups in comparison to control. No significant findings between groups. |
| Trexler and Karst (1972); USA | RCT | N 33 (*n* = 16 males), 85% retention  Students (university); Non-clinical | IBs; IBT | 2 time-points: Pre- and post-intervention | Emotional (anxiety), Psychophysiological; Public speaking confidence; Social validity | RET (*n* = 11) versus Attention Placebo (*n* = 10) versus No treatment (*n* = 12) | 4 sessions (duration not reported) | Face-to-face group sessions | Significant decreases in IBs from pre- to post for RET in comparison to Attention Placebo and no-treatment. | Significant increases in public speaker confidence and improved anxiety from pre- to post for RET in comparison to Attention Placebo and no-treatment. Attention Placebo was significantly superior than RET and no-treatment group for anxiety. Participants reported that ‘treatment fell somewhat short’. |
| Türkum (2007); Turkey | RCT | N = 29 (*n* = 14 males) (20.54 years) 83% retention  Students (university); Non-clinical | IBs; IBS (2) | 2 time-points: Pre- and post-intervention | Coping skills; Optimism | Coping with Stress Counselling Training *^e^*  (*n* = 10) versus Coping with Stress Guidance Training *^e^*  (*n* = 10) versus Wait-list control (*n* = 9) | 8 x 90 min weekly sessions (8 weeks) | Face-to-face group sessions | Significant decreases in IBs from pre to post for Coping with Stress Counselling Training (*d* = 1.13) and Coping with Stress Guidance Training (*d* = 1.24). No significant findings for control group (*d* = -.01). | Significant increases in optimism from pre to post for Coping with Stress Counselling Training. Significant increases in problem-focused stress coping from pre to post for Coping with Stress Guidance Training. Significant decreases in use of avoidance strategies for coping with stress from pre to post for control group. |
| Ugwoke et al. (2021); Nigeria | RCT | N = 581 (*n* = 316 males) (39.22 years), 98% retention  Students (university); Non-clinical | IBs; PTIBQ | 4 time-points: pre-, post-intervention, 2-month follow-up and 4-month follow-up | Occupational risks | REBT (*n* = 286) versus Wait-list control (*n* = 295) | ≈70 x 2-hr sessions, delivery of twice weekly (8 months) | Face-to-face group sessions | Significant improvements in IBs from pre to post for REBT (*d* = -4.28)  in comparison to wait-list control group (*d* = -.30) which was maintained at follow-up for REBT group (follow-up 1 *d* = -1.56; follow-up 2 *d* = -1.95). | Significant improvements in emotional distress and risk management practices from pre to post for REBT in comparison to wait-list control group which was maintained follow-up. |
| Victor-Aigbodion et al. (2023); Nigeria | RCT | N = 90 (*n* = 48 male), 17 - 28 age range, 100% retention  Students (university); Sub-clinical | Irrational beliefs; IBI | Pre-, post intervention and 2-week follow-up | Mental health (depression) | REBT (*n* = 45) versus Placebo (*n* = 45) | 20 x 50 min sessions (12 weeks) | Face-to-face group sessions | Significant decreases in irrational beliefs for REBT in comparison to control from pre to post (*d* = 3.42) which was maintained at follow-up (*d* = 3.45). | Significant decreases in depression for REBT in comparison to control which was maintained at follow-up. |
| Warren et al.  (1984); USA | RCT | N = 59 (12-16 years), retention  not reported  Students (adolescents); Non-clinical | IBs; II | 3 time-points: Pre-intervention, post-intervention and 3-week follow-up | Cognitive; Emotional (anxiety); Social skills | RET (*n* = 14) versus RET + REI (*n* = 14) versus Relationship-  oriented Counselling (*n* = 16) versus Wait-list control (*n* = 15) | 7 x 50-minute 2-3 times per week (3 weeks) | Face-to-face group sessions | Significant decreases in IBs from pre- to post-intervention for RET and RET + REI compared to Relationship-oriented Counselling and wait-list control. Significant findings maintained at follow-up for RET + REI only. No significant findings between-groups. | Significant decreases in interpersonal anxiety from pre- to post-intervention for RET and RET + REI compared to Relationship-oriented Counselling and wait-list control. Findings were maintained at follow-up. |
| Wessel and Mersch (1994); Netherlands | RCT | N = 41 (*n* = 19 males) (16.5 years), 100% retention  Students (adolescents); Non-clinical | IBs; RBI | 2 time-points: Pre- and post-intervention | Emotional (anxiety); Mental ill-health (general psychopathology); Social skills | RET + Exposure (*n* = 22) versus Wait-list control (*n* = 19) | 10 x 2-hr weekly sessions (10 weeks) | Face-to-face group sessions | Significant decreases in IBs from pre- to post for RET + Exposure (*d* = -2.17) in comparison to wait-list group (*d* = -1.23). | Significant improvements in anxiety and social skills from pre- to post for RET + Exposure in comparison to wait-list group. |
| Xu and Liu (2017); China | RCT | N = 60 females (22 years), 100% retention  Students (university); Non-clinical | IBs; IBQ-40 | 2 time-points:  Pre and post-intervention | Mental ill-health | REBT (*n* = 25) versus Neurofeedback (*n* = 35). | 8 x 35-45 min weekly sessions (8 weeks) | Face-to-face group sessions | Increase in adjusted IBs from pre to post for REBT group (*d* = -.67). No increases observed for neurofeedback group (*d* = .03). No statistical analysis reported. | Decreased mental ill-health symptoms from pre to post in the REBT group and increased symptoms in the neurofeedback group. No statistical analysis reported. |
| Bedel et al. (2020); Turkey | NRCT | N = 26 (*n* = 13 males), 100% retention  Students (children); Non-clinical | IBs; IBS-A | 2 time-points: Pre- and post-intervention | Emotional (anxiety) | Test Anxiety Psychoeducation Programme *^e^*  (*n* = 13) versus Control group (*n* = 13) | 10 x 50min weekly sessions (10 weeks) | Face-to-face group sessions | Significant decreases for IBs from pre to post for REBT group (*d* = .98) compared to control group (*d* = .06). | Significant decreases in anxiety from pre to post for REBT group compared to control group. |
| Boutin (1978); USA | NRCT | N = 1 female (17 years), 100% retention  Student (university); Sub-clinical | IBs; PBI (1) | 2 time-points: Pre- and post-intervention | Academic performance; Emotional (anxiety); Hypnotic susceptibility; Psychophysiological | Rational Stage Directed Hypnotherapy *^e^*  (*n* = 1) | 8 x 1-hr weekly (8 weeks) | Face-to-face 1:1 sessions | Decreased irrational cognitions from pre to post. Of note, no statistical analysis was conducted. | Decreased anxiety (also by sweat) and increased academic performance from pre to post which was maintained at follow-up. Of note, no statistical analysis was conducted. |
| Caruso et al. (2018); Italy | NRCT | N = 237 (*n* = 108 males) (47.5 years for teachers and 9 years for students), retention not reported  Teachers and students (children); Non-clinical | RBs; CSRB-B (only completed by the students) | 2 time-points: Pre- and post-intervention | Self-perceptions (self-efficacy) | REE for teachers and students (*n* = 76) versus REE for students only (*n* = 86) versus Control group (n=75) | REE for teachers and students: 8 x 1-hr sessions, delivery of 2 sessions per month (5 months).  REE for students only: 8 x 1-hr weekly sessions (2 months) | Face-to-face group sessions | Significant increases of rational beliefs in children for REE for teachers and students (*d* = -.64)  and REE for students only (*d* = -1.17)  from pre to post compared to control group (*d* = -.09)  . No significant differences between the REE groups. | Significant increases of self-efficacy in teachers for REE delivered to teachers and students from pre to post which was not observed in the REE for students only or control group. |
| Cristea et al. (2006; 2008) *^c^*; Romania | NRCT | N = 63 (*n* = 40 males) (9-10 years), 100% retention  Students (children); Non-clinical | IBs; CASI | 2 time-points: Pre- and post-intervention | Emotional (anxiety); REBT knowledge | Rational Emotive and Behavioural Education (*n* = 18) versus Astronomy (*n* = 23) versus Control group (*n* = 22) | 20 x 45 min twice weekly sessions (3-4 months) | Face-to-face group sessions | No significant findings in IBs for Rational Emotive and Behavioural Education (*d* = .42) compared to Astronomy (*d* = .59) and Control group (*d* = -.16). | Significant increases in REBT knowledge for Rational Emotive and Behavioural Education compared to the other conditions. No significant findings in anxiety for Rational Emotive and Behavioural Education compared to other groups. |
| David and Cobeanu (2016); Romania | NRCT | N = 102 (*n* = 19 males) (36.94 years), 86% retention  Students (university); Non-clinical | RBs & IBs; SGABS | 2 time-points: Pre- and post-intervention | Emotional (distress); Employee performance | Cognitive Behavioural Coaching (*n* = 102) | No frequency reported (6 months) | Face-to-face group sessions | Significant decreases in IBs from pre to post (*d* = .54). No significant findings for rational beliefs (*d* = -.03) | Significant decreases in depression and significant increases in work performance from pre to post. No significant change in general distress. |
| David et al. (2021); Romania | NRCT | N = 31 (*n* = 13 males) (12.25 years), retention not reported  Students (children); Sub-clinical | RBs & IBs; ABS-2-S.  IBs; CASI | 2 time-points: Pre- and post-intervention | Cognitive; Emotional; Social validity | REThink Therapeutic Online Video Game *^e^* (*n* = 31) | 3 x 1-hr weekly sessions (3 weeks) | Computer-based 1:1 sessions (accessed at own convenience) | Significant decreases in IBs from pre to post (ABS-2-S *d* = .59; CASI *d* = .31). No significant findings for rational beliefs (*d* = -.23). | Significant improvements in emotional problems and negative automatic thoughts from pre to post. No significant findings for problem solving abilities. Participants reported high levels of intervention satisfaction. |
| David et al. (2022); Romania | NRCT | N = 54 (*n* = 18 males) (13 years), retention not reported  Students (children); Non-clinical | IBs; CASI | 3 timepoints;  Pre-, mid- and post-intervention. | Affect (functional and dysfunctional mood, emotion regulation); Emotional/behavioural problems in children | REThink Game (*n* = 54) | 7 modules approx. 50 mins each (1 month) | Computer based | Irrational belief change was not reported from pre to post (*d* = .58). Those better at identifying the connection between thinking and feeling reported higher improvements in irrational beliefs areas. | In-game performance at some levels reflected improvements in mental health, tolerance for rules, positive emotions and emotional control. In-game performance at other levels were less associated with reduced depressive mood and lower emotional difficulties improvements. |
| Flanagan et al. (1998); USA | NRCT | N = 44 (*n* = 17 males) (9-11 years), 100% retention  Students (children); Non-clinical | IBs; CASI | 2 time-points: Pre- and post-intervention | Social skills | REBT + Problem Solving (*n* = 22) versus  Problem Solving (*n* = 22) | 12 x 30 min weekly sessions (12 weeks) | Face-to-face group sessions | Greater decreases in IBs for REBT + Problem Solving (*d* = .31 - *d* = .64) from pre to post compared to Problem Solving (*d* = -.17 - *d* = .23). | Greater improvements in social skills for REBT + Problem Solving from pre to post compared to Problem Solving. |
| Jacobs and Croake (1976); USA | NRCT | N = 74 males and females, 85% retention  Students (university); Non-clinical | IBs; AIII | 2 time-points: Pre- and post-intervention | Emotional (anxiety); Problems | RET versus Control group | 5 x 1.5-hr weekly sessions (5 weeks) | Face-to-face group sessions | Increases in rational thinking from pre to post for RET compared to control group evident by mean scores. No further analysis conducted. | Decreases in anxiety and self-reported problems from pre to post for RET group compared to control group evident by mean scores. No further analysis conducted. |
| Kabasakal and Emiroğlu (2020); Turkey | NRCT | N = 212 (*n* = 104 males) (12.02 years), 100% retention  Students (adolescents); Non-clinical | IBs; IBS-A | 2 time-points: Pre- and post-intervention | Emotional (wellbeing); Self-perception (self-efficacy); Social skills; Social validity | RET (*n* = 97) versus Control group (*n* = 115) | 24 x 45 min sessions, delivery of twice weekly (12 weeks) | Face-to-face group sessions | Significant decreases in IBs for RET group from pre to post. No significant findings for control group. | Significant increases in wellbeing, self-efficacy and social acceptance towards disabled students for RET group from pre to post. No significant findings for control group. Parents of the participants reported that the intervention positively influenced the participants. |
| Lupu and Iftene (2009); Romania | NRCT | N = 88 participants (*n* = 53 males) (17.4 years), 100% retention  Students (adolescents); Non-clinical | RBs & IBs; ABS-II | 2 time-points: Pre- and post-intervention | Emotional (anxiety & depression) | REE (*n* = 50) versus Control group (*n* = 38) | One-off 50 min session (1 day) | Face-to-face group sessions | Significant decreases in IBs from pre to post for REE (*d* = -5.10). Significant decreases in IBs from pre to post for REE compared to control group (*d* = -.26). | Significant decreases in anxiety from pre to post for REE. Significant decreases in anxiety from pre to post for REE compared to control group. |
| McCormick et al. (1991); USA | NRCT | N = 35 (*n* = 9 males) (22 years), retention not reported  Students (university); Non-clinical | RBs & IBs; ABS-II | 2 time-points: Pre- and post-intervention | Self-perceptions (self-efficacy) | RET (*n* = 12) versus Social Development (*n* = 8) versus Extra Sensory Perception (*n* = 9) versus Humanistic Psychology (*n* = 6) | 30 x 75 min twice weekly sessions (15 weeks) | Face-to-face group sessions | No significant pre-/post change observed in IBs for RET. | Significant increases in self-efficacy from pre to post for RET in comparison to Social Development and Extra Sensory Perception. |
| Mio and Matsumuto (2018); Japan | NRCT | N = 515 (*n* = 287 males) (12-14 years), 100% retention  Students (secondary school); Non-clinical | IBs; IBS-10 | 2 time-points:  Pre- and post-intervention | Self-perceptions (self-esteem) | Onayami Kaiketsu Shiito (OKS)/Problem-solving Sheet *^e^* (*n* = 238) versus Assertiveness Training programme (*n* = 277) | One-off 50 min session (1 day) | Face-to-face group sessions | Significant decreases in IBs from pre to post for OKS (*d* = 4.48)  in comparison to control group (*d* = 1.94). | No significant pre-/post change observed in self-esteem for any group. |
| Morris (1993); Canada | NRCT | N = 24 (*n* = 19 males) (15.64 years), retention not reported  Adolescents diagnosed with either conduct disorder or ADHD; Clinical | IBs; IBT | 2 time-points:  Pre- and post-intervention | Emotional (anxiety & depression) | REE (*n* = 24) | 20 x 1-hr twice weekly (10 weeks) | Face-to-face group sessions | Significant decreases in IBs from pre to post for students with conduct disorder only. No significant findings for students with ADHD. | Significant decreases in depression, state and trait anger from pre to post for students with conduct disorder only. No significant findings on any variable for students with ADHD. |
| Popa & Predatu (2019); Romania | NRCT | N = 60 (n = 21 males) (20.40 years), retention not reported  Students (university); Sub-clinical | RBs & IBs; ABS-2 | 2 timepoints; Pre- and post-intervention | Affect (affective distress, anxiety); Individual differences (personality) | Integrative CBT/REBT (*n* = 60) | 10 x 50-min weekly sessions (10 weeks) | Face-to-face group sessions | Significant decreases in irrational beliefs from pre to post VR (*d* = .80). | Significant decreases in anxiety and negative emotions from pre to post. Significant increases in emotional stability from pre to post. |
| Schenk et al. (2020); Romania | NRCT | N = 40 (*n* = 7 males) (19.22 years), retention not reported  Students (university); Clinical | RBs & IBs; ABS-2 | 2 time-points: pre- and post-intervention | Emotional (anxiety) | REBT (*n* = 40) | 8 sessions (duration not reported) | Face-to-face. Group or 1:1 format not reported | Significant decreases in IBs from pre to post (*d* = .55). | Significant decreases in anxiety from pre- to post. |
| Thorpe et al. (1984) Study 1; USA | NRCT | N = 27 (*n* = 13 males), retention not reported  Students (university); Non-clinical | IBs; CBS | 2 time-points: Pre- and post-intervention | Cognitive; Social skills; Social validity | REI Behavioural Rehearsal *^e^* versus REI Cognitive  *^e^* versus REI Emotional Rehearsal *^e^* | 2 x 1.5-hrs scheduled fortnightly (4 weeks) | Face-to-face group sessions | Significant decreases in IBs from pre to post for Behavioural Rehearsal (*d* = .56), Cognitive Rehearsal (*d* = .30)  and Emotional Rehearsal (*d* = .45). No difference observed across groups. | Significant improvements in social skills and cognitive outcomes from pre to post across all groups. REI Behavioural Rehearsal was significantly superior to REI Cognitive and REI Emotional Rehearsal for conflict resolution. No difference in likability of practitioner or benefits of intervention across groups. |
| Thorpe et al. (1984) Study 2; USA | NRCT | N = 28 (*n* = 9 males), retention not reported  Students (university); Non-clinical | IBs; CBS | 2 time-points: Pre- and post-intervention | Cognitive; Social skills; Social validity | Behavioural Rehearsal *^e^* versus REI *^e^* versus Behaviour Rehearsal + Emotional Rehearsal *^e^* versus Behavioural Rehearsal + Cognitive Rehearsal  *^e^* | Frequency and duration not reported | Face-to-face. Group or 1:1 format not reported | REI was significantly superior in reducing IBs from pre to post (*d* = .57; Behavioural Rehearsal *d* = .61; Behaviour Rehearsal + Emotional Rehearsal *d* = -.18; Behavioural Rehearsal + Cognitive Rehearsal *d* = .30). | REI was significantly superior in improving assertiveness and cognitive outcomes from pre to post. No difference in likability and competence of practitioner across groups. At post-test, Behavioural Rehearsal + Cognitive Rehearsal reported greater improvements. Mood improved from pre to post for all groups. |
| Thurman (1983); USA | NRCT | N = 22 (*n* = 12 males) (23.5 years), 100% retention  Students (university); Non-clinical | IBs; IBT (modified - 6/10 subscales used as related to Type A behaviour) | 3 time-points: pre-, post-intervention and 2-month follow-up | Emotional (anxiety, happiness & stress); Social validity; Type A personality | RET (*n* = 11) versus No-contact control (*n* = 11) | 6 x 2-hr weekly sessions (6 weeks) | Face-to-face group sessions | Significant decreases in IBs from pre- to post for RET (*d* = 2.00) in comparison to no-contact control (*d* = -.30)  which was maintained/improved at follow-up (RET *d* = .28). | Significant decreases in Type A behaviours from pre- to post for RET group in comparison to no-contact control which was maintained/improved at follow-up. Participants reported the intervention was helpful. Non-significant findings for both groups for emotions. |
| Trip et al. (2010); Romania | NRCT | N = 192 (*n* = 87 males) (10-11 years), retention not reported  Students (children); Non-clinical | IBs; CASI | 3 time-points: Pre-, post-intervention and 1-year follow-up | Emotional; Dysfunctional behaviours; Self-perceptions (locus of control) | Rational Emotive Behavioural Education (*n* = 78) versus Civic Education Classes (*n* = 34) versus control group (*n* = 80) | 13 x 1-hr weekly (13 weeks) | Face-to-face. Group or 1:1 format not reported | Significant decreases in IBs from pre- to post for Rational Emotive Behavioural Education group (*d* = .67)  in comparison to Civic Education Classes (*d* = -.22)  and control group (*d* = .20). At follow-up, unconditional self-acceptance was the only significant finding maintained by Rational Emotive Behavioural Education. | Significant improvements in academic behaviours from pre to post for Rational Emotive Behavioural Education only. Rational Emotive Behavioural Education had significantly lower internal locus of control at post-test when compared to Civic Education Classes group. No significant changes in emotions. Follow-up results not reported. |
| Ulusoy and Duy (2013); Turkey | NRCT | N = 30 (8th grade students), 90% retention  Students (children); Non-clinical | IBs; IBS-A | 2 time-points: Pre- and post-intervention | Cognitive | Cognitive Restructuring  *^e^* (*n* = 10) versus Adolescent Development Placebo (*n* = 10) versus No-treatment control group (*n* = 10) | 10 x 40 min sessions (duration not reported) | Face-to-face group sessions | Significant decreases in IBs from pre- to post for Cognitive Restructuring. No significant findings for control group. | No significant findings for attributional style. |
| Vaida et al. (2008); Romania | NRCT | N = 72 (*n* = 37 males) (18.08 years), 100% retention  Students (adolescents); Non-clinical | IBs; Attitude and Belief Scale II | 2 time-points: Pre- and post-intervention (6 months following intervention) | N/A | Rational Emotive Behaviour Education (n = 48) versus Control group (*n* = 24) | ≈ 52 x 1 hr sessions, delivery of twice weekly (6 months) | Face-to-face. Group or 1:1 format not reported | Significant decreases in IBs from pre- to post for Rational Emotive Behaviour Education (*d* = .65). No significant findings for control group (*d* = .01). | N/A |
| Wilde (1996); Canada | NRCT | N = 95, retention not reported  Students (children); Non-clinical | IBs; II | 2 time-points:  Pre- and post-intervention | Academic performance | REE (*n* = 95) | 4 x 30 min twice weekly sessions (2 weeks) | Face-to-face group sessions | Significant decreases in IBs from pre- to post. | A higher percentage of children who scored below the mean academic performance measure improved from pre- to post than children who scored above the mean. |
| Wilde (1996; 1999) *^b^*; USA | NRCT | N = 153, 77% retention.  Wilde (1999): At follow-up, an additional 58 participants were recruited as a comparison group.  Students (children); Non-clinical | IBs; II | 3 time-points: Pre-, post-intervention and 4-year follow-up | N/A | REE (*n* = 95) | 4 x 30 min twice weekly sessions (2 weeks) | Face-to-face group sessions | Significant decreases in IBs from pre- to post (*d* = -.53) which were maintained at follow-up. At follow-up, those who had received REE had significantly lower IBs than those who had not. | N/A |

*Note:* ^a^N is the initial sample size for the intervention and control condition (if relevant). *^b^* Wilde (1999) is a follow-up study to Wilde. (1996). *^c^* Cristea (2006) and Cristea (2008) is the same study. *^d^* Thorpe et. al. (1984) detailed two separate intervention studies and thus was split into ‘Study 1’ and ‘Study 2’. *^e^* Intervention is based upon REBT principles. *^f^* Waitlist control group then underwent RETMAN & Retmagic cartoons.

General: ADHD: Attention Deficit Hyperactivity Disorder; IBs = Irrational beliefs; NRCT = Non-randomised control trial; RBs = Rational beliefs; RCT = Randomised control trial; REBT = Rational Emotive Behaviour Therapy; REE = Rational Emotive Education; REI = Rational Emotive Imagery; RET = Rational Emotive Therapy; RT = Rational Therapy.

Measures: ABS – 2 = Attitudes and Belief Scale 2; ABS-2-AV = Attitudes and Belief Scale 2-Abbreviated version; ABS-2-S = Attitudes and Beliefs Scale - Short Form; AIII = Adult Irrational Ideas Inventory; CASI = Child and Adolescent Scale of Irrationality; CBPS-2 = Career Belief Patterns Scale Version 2; CBS = Common Beliefs Survey; CSRB-B = Children's Survey of Rational Beliefs Form B; E-RIBS = Exam-Beliefs Scale; GABS = General Attitudes and Beliefs Scale; GABS-II = Attitudes and Belief Scale – 2; IBI = Irrational Beliefs Inventory; IBQ - 40 = Scale Adjusted IBs; IBQ = IBs Questionnaire; IBS-10 = IBs Scale 10; IBS-A = IBs Scale for Adolescents; IBS (2) = The IBs Scale; IBS (2) AF = The IBs Scale: Adolescent Form; IBT = IBs Test; II = The Idea Inventory; PBI (1) = Personal Beliefs Inventory; PTIBQ = Perceptions of Technical Vocational Education Training Image and IBs Questionnaire; RBI = Rational Behaviour Inventory; SBI = Students' Beliefs Questionnaire; SCICS = Self-concept Irrational Cognition Scale; SGABS = General Attitudes and Beliefs Scale- Short Form; SR = Scale of Rationality.

**Table 7**

*A Summary of Included Studies that Fall Within the Forensic Domain (n = 1 studies; 1 RCT)*

| **Study; countr**y | **Design** | **Participants**  N*^a^*, sex (Mage), % retention; type | **Primary outcomes & measure(s)** | **Time-points** | **Additional outcomes** | **Intervention vs control/ comparison** | **Frequency (duration)** | **Mode of delivery** | **Results: Primary outcomes** | **Results: Additional outcomes** |
| --- | --- | --- | --- | --- | --- | --- | --- | --- | --- | --- |
| Aldahadha (2018); Jordan | RCT | N = 83 males and females (29.41 years), retention not reported  Convicted terrorist and extremists; Non-clinical | IBs; BI (modified - only 50 items used) | 2 time-points: Pre- and post-intervention | N/A | Disputing Irrational Beliefs Programme *^e^* (*n* = 44) versus Control group (*n* = 39) | 12 x 1.5-hr weekly sessions (12 weeks) | Face-to-face. Group or 1:1 format not reported | Significant decreases for IBs from pre to post for Disputing Irrational Beliefs Programme (*d* = 2.98) compared to control group (*d* = -.10). | N/A |

*Note:* *^a^* N is the initial sample size for the intervention and control condition (if relevant).

General: RCT = Randomised control trial; IBs = Irrational beliefs.

Measures: BI = Belief Inventory.

**Table 8**

*A Summary of Included Studies that Fall Within the Hospital and Community Healthcare Domain (n = 25 studies; 14 RCTs and 11 NRCTs)*

| **Study; countr**y | **Design** | **Participants**  N*^a^*, sex (Mage), % retention; type | **Primary outcomes & measure(s)** | **Time-points** | **Additional outcomes** | **Intervention vs control/ comparison** | **Frequency (duration)** | **Mode of delivery** | **Results: Primary outcomes** | **Results: Additional outcomes** |
| --- | --- | --- | --- | --- | --- | --- | --- | --- | --- | --- |
| Cramer and Kupshik (1993); UK | RCT | N = 18 females (34.8 years), 100% retention  Outpatients; Sub-clinical | IBs; IBS (1) | 2 time-points: Pre- and post-intervention | Emotional; Mental ill-health (non-psychotic psychiatric disorders) | Rational Self-statements *^d^* (*n* = 9) versus Irrational Self-statements (*n* = 9) | One-off session (1 day) | Face-to-face. Group or 1:1 format not reported | No significant findings for IBs (rational self-statements *d* = .20; irrational self-statements *d* = .10). | Significant decreases in appropriate and inappropriate negative emotions for Rational Self-statements at post. No significant findings for Irrational Self-statements condition. |
| Emmelkamp and Beens (1991); Netherlands | RCT | N = 30 males and females (18-65 years), 70% retention  Adults diagnosed with OCD; Clinical | IBs; IBT | 7 time-points over two blocks of intervention: Pre-intervention block 1, 4-weeks after pre– intervention block 1, post– intervention block 1, pre– intervention block 2, post– intervention block 2,  4-week follow-up and six-month follow-up | Emotional (anxiety); Mental ill-health (OCD) | RET + Exposure in Vivo  *^d^* versus Exposure in Vivo | 12 x 1-hr sessions (8 weeks) | Face-to-face. Group or 1:1 format not reported | Significant decreases in IBs from pre to post for RET + Exposure in Vivo (*d* = .85)  compared to Exposure in Vivo (*d* = .31). Results maintained at follow-up for RET + Exposure in Vivo (*d* = .17) only (Exposure in Vivo *d* = -.06). | Significant decreases in anxiety/discomfort and OCD symptoms from pre to post for both groups. Results maintained at follow-up. |
| Emmelkamp et al. (1986); Netherlands | RCT | N = 51 (36 years), 77% retention  Phobics (agoraphobia); Clinical | IBs; IBT | 4 time-points across two blocks of intervention: Pre-intervention block 1, post-intervention block 1, pre-intervention block 2, post-intervention block 2 | Behavioural; Cognitive; Phobia (clinical improvement, symptoms) | RET + Exposure in Vivo  *^d^* versus Exposure in Vivo versus Self-instructional Training + Exposure in Vivo | 12 x 2.5-hr sessions (duration not reported) | Face-to-face group sessions | Significant decreases in IBs from pre to post for all groups and no significant difference between groups. | Significant improvements on all phobic measures from pre to post for all groups. Exposure in Vivo produced the greatest improvements. RET group had the most treatment failures at post-test. |
| Emmelkamp, Mersch, Vissia and Van Der Helm (1985); Netherlands | RCT | N = 34 (*n* = 13 males) (31 years), 88% retention  Phobics (social); Clinical | IBs; IBT | 3 time-points: Pre-, post-intervention and 1-month follow-up | Emotional (anxiety); Phobia; Psychophysiological | RET versus Exposure in Vivo  *^d^* versus Self-instructional Training | 6 x 2.5-hrs (duration not reported) | Face-to-face group sessions | Significant decreases in IBs from pre to post for RET group. At follow-up significant improvements for IBs for RET group. | Significant improvements on phobic anxiety, social anxiety and symptom checklist for RET group from pre to post. No significant improvements on fear or pulse for RET group. RET was superior to Self-instructional Training on phobic anxiety from pre to post to follow-up. |
| Emmelkamp et al. (1988); Netherlands | RCT | N = 20 males and females (29.9 years), 90% retention  Phobics (OCD); Clinical | IBs; IBT | 4 time-points: Pre-, post-intervention, 1-month and 6-month follow-up | Emotional (anxiety, depression & hostility); Mental ill-health (OCD) | RET versus Self-controlled Exposure | 10 x 1-hr sessions (8 weeks) | Face-to-face group sessions | Decreases in IBs from pre to post for RET (*d* = .69) and at follow-up (*d* = .55). which did not reach significance. No significant findings for Self-controlled Exposure from pre to post (*d* = .05) or at follow-up (*d* = .02). Results at follow-up for individual treatment groups were not assessed. Results were pooled (RET and Self-controlled Exposure) which demonstrated results were maintained. No 6-month follow-up data presented. | Significant decreases in social anxiety from pre to post for both conditions. Significant decreases depression from pre to post for RET group. More patients rated as clinically improved from Self-controlled Exposure group than RET group. Results at follow-up for individual treatment groups were not assessed. Results were pooled (RET and Self-controlled Exposure) which demonstrated results were maintained. |
| Kassinove et al. (1980); USA | RCT | N = 34 (*n =* 12 males) (21 – 56 years), retention not reported  Neurotic clients of a community health centre; Clinical | IBs; II | 2 time-points: Pre- and post-intervention | Emotional (anxiety); Neuroticism | Rational Emotive Bibliotherapy (n = 11) versus Rational Emotive Audio Therapy (*n* = 11) versus No-contact control (*n* = 12) | 16 x 1-hr sessions twice weekly (8 weeks) | Face-to-face. Group or 1:1 format not reported | Significant decreases in IBs for Rational Emotive Bibliotherapy (*d* = -1.34) and Rational Emotive Audio Therapy (*d* = .60) in comparison to no-contact control (*d* = .15) from pre to post. | Significant decreases in neuroticism and trait anxiety for Rational Emotive Bibliotherapy group only from pre to post. |
| Komasi et al. (2017); Iran | RCT | N = 44 *n* = 36 males), 84% retention  Drug addicts undergoing pharmacological therapy; Clinical | IBs; IBT | 2 time-points: Pre- and post-intervention | Social validity | Individual Metaphor Therapy  *^d^* versus Control group | 10 x 1hr sessions (duration not reported) | Face-to-face 1:1 sessions | Significant decreases in IBs from pre to post for Individual Metaphor Therapy (Approval Seeking IB *d* = .69) compared to control group (Approval Seeking IB *d* = .96). | Participants reported that the content was pleasant and remarkable. |
| Lipsky et al. (1980); Smith (1983)*^b^*; USA | RCT | N = 50 (*n* = 13 males) (33.3 years), retention not reported  Adult outpatients; Clinical | IBs; II | 2 time-points: Pre- and post-intervention | Emotional (anxiety); Neuroticism | RET  *^e^* versus RET + Rational Role Reversal  *^d^* versus RET + REI  *^d^* versus Attention Alternative Treatment versus wait-list control | 12 x 45 min weekly session (12 weeks) | Face-to-face. Group or 1:1 format not reported | Significant decreases in IBs from pre to post for RET (*d* = -2.90), RET + Rational Role Reversal (*d* = -2.68) and RET + Rational Emotive Imagery (*d* = -2.75) as compared with Attention Alternative Treatment (*d* = -.54) and wait-list control group (*d* = -.19). RET + REI and RET + Rational Role Reversal had significantly further decreases in IBs from pre to post than RET alone. | Significant decreases in anxiety, depression and neuroticism from pre to post for the RET, RET + Rational Role Reversal, RET + Rational Emotive Imagery as compared with the other two groups. RET + REI and RET + Rational Role Reversal had significantly further decreases in all outcomes. |
| Mattick and Peters (1988); Australia | RCT | N = 51 (*n* = 24 males) (36.7 years), 86% retention  Phobics (social); Clinical | IBs; IBT (modified - only 39-items) | 3 time-points: Pre-, post-intervention and 3-month follow-up | Cognitive; Phobia; Self-perception (locus of control) | Guided Exposure + Cognitive Restructuring *^d^*  versus Guided Exposure | 6 x 2-hr weekly sessions (6 weeks) | Face-to-face group sessions | Significant decreases in IBs from pre to post for Guided Exposure Combined with Cognitive Restructuring (*d* = .46) and Guided Exposure (*d* = .42) which was maintained at follow-up (Guided Exposure Combined with Cognitive Restructuring *d* = .21; Guided Exposure *d* = .14). No significant differences between groups. | Significant improvements in end-state functioning, behavioural approach and self-rated avoidance from pre to post for Guided Exposure + Cognitive Restructuring as compared with Guided Exposure only. At follow-up, end state functioning and clinical improvement was significantly for Guided Exposure Combined with Cognitive Restructuring than Guided Exposure. |
| Mattick et al. (1989); Australia | RCT | N = 43 (*n* = 20 males) (41.75 years), 88% retention  Phobics (social); Clinical | IBs; IBT (modified - only 39-items) | 3 time-points: Pre-, post-intervention and 3-month follow-up | Cognitive; Phobia; Self perception (locus of control) | Guided Exposure versus Cognitive Restructuring *^d^* versus Guided Exposure with Cognitive Restructuring  *^d^* versus Wait-list control group | 6 x 2-hr weekly sessions (6 weeks) | Face-to-face group sessions | Non-significant pre-/post decreases observed in IBs for all groups except wait-list control group (Guided Exposure *d* = .29;  Cognitive Restructuring *d* = .79; Guided Exposure with Cognitive Restructuring *d* = 1.02; Wait-list control group *d* = .01). Between post and follow-up, greater decreases in IBs for Cognitive Restructuring group than other groups (Guided Exposure *d* = .28;  Cognitive Restructuring *d* = .77; Guided Exposure with Cognitive Restructuring *d* = -.08). | Significant improvements in phobic avoidance and severity and attitudinal variables for Guided Exposure, Cognitive Restructuring and Guided Exposure with Cognitive Restructuring compared to wait-list control group. At follow-up, greater improvements observed for Cognitive Restructuring compared to other groups. |
| Moon et al. (2021); Korea | RCT | N = 42 (*n* = 20 males) (32.61 years), 93% retention  Outpatients; Subclinical | RBs and IBs; SGABS | 3 time-points:  Pre-, post-intervention and 4-week follow-up | Emotional (depression); Psychophysiological | REBT (n = 21) versus Control group (n = 21) | 8 x 90 min weekly sessions (8 weeks) | Face-to-face group sessions | Significant decreases in IBs from pre to post for REBT (*d* = 1.57)  in comparison to control group (*d* = .01)  which was maintained at follow up for REBT group (REBT *d* = 1.51; Control *d* = -.09). | Significant decreases in depression scores and salivary cortisol levels from pre to post for REBT in comparison to control group which were maintained at follow up. |
| Munjack et al.  (1984); USA | RCT | N = 16 males (47.5 years), retention not reported  Males with erectile failure; Non-clinical | IBs; RBI | 2 time-points:  Pre- and post-intervention | Sexual behaviour | RET versus wait-list control group | 12 sessions, delivery of twice weekly (6 weeks) | Face-to-face. Group or 1:1 format not reported | No significant difference in IBs within or between groups. | RET significantly reduced sexual anxiety and significantly more attempts and successful intercourse. At follow-up, all successful intercourse had fallen from post-intervention but were significantly higher than pre-intervention success rates. |
| Rezaeisharif (2021); Iran | RCT | N = 80, retention not reported  Adults with substance misuse disorder; Clinical | IBs;  4-IBT-A | 2 time-points:  Pre- and post-intervention | Emotional (hopelessness) | Cognitive Restructuring  *^d^* (*n* = 40) versus No-contact control (*n* = 40) | 8 x 90 min weekly sessions (2 months) | Face-to-face group sessions | Significant decreases in IBs from pre- to post for Cognitive Restructuring in comparison to control group. | Significant decreases in hopelessness from pre- to post for Cognitive Restructuring in comparison to control group. |
| Szentagotai et al. (2008); Romania | RCT | N = 170 (*n* = 57 males) (37 years), 89% retention  Adult diagnosed with Major Depressive Disorder; Clinical | IBs: 1) Attitude and Belief Scale II and 2) Content analysis of thoughts while in a negative scenario | 4 time-points:  Pre-, mid- (7 weeks), post-intervention and 6-month follow-up | Cognitive; Emotional (depression) | REBT (*n* = 57) versus Cognitive Therapy *^e^*(*n* = 56) versus Pharmacotherapy (*n* = 57) | 20 sessions (14 weeks) & optional three booster follow-up sessions (14 weeks and booster sessions scheduled within the 6-month follow-up phase) | Face-to-face. Group or 1:1 format not reported | Significant decreases in IBs from pre to post for REBT (*d* = .46), Cognitive Therapy (*d* = .73), Pharmacotherapy (*d* = .94) which was only maintained for REBT (*d* = .00; Cognitive Therapy *d* = -.02; Pharmacotherapy *d* = -.13). | Significant decreases in automatic thoughts, core beliefs and depression from pre to post for all groups. No differences at follow-up. |
| Emmelkamp, Mersch and Vissia (1985); Netherlands | NRCT | N = 69, 88% retention  Socially anxious adults; Subclinical | IBs; IBT | 3 time-points: Pre-, post-intervention and 1-month follow-up | Emotional (anxiety); Phobia (social); Psychophysiological | RET versus Exposure in vivo versus Self-instructional Training | 6 x 2.5-hrs (duration not reported) | Face-to-face group sessions | Decreases in IBs from pre to post for all groups which was maintained at follow-up. No significant differences between the groups although RET was superior at reducing IBs. | Significant improvements in phobic anxiety, pulse phobic avoidance fear, social anxiety and symptom check list for RET group from pre to post. At follow-up, significant improvements in phobic anxiety and social anxiety for RET group. Significant decreases in anxiety pre to post for all conditions with no evidence that treatments were significantly superior to the other. |
| Grove et al. (2021); USA | NRCT | N = 94 (*n* = 83 males) (44.89 years), retention not reported  Post 9/11 military Veterans diagnosed with PTSD; Clinical | IBs; IBT | 2 time-points: Pre- and post-intervention | Emotional (anxiety & depression); Mental ill-health (PTSD) | REBT (*n* = 47) versus PTSD Recovery Programme (*n* = 47) | 5 x 90 min sessions (duration not reported) | Face-to-face group sessions | No significant decreases of IBs reported for REBT group (*d* = 0.35). | Significant decreases in depression and PTSD symptoms from pre to post for REBT group.  No significant findings for anxiety reported for REBT group. PTSD symptoms decreased significantly for both groups to a similar level. |
| Grove et al. (2023); USA | NRCT | N = 86 (n = 78 males) (44.60 years), retention not reported  Veterans diagnosed with PTSD; Clinical | IBs; IBS (1) | Pre - and post - intervention | Mental health; (PTSD, depression, anxiety) | REBT | 5 sessions | Not reported | Significant decreases in irrational beliefs from pre to post. | Significant decreases in irrational beliefs from pre to post (*d* = .37). |
| Hamberger and Lohr (1980); USA | NRCT | N = 1 male, 27 years, 100% retention  Student experiencing emotional distress due to divorce; Sub-clinical | IBs; IBT | 2 time-points: Pre- and post-intervention | N/A | Rational Restructuring (*n* = 1) | 13 x 50 min sessions (13 weeks) | Face-to-face 1:1 sessions | Significant decreases in IBs from pre to post. | N/A |
| Igna et al. (2014); Romania | NRCT | N = 85 males and females, 85% retention  Adults with chronic pain; Non-clinical | RBs and IBs; SGABS | 2 time-points: Pre- and post-intervention | Cognitive; Emotional (anxiety, depression, mood & wellbeing); Mindfulness; Physical health (pain) | CBT (*n* = 35) versus mindfulness-based CBT  *^d^* (*n* = 25) versus Pharmacological and Physiotherapy control group (*n* = 25). | 6 x 1-hr sessions, delivery twice weekly (3 weeks) | Face-to-face 1:1 sessions | Decreases in IBs from pre to post for all groups which were not significant (CBT *d* = .13; mindfulness-based CBT *d* = .60; Pharmacological and Physiotherapy control group (*d* = .05). | Significant changes in pain, anxiety and emotional wellbeing from pre to post for CBT. Significant changes in pain, anxiety, depression and distress from pre to post for Mindfulness-Based Cognitive Behaviour Group. Significantly lower levels of pain intensity for Mindfulness-Based Cognitive Behaviour Group compared to control group at post-test. No significant findings for mindfulness. |
| Jacobsen et al. (1987); USA | NRCT | N = 61 males (42.6 years), 69% retention  Psychiatric inpatients; Clinical | IBs; II | 2 time-points: Pre- and post-intervention | Mental ill-health (psychiatric & psychosomatic symptoms) | RET (*n* = 34) versus Usual Care control group (*n* = 27) | 9 sessions, delivery of three per week (3 weeks) | Face-to-face group sessions | Significant decreases in IBs for RET group in comparison to control group. | No significant findings in additional outcomes for both conditions. |
| Mersch et al. (1989; 1991)*^c^*; Netherlands | NRCT | N = 74 (*n* = 33 males) (32 years), 84% retention  Phobics (social); Clinical | IBs; RBI | 4 time-points: Pre-, post-intervention, 1.5-month follow-up and 14-month follow-up | Behavioural; Cognitive; Emotional (anxiety); Phobia; Social skills; Social validity | RET versus Social Skills Training | 8 x 2.5-hr weekly sessions (8 weeks) | Face-to-face group sessions | No significant pre-/post change observed in IBs for either group. No significant differences in level of improvement between both groups during follow-up. | No significant differences pre-/post change observed in any variable for either group. No significant differences in level of improvement between both groups during follow-up. Social validation findings not reported. |
| Neamtu and David (2016); Romania | NRCT | N = 30 (*n* = 9 males) (16.53 years), 100% retention  Children/ adolescents in residential care; Subclinical | IBs; CASI | 3 time-points: Pre-, post-intervention and 3-month follow-up | Behavioural social/emotional child problems; Emotional (anger, generic mood) | Rational Emotive and Cognitive Behavioural Therapy (*n* = 15) versus Wait-list control (*n* = 15) | 12 sessions (6 weeks) | Face-to-face group sessions | Significant decreases in IBs from pre to post for Rational Emotive and Cognitive Behavioural Therapy compared to wait-list control. | Significant decreases in externalizing and internalizing symptoms, negative dysfunctional emotions, and significantly more positive affect from pre to post for Rational Emotive and Cognitive Behavioural Therapy compared to waitlist control. Findings maintained at follow-up. |
| Nottingham and Neimeyer (1992); USA | NRCT | N = 449 (*n* = 169 males) (36.4 years), retention reported  In-patient psychiatric patients; Clinical | Irrational thinking; 1. IBS, 2. SPB | 2 time-points:  Pre and post-intervention (within 48 hours of the scheduled discharge date) | Cognitive; Emotional (anxiety & depression); Quality of life; Social skills; Social validity | RET (*n* = 372) versus 12-step Model of Treatment with no RET/CBT component (*n* = 77) | Inpatient programme of 19.5 hours per week (duration not reported) | Face-to-face group and 1:1 sessions | Significant decreases in IBs from pre to post for RET (IBS *d* = 1.71;  SPB *d* = -1.93)  when compared with 12-step model of treatment group (IBS *d* = 1.06;  SPB *d* = -1.55).  . | Significant improvements in cognitive outcomes, anxiety, depression, quality of life and social skills from pre to post for Rational Emotive Therapy when compared with 12-step model of treatment group. Participants reported that the RET component was the most helpful and enjoyable. |
| Ray et al. (1984); USA | NRCT | N = 62 males, retention not reported  Veteran alcoholic patients; Clinical | IBs; RBI | 2 time-points:  Pre- and post-intervention | N/A | RET within an alcohol treatment programme (*n* = 62) | Frequency not reported (6 weeks) | Face-to-face group and 1:1 sessions | Significant decreases in 6/11 IBs from pre- to post (*d* = -.88). | N/A |
| Riggs and Meyer (1981); USA | NRCT | N = 35 males, 54% retention  Adults with substance misuse disorder; Clinical | IBs; IBT | 2 time-points:  Pre- and post-intervention | N/A | Rational Behaviour Counselling (*n* = 35) | 5 x 1.5-hr weekly sessions (5 weeks) | Face-to-face group sessions | Significant decreases in IBs from pre- to post. | N/A |

*Note:* *^a^* N is the initial sample size for the intervention and control condition (if relevant). *^b^* Smith (1983) is a reanalysis of Lipsky et al. (1980). *^c^* Mersch et al. (1991) is a follow-up study to Mersch et al. (1989). *^d^* Intervention is based upon REBT principles. *^e^* Intervention is not based upon REBT principles.

General: CBT = Cognitive Behavioural Therapy; IBs = Irrational beliefs; NRCT = Non-randomised control trial; OCD = Obsessive Compulsive Disorder; PTSD = Post Traumatic Stress Disorder; RCT = Randomised control trial; REBT = Rational Emotive Behaviour Therapy; REI = Rational Emotive Imagery; RET = Rational Emotive Therapy.

Measures: 4-IBT-A = IBs Test-Ahvaz; ABS – 2 = Attitudes and Belief Scale 2; CASI = Child and Adolescent Scale of Irrationality; IBS (1); The Irrational Belief Scale; IBT = IBs Test; II = The Idea Inventory; RBI = Rational Behaviour Inventory; SGABS = General Attitudes and Beliefs Scale- Short Form; SPB = Survey of Personal Beliefs.

**Table 9**

*A Summary of Included Studies that Fall Within the Organisational Domain (n = 24 studies; 10 RCTs, 13 NRCTs and 1 MMS)*

| **Study; countr**y | **Design** | **Participants**  N*^a^*, sex (Mage), % retention; type | **Primary outcomes & measure(s)** | **Time-points** | **Additional outcomes** | **Intervention vs control/ comparison** | **Frequency (duration)** | **Mode of delivery** | **Results: Primary outcomes** | **Results: Additional outcomes** |
| --- | --- | --- | --- | --- | --- | --- | --- | --- | --- | --- |
| Ekwueme et al. (2023);  Nigeria | RCT | N = 128 (*n* = 45 males), retention not reported  Teachers; Non-clinical | IBs; TIBS | Pre, post and 3- month follow-up | Employee | REBT (*n* = 59) versus Waitlist control (*n* = 64) | 90 min sessions (3 months) | Face-to-face group sessions | Significant decreases in irrational beliefs for REBT in comparison to control which was maintained at follow-up. | Significant decreases in workplace deviant behaviours for REBT in comparison to control which was maintained at follow-up. |
| Ifelunni et al.(2022); Nigeria | RCT | N = 138 females, 89% retention  Teachers (primary); Non-clinical | IBs; Teacher Irrational Belief Scale | 3 time-points: Pre-, post-intervention and follow-up (no time frame provided) | Affect; Employee | REBT (*n* = 70) versus Waitlist control (*n* = 68) | 14 x 1hr weekly sessions (14 weeks) | Face-to-face group sessions | Decreases of IBs from pre to follow-up for REBT group. Significance not reported. | Significant improvements on wellbeing and multidimensional health states for REBT group from pre to post which was maintained at follow-up in comparison to controls. |
| Iremeka et al. (2021); Nigeria | RCT | N = 160 (*n* = 124 males), 100% retention  Construction workers; Non-clinical | IBs; WRIBQ | 3 time-points: Pre, post-intervention and 4-week follow-up | Emotional (stress) | REBT (*n* = 80) versus Control group (*n* = 80) | 8 x 75 min weekly sessions (8 weeks) | Face-to-face group sessions | Significant decreases in IBs for REBT group from pre to post (*d* = 13.70)  which was maintained at follow-up (*d* = .66). Significant decreases in IBs for REBT group in comparison to control group. No significant findings for control from pre to post (*d* = -.57) or at follow-up (*d* = .97). | Significant decreases in stress for REBT group from pre to post which was maintained at follow-up. Significant decreases in stress for REBT group in comparison to control group. |
| Möller and Botha (1996); South Africa | RCT | N = 44 males (40.86 years), 100% retention  Insurance workers; Non-clinical | IBs; TACQ | 3 time points: Pre-, post-intervention and 10-week follow-up | Emotional (hostility); Type A personality | REBT (*n* = 22) versus Control group (n=22) | 9 x 1.5 hr weekly sessions (9 weeks) | Face-to-face group sessions | Significant improvements in Type A IBs from pre to post for REBT in comparison to control group which were maintained at follow-up. | Significant improvements in Type A behaviour and hostility from pre to post for REBT in comparison to control group which were maintained at follow-up. |
| Ogbuanya et al. (2017); Nigeria | RCT | N = 108 (*n* = 92 males) (27.19 years), 100% retention  Workshop instructors; Non-clinical | IBs; WIOIBS | 3 time-points:  Pre-, post-intervention and 3-month follow-up | Emotional (stress) | REBC (*n* = 55) versus Wait-list control (*n* = 53) | 24 x 1 hr twice weekly sessions (12 weeks) | Face-to-face. Group or 1:1 format not reported | Significant decreases in IBs from pre to post for REBC (*d* = 2.46) group which was maintained at follow-up (*d* = .61). No significant findings for wait-list control from pre to post (*d* = -.15) to follow-up (*d* = -.31). | Significant decreases in occupational stress and significant improvements in work ability from pre to post for REBC which was maintained at follow-up. No significant findings for wait-list control. |
| Onuigbo et al (2018); Nigeria | RCT | N = 86 (*n* = 27 males) (39.38 years), retention not reported  Teachers; Non-clinical | IBs; TIBS | 3 time-points:  Pre-, post- intervention and 4-month follow-up | Emotional (stress) | REBT (*n* = 43) versus No-contact control (*n* = 43) | 24 x 90 min sessions, delivery of twice weekly (12 weeks) | Face-to-face. Group or 1:1 format not reported | Significant decreases in IBs from pre to post for REBT (*d* = 5.31)  which was maintained at follow-up (*d* = .36). Compared to waitlist control (pre to post *d* = .02; post to follow-up *d* = .35), REBT had significantly reduced IBs which was maintained at follow-up. | Significant decreases in stress from pre to post for REBT which was maintained at follow-up. Compared to waitlist control, REBT had significantly reduced stress which was maintained at follow-up. |
| Otu and Omeje (2021); Nigeria | RCT | N = 176 (*n* = 93 males) (28.21 years), 90% retention  University graduates; Non-clinical | IBs; DCBS | 3 time-points:  Pre-, post-intervention and 1-month follow-up | N/A | Rational Emotive Career Coaching  *^c^* (*n* = 88) versus Skill Acquisition and Career Development Programme (*n* = 88) | 16 x 50 min sessions, delivery twice weekly (8 weeks) | Face-to-face group sessions | Significant decreases in IBs from pre to post for Rational Emotive Career Coaching (*d* =10.67). which was maintained at follow-up (*d* = .27). Compared to Skill Acquisition and Career Development Programme (pre to post *d* = .07; post to follow-up *d* = .08)  , Rational Emotive Career Coaching had significantly reduced IBs which was maintained at follow-up. | N/A |
| Stanton (1989); Australia | RCT | N = 40, retention not reported  Teachers; Non-clinical | Irrational thinking: TII | 3 time-points:  Pre -, post-intervention and 12-month follow-up | Emotional (stress) | RET + Hypnosis *^c^* versus Stress Reduction Programme  *^c^* | 4 x 30-60 min weekly sessions (4 weeks) | Face-to-face. Group or 1:1 format not reported | Significant decreases in IBs from pre- to post for RET + Hypnosis (*d* = 2.63)  in comparison to Stress Reduction Programme (*d* = .96)  which was maintained at follow-up (RET + Hypnosis *d* = .52;  Stress Reduction Programme *d* = .46). | Significant decreases in stress from pre- to post for RET + Hypnosis and Stress Reduction Programme which was maintained at follow-up. RET + Hypnosis was significantly superior. |
| Thurman (1985a; 1985b)*^b^*; USA | RCT | N = 39 (*n* = 30 males) (46.6 years), 87% retention  University staff; Non-clinical | IBs: 1) RBI 2) TAIBI | 4 time-points:  Pre-, post-intervention, 6-month follow-up and 1-year follow-up | Emotional (anger and hostility); Psychophysiological; Type A personality; Social validity | Cognitive-behaviour Modification *^c^*  (*n* = 11) versus Cognitive-behaviour Modification + Assertion Training *^c^*  (*n* = 11) versus Minimal Treatment (*n* = 12) | 8 x 2-hr weekly sessions (8 weeks) | Face-to-face group sessions | Significant decreases in Type A IBs from pre to post for Cognitive-behaviour Modification (*d* = 1.78) and Cognitive-behaviour Modification + Assertiveness Training (*d* = 1.09) compared to Minimal Treatment (*d* = .21)  which was maintained at follow-up. No significant findings observed for IBs as measured by Rational Behaviour Inventory (Cognitive-behaviour Modification *d* = -.83; Cognitive-behaviour Modification + Assertiveness Training *d* = -.53; Minimal Treatment *d* = -.44). Cognitive-behaviour Modification and Cognitive-behaviour Modification + Assertiveness Training did not differ significantly from each other. | Significant decreases in Type A behaviour and hostility from pre to post for Cognitive-behaviour Modification and Cognitive-behaviour Modification + Assertiveness Training compared to Minimal Treatment. Significant decreases in anger from pre to post for Cognitive-behaviour Modification only. At both follow-ups, significant decreases in Type A behaviours, and speed and impatience behaviours for Cognitive-behaviour Modification + Assertiveness Training compared to Minimal Treatment. Cognitive-behaviour Modification + Assertiveness Training did not differ significantly from each other. Groups were statistically similar on therapist and treatment evaluation ratings. No significant findings were reported on physiological measures. |
| Ugwoke et al. (2017); Nigeria | RCT | N = 185 (*n* = 86 males) (38.14 years), 100% retention  Teachers; Non-clinical | IBs: TIBS | 3 time-points:  Pre-, post- intervention and 4-month follow-up | Emotional (stress) | Rational Emotive Health Education Intervention  *^c^*  (*n* = 93) versus Wait-list control group (*n* = 92) | 20 x 1-hr sessions (10 weeks) | Face-to-face group sessions | Significant decreases in IBs from pre- to post for Rational Emotive Health Education (*d* = 4.65) which was maintained at follow-up (*d* = 1.09). No significant findings for control group from pre to post (*d* = .10). | Significant decreases in stress from pre- to post for Rational Emotive Health Education. No significant findings for control group. Findings maintained at follow-up. |
| Bora et al. (2013); Romania | NRCT | N = 40 (*n* = 10 males) (35.33 years), 100% retention  Teachers; Non-clinical | IBs; TIBS | 3 time-points: Pre-, post-intervention and 4-month follow-up | Emotional; Dysfunctional behaviours; Self-perception (locus of control) | REE (*n* = 20) versus Control group (*n* = 20) | 15 weekly sessions (3 months) | Face-to-face group sessions | Significant decreases for IBs from pre to post (*d* = 3.27)  for REE which was not maintained at follow-up (*d* = -.26). No significant findings observed for control group (*d* = -.10) | Significant changes in locus of control and significant decreases in distress depression, dysfunctional emotions, sadness, worry and anxiety and dysfunctional behaviours from pre to post for REE which was not observed for control group. Generally, significant findings were not maintained at follow-up. |
| David and Matu (2013); Romania | NRCT | N = 22 (*n* = 15 males) (32.18 years), 82% retention  Managers; Non-clinical | RBs and IBs; M-RIBS | 2 time-points: Pre- and post-intervention | Coaching skills; Emotional (distress); Employee performance; Optimism and pessimism; Satisfaction with team | Rational Managerial Coaching Programming *^c^*  (*n* = 22) | 4 x 1-hr group coaching, 1 x 50-min individual coaching, 1 x 15-min shadowing session, 1 x 1.5-hr group managerial coaching session (6 months) | Face-to-face group and 1:1 sessions | Significant decreases in IBs (*d* = .39) and significant increases in RBs (*d* = -.84) from pre to post. | Significant decreases in dysfunctional negative emotions from pre to post when optimism level was controlled for. Significant increases in satisfaction with team from pre to post. |
| David et al. (2016); Italy | NRCT | N = 59 (*n* = 33 males) (35.9 years), retention not reported  Managers; Non-clinical | RBs and IBs; SGABS.  RBs and IBs; M-RIBS | 2 time-points: Pre- and post-intervention | Emotional (distress); Employee performance | Cognitive Behavioural Coaching  *^c^*  (*n* = 59) | One-off 5-hr session & one telephone coaching session (1 day with telephone session scheduled within 6 months) | Face-to-face group sessions. 1:1 sessions via telephone | Significant decreases in IBs  *(d* = 1.13) and significant increases in rational beliefs *(d* = .81) from pre to post. | Significant decreases in depressed mood and significant increases in managers' soft skills from pre- to post- test. No significant findings for distress, anxiety and employee performance. |
| De Jesus and Conboy (2001); Portugal | NRCT | N = 25 (*n* = 3 males) (41.7 years), retention not reported  Teachers; Non-clinical | IBs; IBS (3) | 2 time-points: Pre- and post-intervention | Emotional (stress, wellbeing); Motivation; Social validity | Relational-Training Stress-Management Course  *^c^*  (*n* = 25) | 10 x 3-hr sessions (duration not reported) | Face-to-face group sessions | Significant decreases in IBs from pre to post (*d* = .88) | Significant decreases in stress and significant increases in wellbeing from pre to post. No significant findings for motivation. Participants reported that the intervention was useful and should have been longer. |
| Ellis et al. (1989); USA | NRCT | No participant details provided, 63% retention  REBT therapists; Non-clinical | RBs; RM | 2 time-points: Pre-and post-intervention | None | RET | One-off 9-hr session (1 day) | Face-to-face group sessions | Significant increases in test rationality from pre to post. | N/A |
| Forman and Forman (1980); USA | NRCT | N = 17, 100% retention  School staff; Non-clinical | IBs; IBT | 2 time-points: Pre- and post-intervention | Social validity | RET (*n* = 17) | 10 x 1.5-hr sessions (duration not reported) | Face-to-face group and 1:1 sessions | Significant decreases in IBs from pre to post (*d* = 1.69). | Participants reported the course was applicable and practical. Some requested further time to practice techniques. |
| Kushnir and Malkinson (1993); Israel | NRCT | N = 40 males (48.1 years), 86% retention  Safety officers; Non-clinical | IBs; BI (modified - 30/100 items used) | 3 time-points: Pre-, post-intervention and 18-month follow-up | Cognitive; Physical health; Social skills; Social validity | RET (*n* = 22) versus Control group (*n* = 18) | No frequency reported (9 months) | Face-to-face group sessions | Significant decreases in IBs from pre to post for RET (*d* = .57) which was not maintained at follow-up (*d* = -.30). Significant decreases in IBs from pre to post for RET compared to control group (*d* = -0.09). | Significant decreases in somatic complaints and cognitive weariness from pre to post for RET which was maintained at follow-up. Significant increases in assertiveness from pre to post for RET. |
| Kushnir et al. (1994); Israel | NRCT | N = 48 (*n* = 12 males) (40.15 years), retention not reported  Occupational health staff; Non-clinical | IBs; BI (modified- 30/100 items used) | 3 time-points: Pre-, mid- and post-intervention | Psychosocial (ability); Social validity | RET (*n* = 23) versus Control group (*n* = 25) | 1st semester: 3-hour weekly session (one semester).  2nd semester: Group work & supervisor meeting every second week (no duration reported) | Face-to-face group and individual sessions | Significant decreases in IBs from pre to post for RET compared to control group. | Significant increases in psychosocial ability from pre to post for RET compared to control group. Participants reported improved self-awareness and ability to manage stress in professional and personal life. |
| Kushnir et al. (1998); Israel | NRCT | N = 64 (*n* = 16 males) (40.16 years), retention not reported  Occupational health staff; Non-clinical | IBs; BI (modified - 30/100 items used)  Low Frustration Tolerance; LFTS | 2 time-points: Pre- and post-intervention | Psychosocial (efficacy) | REBT (*n* = 39) versus control (*n* = 25) | 14 x 3-hr weekly sessions (14 weeks) | Face-to-face group sessions | Significant decreases in IBs and low frustration tolerance from pre to post for RET (IBs *d* = .46 compared to control group *d* = -.46). | Significant increases in psychosocial efficacy from pre to post for RET compared to control group. |
| Morris (1992); Canada | NRCT | N = 1 male (32 years), 100% retention  Warehouse employee; Non-clinical | IBs; IBT | 2 time-points:  Pre- and post-intervention | Emotional (anger, anxiety & depression); Mental ill-health; Physical health; Quality of life; Social validity | RADAR  *^c^* (*n* = 1) | 5 x 2-hr sessions (duration not reported) & 2 follow-up phone calls (2-week and 1-month) | Face-to-face 1:1 sessions | Significant decreases in IBs from pre to post. | Decreases in depression, anxiety and trait anger scores from pre to post. Participant reported improvements in mental health, physical health and quality of life. No statistical analysis reported. |
| Turner and Barker (2015); UK | NRCT | N = 11 (*n* = 2 males) (28-60 years), 91% retention  Blue-chip organisation workers; Non-clinical | RBs and IBs; SGABS | 4 time-points:  Pre-workshop 1, post-workshop 1, pre-workshop 2 and post-workshop 2 | Social validity | REBT (*n* = 11) | No frequency reported (1 month) | Face-to-face group sessions | Significant decreases in IBs (*d* = 1.62) from pre to post and increases in RBs (*d* = -.58). | Participants reported that they enjoyed the group work elements, reported changes towards a more rational philosophy and changes in a range of cognitions, emotions and behaviours. |
| Wood et al. (2021); UK | NRCT | N = 34 (*n* = 29 males) (42.39 years), retention not reported  Fire service personnel; Non-clinical | IBs; iPBI | 3 Phases: Baseline, post-intervention 3-month follow-up | Emotional (distress); Employee behaviours (presenteeism); Psychophysiological; Resilience; Social validity | REBC (*n* = 18) versus No-contact control (*n* = 18) | 4-6 x 25-60 min sessions, delivery of weekly/bi-weekly (12 weeks) | Face-to-face 1:1 sessions | Significant decreases in IBs from pre to post for Rational Emotive Behaviour Coaching (*d* = 1.48) compared to the control group (*d* = -.04)  which was maintained at follow-up for REBC (*d* = --.18; Control *d* = .09).  . Significant decreases in IBs for Rational Emotive Behaviour Coaching from baseline to post to follow-up. No statistically significant findings observed for control. | No significant findings for resilience, hair cortisol, psychological distress and presenteeism at any time point between Rational Emotive Behaviour Coaching and control group. Participants reported they were more capable of dealing with work and non-work adversities, had greater emotional control, empathy for others and perspective of negative experiences. |
| Woods (1987); USA | NRCT | N = 51 (*n* = 43 males) (41 years), 96% retention  Corporate workers; Non-clinical | IBs; IBT | 2 time-points:  Pre and post-intervention | Emotional (anxiety, depression & stress); Social skills; Type A personality | RET (*n* = 51) | 4 x 1.5-hr weekly sessions (4 weeks) | Face-to-face group sessions | Significant decreases in IBs from pre to post (*d* = .78). | Significant decreases in Type A behaviours, anxiety, anger and depression from pre to post. Significant increases in assertiveness from pre to post. |
| Jones et al. (2020); UK | MMS | N = 50 (*n* = 22 males) (45.86 years), 100% retention  Police personnel; Non-clinical | IBs; iPBI | 3 time-points: Pre-, post-intervention and 6-month follow-up | Motivation; Psychophysiological; Psychological needs; Social validity | REBC (*n* = 24) and Wait-list control group (*n* = 26) | 8 x 1-hr sessions (12 weeks) | Face-to-face 1:1 sessions | Significant decreases in IBs for Rational Emotive Behavioural Coaching from pre to post (*d* = 1.23)  which was maintained at follow-up (*d* = .50). No significant differences for Wait-list control group from pre to post (*d* = .80) or at follow-up (*d* = 1.17).  . | Significant increases in the satisfaction of basic psychological needs for Rational Emotive Behavioural Coaching from pre to post. No significant for Rational Emotive Behavioural Coaching on self-determined motivation and hair cortisol concentration. Social validation indicated that the intervention was satisfactory, accepted, meaningful and useful. There were mixed views on whether Rational Emotive Behavioural Coaching was beneficial to performance. |

*Note:* *^a^* N is the initial sample size for the intervention and control condition (if relevant). *^b^* Thurman (1985b) is a follow-up study to Thurman (1985a). *^c^* Intervention is based upon REBT principles.

General: IBs = Irrational beliefs; NRCT = Non-randomised control trial; MMS = Mixed methods study; RBs = Rational Beliefs; RCT = Randomised control trial; REBC = Rational Emotive Behaviour Coaching; REBT = Rational Emotive Behaviour Therapy; RET = Rational Emotive Therapy.

Measures: BI = Belief Inventory; DCBS = Dysfunctional Career Beliefs Scale; IBS (3) = Irrational Belief Scale; IBT = IBs Test; iPBI = The Irrational Performance Beliefs Inventory; LFTS = Low Frustration Tolerance Scale; M-RIBS = The Manager Rational and IBs Scale; RBI = Rational Behaviour Inventory; RM = Rationality Measure = SGABS = General Attitudes and Beliefs Scale- Short Form; TACQ = The Type A Cognitive Questionnaire; TAIBI = The Type A IBs Inventory; TIBS = Teacher Irrational Belief Scale; TII = Teacher Idea Inventory; WIOIBS = Workshop Instructors Occupational IBs Scale; WRIBQ = Work-related IBs Questionnaire.

**Table 10**

*A Summary of Included Studies that Fall Within the Relationships Domain (n = 12 studies; 7 RCTs and 5 NRCTs)*

| **Study; countr**y | **Design** | **Participants**  N*^a^*, sex (Mage), % retention; type | **Primary outcomes**  **& measure(s)** | **Time-points** | **Additional outcomes** | **Intervention vs control/ comparison** | **Frequency (duration)** | **Mode of delivery** | **Results: Primary outcomes** | **Results: Additional outcomes** |
| --- | --- | --- | --- | --- | --- | --- | --- | --- | --- | --- |
| Baucom and Lester (1986); USA | RCT | N = 48 (*n* = 24 males) (32.05 years), retention not reported  Romantic relationships; Non-clinical | IBs; 1) IBT, 2) ReBI | 3 time-points: Pre-, post-intervention and 6-month follow-up | Cognitive; Romantic relationships | CBT + Behaviour Marital Therapy *^b^* (*n* = 16) versus Behaviour Marital Therapy (*n* = 16) versus Wait-list control group (*n* = 16) | 12 x 1.5-hr weekly sessions (12 weeks) | Face-to-face 1:1 sessions for each couple | Significant decreases in IBs from pre to post for CBT + Behaviour Marital Therapy for females (IBT *d* = .83; ReBI *d* = 1.05) only but not for males (IBT *d* = .47; ReBI *d* = 1.30) which was not maintained at follow-up (IBT females *d* = -.17; IBT males *d* = .02; ReBI males *d* = -.50; ReBI females *d* = -.37). Significant decreases in IBs from pre to post for Behaviour Marital Therapy for females as measured by ReBI only (IBT *d* = .09; ReBI *d* = 1.25) but not for males (IBT *d* = -.16; ReBI *d* = .60) which was not maintained at follow-up (IBT females *d* = -.10; IBT males *d* = .33; ReBI males *d* = -.03; ReBI females *d* = -.28). No significant differences for wait-list control group or significant differences between CBT + Behaviour Marital Therapy and Behaviour Marital Therapy. | Significant improvements in cognitive measures and marital adjustment for  CBT + Behaviour Marital Therapy and Behaviour Martial Therapy from pre to post. No significant pre-/post change between Behaviour Marital Therapy and CBT + Behaviour Marital Therapy groups on any variable. Generally, findings maintained at follow-up. |
| David (2014); Romania | RCT | N = 130 (*n* = 14 males), 82% retention  Parents; Non-clinical | RBs and IBs; SGABS.  RBs and IBs; P-RIBS | 3 time-points: Pre-, post-intervention and 1-month follow-up | Behavioural/emotional problems in children (teacher rated); Emotional (depression, distress); Parenting (dysfunctional discipline style and competence) | Rational Parenting Programme *^b^*  (*n* = 45) versus Standard Cognitive Behavioural Parenting Programme (*n* = 47) versus Wait-list control group (*n* = 38) | 10 x 1.5-hr weekly sessions (10 weeks) | Face-to-face group sessions | Significant decreases in IBs from pre- to post-intervention for Rational Parenting Programme compared to wait-list control group. | Significant decreases in child disruptive behaviour, parent depressive symptoms, dysfunctional parenting and parental distress as well as a significant increases in parent competence from pre- to post-intervention for Rational Parenting Programme compared to wait-list control group. |
| David et al. (2017); Romania | RCT | N = 53 (*n* = 5 males) (35.97 years), 100% retention  Parents; Non-clinical | RBs and IBs; P-RIBS | 2 time-points: Pre- and post-intervention | Behavioural/emotional problems in children; Emotional (parental stress); Parenting (behaviours, competence, parent – child interaction) | Rational Positive Parenting Program  *^b^* (*n* = 25) versus Rational Positive Parenting Program + Attention Bias Modification  *^b^* (*n* = 28) | One initial face-to-face meeting followed by eight computer modules with two modules released per week (duration not reported) | Computer-based 1:1 sessions (accessed at own convenience) | Significant decreases in irrational thinking for Rational Positive Parenting Program (*d* = .29)  and Rational Positive Parenting Program + Attention Bias Modification (*d* = .26)  from pre to post. | Significant decreases in parental distress from pre to post intervention for Rational Positive Parenting Program group and only marginal decreases for the Rational Positive Parenting Program + Attention Bias Modification. Significant increases in parents’ satisfaction, self-esteem and self-efficacy from pre to post in both groups. No significant differences for positive or negative interactions. |
| Ede & Okeke (2022); Nigeria | RCT | N = 67 (*n* = 32 males) (age information not provided), 100% retention  Couples undergoing divorce; Non-clinical | RBs & IBs; P-RIBS | 3 timepoints;  Pre-, post-intervention and follow-up (3-month) | Affect (depression); Romantic relationships (marital burnout) | Rational-Emotive Couple Intervention (*n* = 67) | 14 x 2hr sessions (14 weeks) | Face-to-face | Change and significance in irrational/rational beliefs not reported on. | Parental irrational and rational beliefs are positively and significantly associated with marital burnout among couples seeking divorce. Depression is positively and significantly associated with marital burnout among couples seeking divorce. Change and significance in depression not reported on. Significant decreases in marital burnout from pre to post which was maintained at follow-up. |
| Gavita and Calin (2013); Romania | RCT | N = 32 (*n* = 21 males) (8.9 years children, 37.25 years parents), 100% retention  Parents and their children; Subclinical children and non-clinical parents (classed as subclinical overall due to intervention focus) | IBs; CASI.  RBs and IBs; P-RIBS. | 2 time-points: Pre- and post-intervention | Behavioural/emotional problems in children; Emotional (child anger, parent distress); Parenting (practices) | RETMAN Rational Stories  *^b^* ( *n* = 15 children) versus the Short Rational Parenting Programme  *^b^* (*n* = 17 children) | RETMAN Rational Stories: 9 x 40 min sessions delivered three times per week (3 weeks).  Short Rational Parenting Programme: 3 x 2-hr weekly sessions (3 weeks). | Face-to-face group sessions | Significantly greater decreases in irrational demand for fairness in children and IBs in parents for RETMAN Rational Stories compared to Short Rational Parenting Programme at post-test. | Significantly lower internalizing/externalizing problems and anger in children in RETMAN Rational Stories condition compared to Short Rational Parenting Programme at post-test. Significant improvements in dysfunctional parenting (laxness and verbosity) in Short Rational Parenting Programme compared to RETMAN Rational Stories. |
| Huber and Milstein (1985); USA | RCT | N = 34 (21-51 years), 100% retention  Romantic relationships; Non-clinical | IBs; IBT | 2 time-points: Pre- and post-intervention | Romantic relationships (relationship beliefs, marital satisfaction); Social validity; Therapy expectation | Cognitive Restructuring  *^b^* (*n* = 18) versus Wait-list control group (*n* = 16) | 6 x 45 min weekly sessions (6 weeks) | Face-to-face 1:1 sessions | No significant findings for IBs from pre to post for Cognitive Restructuring (demand for approval *d* = -.37)  or wait-list control group (*d* = .06). | Significant decreases  of unrealistic relationship beliefs for Cognitive Restructuring. Significant increases in marital satisfaction and therapy expectation for Cognitive Restructuring. No change for wait-list control group. |
| Joyce (1995); Australia | RCT | N = 48 (*n* = 13 males), retention not reported  Parents; Non-clinical | IBs; PIBS | 3 time-points: Pre-, post intervention and 10-month follow-up | Behavioural social/emotional child problems  ; Emotional; Parenting; Romantic relationships; Social validity | Rational Emotive Parenting Programme  *^b^* versus Wait-list control group | 9 x 1.5-hr weekly sessions (9 weeks) | Face-to-face group sessions | Significant reductions in IBs for Rational Emotive Parenting Programme group (*d* = -1.47) in comparison to control group which was maintained at follow-up (*d* = 0.00). | Significant decreases in guilt for Rational Emotive Parenting Programme group in comparison to control group. No significant differences in child domain or spouse measures for both groups. Social validation findings not reported. Findings maintained at follow-up. |
| Cramer (2005); UK | NRCT | N = 174 (*n* = 47 males) (19.69 years), 100% retention  Romantic relationships; Non-clinical | IBs; ReBI (modified – only Disagreement Is Destructive sub-scale used) | 2 time-points: Pre- and post-intervention | Romantic relationships | No Awfulizing *^b^* versus (*n* = 29) No Musts *^b^* (*n* = 29) versus No Damning *^b^* (*n* = 29) versus No Over-generalising *^b^* (n = 29) versus All Rational beliefs *^b^* (*n* = 29) versus Control group (*n* = 29) | One-off session (1 day) | Face-to-face. Group or 1:1 format not reported | Significant decreases in the irrational tendency to agree that disagreements are destructive for the All Rational Beliefs (*d* = .62)  and No Musts conditions. No significant findings for control group  (*d* = -.27). | Significant increases in relationship dissatisfaction for All Rational Beliefs and No Damning conditions. |
| McNaughton-Cassil et al. (2002); USA | NRCT | N = 80 (*n* = 35 males) (34.45 years), retention not reported  Couples undergoing IVF; Non-clinical | IBs; IBS (4) | 2 time-points: Pre- and post-intervention | Emotional (anxiety & depression); Optimism/pessimism; Social support | Brief Couples Support *^b^* (*n* = 43) versus Control group (*n* = 37) | 6 x 1.5 hr twice weekly sessions (≈3 weeks/IVF treatment length) | Face-to-face group sessions | Significant increases in IBs from pre to post for males in Brief Couples Support (*d* = -.17). No significant differences observed for females in Brief Couples Support (*d* = -.02) or for control group (males *d* = -.34; females *d* = .09). | Significant decreases in anxiety and depression from pre to post for females in Brief Couples Support. No significant differences for females in optimism or social support. Significant increases in optimism from pre to post for males in Brief Couples Support. Significant increases in optimism for Brief Couples Support in comparison to control group. |
| Mueller and Moskowitz (2020); USA | NRCT | N = 3 females (35.33 years), 100% retention  Mothers of children diagnosed with ASD; Non-clinical | RBs and IBs; P-RIBS | Baseline Phase: once per week prior to the start of the intervention.  Intervention phase: once per week at the start of each session (8 sessions) | Cognitive; Emotional (stress); Dysfunctional behaviours; Parenting | Positive Family Intervention (*n* = 3) | 8 x 90 min weekly sessions (8 weeks) | Face-to-face 1:1 sessions | Two out of three mothers reported significant decreases in IBs from pre to post (*d* = 1.67 - *d* = 2.07). | Significant decreases in parent rating of problem behaviour and observed child problem behaviour from pre to post. |
| Trip et al. (2019); Romania | NRCT | N = 85 females (27 years) 82% retention  Mothers; Non-clinical | RBs and IBs; SGABS | 2 time-points: Pre- and post-intervention | Emotional (distress); Dysfunctional behaviours | Rational Emotive Behavioural Parent Education (*n* = 25) versus Control group (*n* = 45) | 10 x 30 min weekly sessions (10 weeks) | Face-to-face group sessions | Significant decreases in IBs from pre to post for Rational Emotive Behavioural Parent Education (*d* = 2.75) in comparison to control group (*d* = -.01). Significant increases in RBs from pre to post for Rational Emotive Behavioural Parent Education (*d* = -.55) in comparison to control group (*d* = .02) | Significant decreases in depression, dysfunctional emotions and distress from pre to post for Rational Emotive Behavioural Parent Education in comparison to control group. |
| Yu & Schill (1976); USA | NRCT | N = 23 males and females (18 – 39 years), retention not reported  General population who considered themselves to be sensitive to criticism from others; Non-clinical | IBs; ISTM | 3 time-points: Pre-, post-intervention and follow-up | Social skills | RET (*n* = 8) versus Attention Placebo (*n* = 8) versus No-Treatment Placebo (*n* = 7) | 6 sessions (duration not reported) | Face-to-face 1:1 sessions | Increases in rationality from pre to post for all groups but greatest for RET which was also greatest at follow-up. Significant findings not reported. | Decreases in threat from pre to post for all groups although greatest change for RET group. Decreases in fear ratings from pre to post for attention placebo and RET groups although greatest change for RET group. Significant findings not reported. |

*Note:* *^a^* N is the initial sample size for the intervention and control condition (if relevant). *^b^* Intervention is based upon REBT principles.

General: ASD = Autism Spectrum Disorder; CBT = Cognitive Behavioural Therapy; IBs = Irrational beliefs; IVF = In vitro fertilisation; NRCT = Non-randomised control trial; RCT = Randomised control trial.

Measures: CASI = Child and Adolescent Scale of Irrationality; IBS (4) = Irrational Belief Scale (4); IBT = IBs Test; ISTM; Irrational Self-Talk Measure; P-RIBS = Parental Rational and IBs Scale; PIBS = Parent-IBs Scale; ReBI = Relationship Belief Inventory; SGABS = General Attitudes and Beliefs Scale- Short Form.

**Table 11**

*A Summary of Included Studies that Fall Within the Self-identified Healthcare Need Domain (n = 17 studies; 11 RCTs and 6 NRCTs)*

| **Study; countr**y | **Design** | **Participants**  N*^a^*, sex (Mage), % retention; type | **Primary outcomes & measure(s)** | **Time-points** | **Additional outcomes** | **Intervention vs control/ comparison** | **Frequency (duration)** | **Mode of delivery** | **Results: Primary outcomes** | **Results: Additional outcomes** |
| --- | --- | --- | --- | --- | --- | --- | --- | --- | --- | --- |
| Artiran and DiGiuseppe (2021); Turkey | RCT | N = 36 (*n* = 17 males) (26.72 years), 86% retention  Outpatients; Sub-clinical | RBs and IBs; SGABS | 3 time-points: Pre-, post-intervention and 3-month follow-up | Emotional (anxiety and depression, healthy/unhealthy negative emotions wellbeing) | REBT (*n* = 18) versus Humanistic Client-Centred Therapy (*n* = 18) | 12 x 50 min weekly sessions (12 weeks) | Face-to-face group sessions | Significant decreases for IBs from pre to post for REBT group (*d* = .68) which was maintained at follow-up *(d* = .28). No significant pre-/post change observed in IBs for REBT group when compared to Humanistic Client-Centred Therapy group (*d* = .06). | Significant decreases for unhealthy and healthy negative emotions and ontological wellbeing from pre to post for REBT group. Significant reductions in depressive and anxiety symptoms observed for REBT group when compared to Humanistic Client-Centred Therapy group which was maintained at follow-up. |
| Biran and Wilson (1981); USA | RCT | N = 22 (*n* = 7 males) (39.86 years), 100% retention  Phobics; Clinical | IBs; IBT | 4 time-points: Pre-, post-intervention, 1-month follow-up and 6-month follow-up | Behavioural; Cognitive; Emotional (fear, depression); Social skills; Psychophysiological | Cognitive Restructuring *^b^* versus Guided Exposure | 5 x 50 min sessions (2-3 weeks) | Face-to-face 1:1 sessions | Significant decreases for IBs from pre to post for Cognitive Restructuring group only which was maintained at follow-up. No significant findings for Guided Exposure. | Significant decreases in fear and depression from pre to post for Cognitive Restructuring group. Significant improvements in approach behaviour and self-efficacy as well as significant decreases in fear from pre to post for both groups. Significantly greater decreases in skin potential and heart rate for Guided Exposure than Cognitive Restructuring pre to post. Generally, findings maintained at follow-up. |
| Eseadi et al. (2016); Nigeria | RCT | N = 26 (*n* = 13 males) (36.44 years), 100% retention  Children who had experienced adverse childhood stress; Subclinical | RBs and IBs; REBTQ | 2 time-points: Pre- and post-intervention | Emotional (adverse childhood stress) | Cognitive Restructuring Intervention Programme  *^b^* (*n* = 13) versus Control group (*n* = 13) | Frequency not reported (12-weeks with follow-up meetings scheduled 9-months post-intervention) | Face-to-face group sessions | Significant decreases in IBs from pre to post for Cognitive Restructuring Intervention Programme (*d* = 7.22). No significant findings for control group (*d* = -.96). | Not reported. |
| Hovland (1995); Norway | RCT | N = 36 females (37.8 years), retention not reported  Adults with severe anxiety; Subclinical | Irrational Standards or Values; SDVOS | 3 time-points: Pre- and post-intervention and 5-month follow-up | Emotional (anxiety); Self-perception (self-awareness); Social skills | RET Assertiveness Training *^b^* (*n* = 26) versus Self-help (*n* = 10) | 6 x 3-hr weekly sessions (6 weeks) | Face-to-face group sessions | Significant decreases in IBs from pre to post for RET Assertiveness Training (*d* = .83) which was greater compared to control group. No significant findings reported at follow-up (*d* = -.11). Raw data not presented for self-help group. | Significant decreases in perceived non-assertiveness from pre to post for RET Assertiveness Training which was greater compared to control group. No significant findings reported at follow-up. |
| Johnson and Ridley (1992); USA | RCT | N = 10 (*n* = 6 males) (34.2 years), retention not reported  Students seeking counselling for depression; Sub-clinical | IBs; EIVS | 2 time-points: Pre- and post-intervention | Cognitive; Emotional (depression) | REBT  *^b^* (*n* = 5) versus Christian REBT  *^b^* (*n* = 5) | 6 x 50 min twice weekly sessions (3 weeks) | Face-to-face. Group or 1:1 format not reported | Significant decreases in IBs for Christian REBT group (*d* = 1.12) only from pre to post. Decreases in REBT from pre to post (*d* = 1.44). | Significant improvements in depression and automatic thoughts for REBT and Christian REBT group from pre to post. |
| Kanter and Goldfried (1979); USA | RCT | N = 68 (*n* = 18 males) (35.6 years), 91% retention  Adults with anxiety; Sub-clinical | IBs; IBT | 3 time-points: Pre-, post-intervention and 9-week follow-up | Emotional (anxiety); Cognitive; Social skills; Psychophysiological | Systematic Rational Restructuring  *^b^* (*n* = 17) versus Desensitization (*n* = 16) versus Desensitization + Rational Restructuring  *^b^* (*n* = 19) versus Wait-list control (*n* = 16) | 7 x 1.5-hr weekly sessions (7 weeks) | Face-to-face group sessions | Significant decreases in IBs for Systematic Rational Restructuring (*d* = 1.05)  and Desensitization + Rational Restructuring (*d* = .94)  from pre to post compared to waitlist control (*d* = -.04)  and Desensitization  (*d* = .05)  which was maintained at follow-up (Systematic Rational Restructuring *d* = .18; Desensitization + Rational Restructuring *d* = .15). | Significant decreases in anxiety for Systematic Rational Restructuring and Desensitization + Rational Restructuring from pre to post compared to waitlist control and Desensitization which was maintained at follow-up. No significant findings for pulse. |
| Lake et al. (1979); USA | RCT | N = 24 (*n* = 5 males) (33 years), retention not reported  Adults with severe headaches; Non-clinical | IBs; IBT | 3 time-points: Pre-, post-intervention and 3-month follow-up | Physical health; Psychophysiological; Social validity | RET + Digit Temperature Biofeedback *^b^* (*n* = 6) versus Digit Temperature Biofeedback (*n* = 6) versus EMG Biofeedback (*n* = 6) versus Wait-list control (*n* = 6) | 8-10 x 30 - 70 min sessions, delivery of twice a week (4-5 weeks) | Face-to-face 1:1 sessions | Significant decreases in IBs for all conditions over time but no significant differences between groups. | No significant pre-/post change observed in pain for RET + Digit Finger Biofeedback. Participants reported that RET was the most helpful aspect of the intervention. Clinical headache improvement observed for biofeedback groups in comparison to control from pre to post. |
| Mersch (1995); Netherlands | RCT | N = 34 (*n* = 23 males) (35.6 years), 88% retention  Phobics (social); Clinical | IBs; IBI | 4 time-points: Pre -, post-intervention, 3-month follow-up and 1.5-year follow-up | Cognitive; Emotional (anxiety) | Exposure in Vivo versus Integrated Treatment *^b^* versus Wait-list control | 14 x 1-1.5 hr weekly sessions (14 weeks) | Face-to-face 1:1 sessions | Decreases in IBs for Integrated Treatment and Exposure in Vivo (not clear whether this was significant). No significant differences between the three groups in IBs from pre to post to follow-up. | Significant improvements in anxiety for both treatment groups in comparison to wait-list control which was maintained at follow-up. No treatment group was superior. |
| Omeje et al. (2018);  Nigeria | RCT | N = 124 (n = 85 males) (33.76 years), 100% retention  HIV positive outpatients with alcohol use disorder; Clinical | IBs related to alcohol; ARIBS | 3 time-points:  Pre-, post-intervention and 2-week follow-up | Alcohol consumption | Rational Emotive Health Therapy  *^b^* (*n* = 61) versus Wait-list control group (*n* = 63) | 20 x 50 min sessions delivery of twice weekly (10 weeks) | Face-to-face group sessions | Significant decreases in alcohol related IBs from pre to post for Rational Emotive Health Therapy (*d* = 5.04)  which was maintained at follow-up (*d* = .55). Compared to waitlist control (pre to post *d* = .24; post to follow-up (*d* = .19) Rational Emotive Health Therapy had significantly reduced IBs which was maintained at follow-up. | Significant decreases in alcohol consumption from pre to post for Rational Emotive Health Therapy which was  maintained at follow-up. Compared to wait-list control, Rational Emotive Health Therapy group had significantly reduced alcohol consumption which was maintained at follow-up. |
| Syzmanski and O'Donohue (1995) USA | RCT | N = 32 (*n* = 9 males) (18.4 years), specific details not provided but 'high levels of attrition’ reported  Phobics (spider); Clinical | IBs: IB-S | 2 time-points:  Pre- and post- intervention | Behavioural; Emotional (anxiety); Phobia | Cognitive Restructuring  *^b^* versus Cognitive Restructuring with Spider  *^b^* versus Cognitive Restructuring with Snake  *^b^* versus In Vivo Exposure with Spider versus Spider Facts Lecture versus No treatment control | 3 x 1-hr sessions (2 weeks) | Face-to-face group sessions | Significant decreases in IBs related to spiders from pre to post for Cognitive Restructuring (*d* = 3.53), Cognitive Restructuring with Spider (*d* = 1.67) and Exposure (*d* = 1.84) in comparison to no treatment control (*d* = .12), Spider Facts Lecture (*d* = .50). | Significant decreases in spider phobia from pre to post for Cognitive Restructuring and Cognitive Restructuring with Spider in comparison to no treatment control. Significant improvements in behavioural approach test from pre to post for the Cognitive Restructuring in comparison to no treatment control. |
| Warren et al. (1988); USA | RCT | N = 33 ([*n* = 14 males) (37.8 years), 92% retention  Adults with low self-esteem; Non-clinical | IBs; RBI | 3 time-points: Pre-intervention, post-intervention and 6-month follow-up | Cognitive; Emotional (anger, anxiety & depression); Self-perception (self-esteem) | RET versus CBT  *^c^* versus Wait-list control | 8 x 1.5-hr weekly sessions (8 weeks) | Face-to-face group sessions | Significant decreases in IBs from pre- to post-intervention for CBT (*d* = -1.33)  and RET (*d* = -1.42). Significant findings were not maintained at follow-up (RET *d* = -.54; CBT *d* = -.25). No significant findings between-groups or for control group from pre to post (*d* = .32). | Significant improvements in self-esteem, depression, anxiety, distress, social avoidance and cognitive outcomes from pre- to post-intervention for CBT and RET. Significant findings were not maintained at follow-up. No significant findings between-groups or for control group. |
| Adekoya et al. (2023); Nigeria | NRCT | N = 42 (*n* = 22 males), retention not reported  Parents; Non-clinical | Irrational beliefs; Irrational eleifs about Sex Education (has been validated) | 2 time-points: Pre- and post-intervention | None | Rational Emotive Health Education Programme | Not reported | Not reported | Significant decreases in irrational beliefs from pre to post | N/A |
| Keller et al. (1975); USA | NRCT | N = 30 (*n* = 2 males) (68 years), retention not reported  Older adults; Non-clinical | IBs; AIII | 2 time-points: Pre- and post-intervention | Emotional (anxiety) | RET (*n* = 15) versus Waitlist control (*n* = 15) | 4 x 2-hr weekly sessions (four weeks) | Face-to-face group sessions | Significant decreases in IBs for RET group in comparison to waitlist control group. | Significant decreases in anxiety for RET group in comparison to waitlist control group. |
| Kirkby (1994); Australia | NRCT | N = 48 females (28.9 years), 88% retention  Females with severe premenstrual symptoms; Non-clinical | RBs and IBs; GABS | 3 time-points: Pre-, post-intervention and 9-month follow up | Emotional (anxiety); Physical health; Social validity | Coping Skills Training + RET (*n* = 16) versus Awareness Through Movement (*n* = 16) versus Wait-list control group (*n* = 16) | 6 x 1-hr weekly sessions (6 weeks) | Face-to-face group sessions | Significant decreases in IBs from pre to post for Coping Skills Training + RET (*d* = 1.70)  in comparison to Awareness through Movement group (*d* = 1.06) and wait-list control group (*d* = .53). At follow-up, decreases were only maintained for Awareness through Movement group (*d* = .03; Coping Skills Training + RET  *d* = -.03; Wait-list control group *d* = -.08). | Significant improvements in premenstrual symptoms from pre to post for Coping Skills Training + RET in comparison to Awareness through Movement group and wait-list control group which was maintained at follow-up. No significant findings observed for anxiety. Participants reported that the intervention reduced their symptoms and improved quality of life. |
| McKnight et al. (1984); USA | NRCT . | N = 9 females (35.7 years), retention not reported  Adults with major affective disorder; Clinical | IBs; PBI (2) | 11 time-points: Pre- and post-intervention and 1-year follow-up. Also, weekly measures. | Emotional (depression); Mental ill-health; Social skills | Social skills training + RET (*n* = 9) | 8 sessions (8 weeks) | Face-to-face group sessions | Significant decreases in IBs from pre to post. | Significant decreases in depression and significant increases in social skills from pre to post. Findings maintained at follow-up. |
| Newhouse and Schwager (1978); USA | NRCT | N = 30 (27 years), retention not reported  Disadvantaged adults; Non-clinical | Irrational Personality Traits; IPTI  (modified – only two subscales used) | 2 time-points: Pre- and post-intervention | Self-perception (self-concept) | RBT versus Eclectic Cognitive | 10 sessions (duration not reported) | Face-to-face group sessions | Significant decreases in IBs from pre to post for RBT only. | Significant improvements in inner directedness, self-acceptance from pre to post for RBT in comparison to Eclectic Cognitive. |
| Pasarelu et al (2021); Romania | NRCT | N = 15 (*n* = 5 males) (13.53 years), 100% retention  Adolescents diagnosed with a mental health illness; Clinical | IBs; CASI | 2 time-points:  Pre- and post-intervention | Cognitive; Emotional (anxiety, depression); REBT knowledge; Social validity | REBT via a computer (*n* = 15) | 9 modules (6 weeks) | Computer-based 1:1 sessions (accessed at own convenience) | Significant decreases in IBs from pre- to post (*d* = .91). | Significant decreases in anxiety and depression from pre- to post. Significant increases in REBT knowledge from pre- to post. Participants reported high satisfaction and user-friendliness. |

*Note: ^a^* N is the initial sample size for the intervention and control condition (if relevant). *^b^* Intervention is based upon REBT principles. *^c^* Intervention did not include REBT principles.

General: CBT = Cognitive Behavioural Therapy; EMG = Electromyography; IBs = Irrational beliefs; NRCT = Non-randomised control trial; RBs = Rational Beliefs; RBT = Rational Behaviour Therapy; RCT = Randomised control trial; REBT = Rational Emotive Behaviour Therapy; RET = Rational Emotive Therapy.

Measures: AIII = Adult Irrational Ideas Inventory; ARIBS = Alcohol-related IBs Scale; CASI = Child and Adolescent Scale of Irrationality; EIVS = Ellis Irrational Values Scale; GABS = General Attitudes and Beliefs Scale; IB-S = IBs Concerning Spiders; IBI = Irrational Beliefs Inventory; IBT = IBs Test; IPTI = Irrational Personality Trait Inventory; PBI (2) = Personal Beliefs Inventory; RBI = Rational Behaviour Inventory; REBTQ = Rational-Emotive Behaviour Therapy Questionnaire; SDVOS = Self-defeating Value-Orientation Scale; SGABS = General Attitudes and Beliefs Scale- Short Form.

**Table 12**

*A Summary of Included Studies that Fall Within the Sport and Exercise Domain (n = 24 studies; 4 RCTs, 18 NRCTs and 2 MMS)*

| **Study; countr**y | **Design** | **Participants**  N*^a^*, sex (Mage), % retention; type | **Primary outcomes& measure(s)** | **Time-points** | **Additional outcomes** | **Intervention vs control/ comparison** | **Frequency (duration)** | **Mode of delivery** | | **Results: Primary outcomes** | | **Results: Additional outcomes** |
| --- | --- | --- | --- | --- | --- | --- | --- | --- | --- | --- | --- | --- |
| Nejati et al. (2022); Iran | RCT | N = 24 males (15.9 years), retention not reported  Athletes; Non-clinical | IBs; iPBI | 3 timepoints; Pre-, post-intervention and 4-month follow-up | Affect (social anxiety); Performance (sport) | REBT (*n* = 12) versus Placebo based on soccer technology videos (*n* = 12) | 10 sessions | Face-to-face group sessions | | Significant decreases in IBs from pre to post for REBT group (*d* = 10.57)  which was maintained at follow up (*d* = .61). Significant difference between REBT and control in IBs with no change observed for control (pre to post; *d* = .79; post to follow-up *d* = -.92). | | Significant decreases in social anxiety from pre to post which was maintained at follow up for REBT group. Significant difference between REBT and control in social anxiety with no change observed for control. Significant improvements in performance under pressure from pre to post which was maintained at follow up for REBT group. Significant difference between REBT and control in performance under pressure with no change observed for control. |
| Turner, Slater and Barker (2014a); UK | RCT | N = 17 males (16.71 years), 100% retention  Athletes; Non-clinical | RBs and IBs; SGABS*^b^* | 8 time-points: 1) Pre-, 2) - 6) five times immediately after the intervention had commenced, 7) 1-month follow-up, 8) 2.5- month follow-up. | Social validity | REBT (*n* = 9) versus Emotion Control Program (*n* = 8) | 3 x 40 min weekly sessions (3 weeks) | Face-to-face group sessions | | Significant decreases in IBs from pre- to post for REBT in comparison to Emotion Control Programme. At follow-up, only ‘need for achievement’ and ‘demand for fairness’ remained reduced from pre-test levels. | | Participants reported that the intervention would help them modify their thoughts, emotions and behaviours and help performance. |
| Vertopoulos and Turner (2017); Greece | RCT | N = 20 males (16.35 years), 100% retention  Athletes; Non-clinical | IBs; 1) SGABS, 2) Qualitative inspection of participant’s script | 4 phases:  Pre-intervention (four time points across 4 weeks), REBT education stage (four weeks), REPDMS versus no REPDMS (one week) and post-intervention (3 weeks) | N/A | REBT  *^c^* (*n* = 11) versus REBT + REPDMS  *^c^* (*n* = 9) | REBT: 4 x 40 min sessions (4 weeks).  REBT + REPDMS : 5 x 40 min sessions (5 weeks). | Face-to-face group sessions | | Statistical and visual analyses demonstrated significant decreases in IBs and significant increases in rational beliefs from pre- to post-REBT education for both groups. Further benefits noted for the REBT + REPDMS. Qualitative inspection of REPDMS script revealed that demandingness was the main core belief. | | N/A |
| Wood, Barker, Turner & Thomson (2018); UK | RCT | N = 10 males (28.36 years), 100% retention  Athletes; Non-clinical | RBs and IBs; SGABS | 4 time-points: Pre-intervention, time-point 1, time-point 2, post-intervention. Each time-point represents a monthly training camp. | Emotional (anxiety); Psychophysiological; Sport performance; Social validity | REBT (*n* = 10) | 1 x 1-hour session (1 day) | | Face-to-face group sessions | | Descriptive statistics and effect sizes used to analyse data. Immediate and small decreases in IBs from pre to post (Group A *d* = 1.11; Group B *d* = -.36). | Descriptive statistics and effect sizes used to analyse data. Immediate and small decreases in pre-performance anxiety and psychophysiological measures from pre to post. Mixed findings for performance. Participants reported enhanced understanding of a helpful/unhelp response to a situation and valued coach inclusion in workshop. Participants also reported that one workshop was insufficient for understanding the ABC(DE) framework. |
| Bailey et al. (2023) | NRCT | N = 4 (*n* = 3 males), 100% retention  Coaches; Non-clinical | IBs; iPBI | Pre-, post intervention and follow-up (1 month) | Wellbeing | REBT | 3 x 40-minute sessions (3 weeks) | | Online group sessions (Zoom) | | Visual analysis revealed two out of four participants reduced irrational performance beliefs from pre- to post-intervention. | Visual analysis revealed two out of four participants enhanced mental wellbeing from pre- to post-intervention. |
| Bowman & Turner (2022); UK | NRCT | N = 5 (*n* = 2 males) (59.8 years), 100% retention  Athletes; Non-clinical | IBs; iPBI | 3 timepoints: Pre-, mid- and post-intervention | Affect (golf specific anxiety, social anxiety, wellbeing) | REBT *(n* = 5) | 4 sessions (ranged from 30 minutes - 1.5 hrs) | | Face-to-face and online 1:1 sessions | | Visual analysis revealed that four out of five participants reported reduced irrational performance beliefs from pre- to mid- to post-intervention. | Four out of five participants reported reduced golf-specific anxiety and social anxiety. Three participants reported improved wellbeing with one participant as stable. For participant 5, no change for irrational beliefs, anxiety and wellbeing worsened at follow up. Self-report diaries indicated improved performance and better decision making. One participant still felt frustrated with their game. |
| Chrysidis et al. (2020); UK | NRCT | N = 3 males, 100% retention  Athletes; Non-clinical | IBs; iPBI-2 | 4 time-points: Pre-, mid-, post-intervention and 1-year follow-up | Motivation; Self-perception (self-efficacy); Social validity | REBT with rational self-talk adjunct (*n* = 3) | 5 x 30 min sessions (duration not reported) | | Face-to-face 1:1 sessions | | Significant decreases in IBs from pre to post and follow-up. | Significant increases in motivation and self-efficacy beliefs from pre to post and follow-up. Participants reported that the intervention helped them deal with negative thoughts, shifted their outlook on adversity and improved their confidence, the ability to self-regulate, cope and relax. |
| Cunningham and Turner (2016); UK | NRCT | N = 3 males (23.67 years), 100% retention  Athletes; Non-clinical | RBs and IBs; SGABS  RBs; USAQ | SGABS: Weekly through all phases and six-month follow-up.  USAQ: 3 time-points: Baseline, post-intervention, 2-week follow-up and six-month follow-up | Social validity | REBT (*n* = 3) | 4 sessions (duration not reported) | | Online 1:1 sessions (Skype) | | Significant decreases in total irrationality from pre to post (*d* = .17 to *d* = 1.79). Non-significant increases in unconditional self-acceptance from pre to post. Changes were maintained at follow-up. | Participants reported an increased ability to self-reflect, control their weaknesses and improve performance as well as decreased unhealthy thoughts and emotions. |
| Davis and Turner (2019); UK | NRCT | N = 4 (*n* = 2 males) (41.75 years), 100% retention  Athletes; Non-clinical | IBs; iPBI | 5 time-points: Baseline, post-first session, midpoint, post-final session and 1-month follow-up (data collected over a 20-week period) | Motivation; Sleep; Social validity; Vitality | REBT (*n* = 4) | 5 sessions (5 weeks) | | Face-to-face 1:1 sessions | | Visual analysis revealed decreases in IBs from pre to post which was maintained at follow-up. | Visual analysis revealed increases in self-determination index, vitality and sleep quality from pre to post which were maintained at follow-up. Participants reported changes in thinking and IBs, behaviour and motivation. Intervention was viewed as positive and useful for sport enhancement. |
| Jordana et al. (2022); Spain | NRCT | N = 8 (*n* = 4 males) (41 years), 100% retention  Athletes; Non-clinical | RBs and IBs; SGABS | 2 timepoints; Pre- and post-intervention | Social validity | REBT *(n* = 8) | 3 x 45 minute sessions scheduled every 4 weeks (12 weeks) | | Face-to-face and online 1:1 sessions | | Visual analysis indicated a decrease in irrational beliefs in 6/8 participants from pre to post with an increase in irrational beliefs for 2/8 participants. | At follow-up, 7/8 participants reported they had regained health physical activity. Participants also reported the intervention helped them identify cognitive, emotional and behavioural consequences, set realistic goals, reinterpret the concept of physical activity, develop a more functional internal dialogue and transfer the acquired knowledge to other daily situations. |
| Knapp et al. (2023); UK | NRCT | N = 3 females (30.7 years), 100% retention  Exercisers; Sub-clinical | IBs; iPBI  RBs; USAQ | Three phases:  Pre-intervention (twice per week over 3-week baseline), during (twice per week) and post-intervention (twice per week) | Affect (psychological distress); Dysfunctional behaviours (exercise addiction); Social validity | REBT *(n* = 3) | 6 x 45-minute sessions | | Face-to-face 1:1 sessions | | Significant decreases in irrational beliefs (*d* = 3.60 - *d* = 12.62)  and significant increases in unconditional self-acceptance from pre to post (*d* = -1.03 - *d* = -9.69). Effects maintained at follow-up for two participants. | Significant decreases in exercise addiction and psychological distress from pre to post.  Participants reported that they learnt new coping skills and developed an ability to adopt a rational philosophy to all areas of life. They enjoyed the delivery of sessions and wanted longer to implement behavioural changes. |
| Maxwell-Keys et al. (2022); UK | NRCT | N = 2 (*n* = 1 males) (32 years), 100% retention  Sport officials; Non-clinical | IBs; iPBI | 3 time-points: Pre-, post-intervention and 12-week follow-up | Cognitive (decision making); Emotional (anxiety); Employee performance (match officiating); Social validity | REBT (*n* = 2) | 4 x 45-60 min sessions, delivery of one every two weeks (7 weeks) | | Face-to-face 1:1 sessions | | Significant decreases in IBs from pre to post (Composite IBs participant A; *d* = 2.27; participant B *d* = 2.80) which were maintained at follow-up. | Significant decreases in anxiety and decision reinvestment from pre to post which were maintained at follow-up. Significant increases in match officiating performance from pre to post which was maintained at follow-up. Participants reported that REBT improved psychological wellbeing. |
| Outar et al. (2018); UK | NRCT | N = 3 males (22 years), 100% retention  Exercisers with compulsive exercise traits; Subclinical | IBs; iPBI  RBs (Unconditional Self-Acceptance); USAQ | 19 time-points:  Baseline (twice a week for 3- weeks), intervention (twice a week for 6-weeks) and 2-week follow-up | Dysfunctional behaviours; Social validity | REBT (*n* = 3) | 6 x 45 min weekly sessions (6 weeks) | | Face-to-face 1:1 sessions | | Meaningful reductions in IBs (*d* = 1.80 - *d* = 4.15) and increases in unconditional self-acceptance (*d* = -1.51 - *d* = -.76) for all participants from pre to post to follow-up. | Meaningful reductions in compulsive exercise for all participants from pre to post. Findings maintained at follow-up. Participants reported enhanced emotional and behavioural control which resulted in healthier exercise behaviours and transferred to other life domains. |
| Outar et al. (2021); UK | NRCT | N = 4 (*n* = 2 males) (22.5 years), 100% retention  Exercisers with high muscular dysmorphia; Subclinical | IBs; iPBI  RBs (Unconditional Self-Acceptance); USAQ | 3 phases: Baseline (weekly completion of measures), intervention phase (week 1, 3, 5 and 7) and 6-month follow-up | Mental ill-health (muscle dysmorphia); Social validity | REBT (*n* = 4) | 5 x 1-hr sessions (duration not reported) | | Face-to-face 1:1 sessions | | Meaningful reductions in IBs (*d* = 1.26 - *d* = 2.76) and increases in unconditional self-acceptance (*d* = 1.17 - *d* = 2.48) for all participants from pre to post which was maintained at follow-up. | Meaningful reductions in muscular dysmorphia symptomology for all participants pre to post which was maintained at follow-up. Participants reported improved health behaviours (i.e., eating and exercise), emotions and body image. |
| Turner and Barker (2013); UK | NRCT | N = 4 males (15.5 years), 100% retention  Athletes; Non-clinical | RBs and IBs; SGABS | 18 time-points: Pre-intervention, weekly from week 1 – 17 (includes post-intervention) | Emotional (anxiety); Social validity | REBT (*n* = 4) | 3 x 20 min weekly sessions (3 weeks) | | Face-to-face 1:1 sessions | | Significant decreases in IBs from pre to post in three out of four participants (*d* = 1.05 - *d* = 2.03). | Significant decreases in cognitive anxiety from pre to post in three out of four participants. Participants, parents and coaches reported that the intervention helped the participants feel less anxious, and enhanced cricket performance. |
| Turner and Davis (2019); UK | NRCT | N = 23 (*n* = 13 males) (40.86 years), 91% retention  Athletes; Non-clinical | IBs; iPBI | 5 time-points: Pre-intervention, post-REBT session 3, post-REBT session 5 and 2-week maintenance, 1-week after REPDMS or PDMS, post-intervention. | Motivation; Social validity | REBT + REPDMS  *^c^* (*n* = 12) versus REBT + PDMS  *^c^* (*n* = 11) | 5 x 45 min weekly sessions followed by a one-month break and then 1 x 45 min session (2.5 months) | | Face-to-face group sessions | | Significant decreases in IBs from pre- to post for REBT + REPDMS (*d* = .31)  and REBT + PDMS (*d* = .80). | Significant increases in self-determined motivation from pre- to post for both groups. Participants reported the intervention was useful, encouraged a realistic approach to competing and improved performance. |
| Turner et al. (2018); UK | NRCT | N = 3 males (57.66 years), 100% retention  Athletes; Non-clinical | IBs; iPBI | 6 time-points: Pre-intervention, one week after REBT education, one week after acquisition session 1, one week after acquisition session 3, one week after the integration phase and post-intervention | Emotional (anxiety); Social validity | REBT (*n* = 3) | 6 sessions (≈12 weeks) | | Face-to-face 1:1 sessions | | Visual analysis demonstrated decreases in IBs from pre- to post which were maintained at follow-up. No statistical analysis conducted. | Visual analysis demonstrated decreases in anxiety from pre- to post. No statistical analysis conducted. Participants reported the intervention was positive, helped them feel less anxious and improved their short game in golf. |
| Turner, Slater and Barker (2014b); UK | NRCT | N = 15 males (15.13 years), 100% retention  Athletes; Non-clinical | RBs and IBs; SGABS | 3 time-points: Pre-, post-intervention and 6-week follow-up | Social validity | REBT (*n* = 15) | One-off 1-hr session (1 day) | | Face-to-face group sessions | | Significant decreases in IBs from pre to post (*d* = 1.11) and significant increases in IBs from post to follow-up (*d* = -.72). Decreases in RBs from pre to post (*d* = .04)  and increases from post to follow-up (*d* = -.74). | Participants reported that the intervention helped them to modify their thoughts and behaviours. |
| Urfa et al. (2023); Turkey | NRCT | Five females (28 years) | IBs; iPBI - 2 | 3 timepoints: Pre, mid-, post-intervention | Mental health, cognitive, performance | REBT | REBT and REBT + motivational interviewing | | Online sessions | | Visual analysis revealed decreases in irrational beliefs from pre to post. | Visual analysis revealed decreases in negative emotions, negative automatic thoughts, increases in positive emotions, positive automatic thoughts and perceived performance from pre to mid to post-intervention for both REBT and REBT + Motivational Interviewing (*d* = 3.33 - 9.87). |
| Wood et al. (2017); UK | NRCT | N = 1 female (44 years), 100% retention  Athlete; Non-clinical | RBs and IBs; SGABS | 4 time-points: Pre -, mid-, post-intervention, 3-month follow-up and 6-month follow-up | Self-perception (self-efficacy, control); Sport performance; Social validity | REBT (*n* = 1) | 7 x 1-hr sessions (3-months) | | Face-to-face 1:1 sessions | | Meaningful short and long-term increases in rational beliefs and decreases in IBs from pre to post which were maintained at follow-up. | Meaningful short and long-term improvements in self-efficacy and perceptions of control. Sport performance improvements in competitions. The participant reported that she was able to manage challenging situations, enjoyed competitions, adopted a rational philosophy and her motivation was maintained. |
| Wood, Barker, Turner and Sheffield (2018); UK | NRCT | N = 8 (*n* = 5 males) (40.12 years), 87.5% retention  Athlete; Non-clinical | RBs and IBs; SGABS | Weekly from the onset (at least five weeks prior to beginning to intervention to five weeks after intervention) | Achievement goals; Emotional; Psychophysiological; Sport performance; Social validity | REBT (*n* = 8) | 5 x 30 min sessions, delivery of once/twice weekly (3-5 weeks) | | Face-to-face 1:1 sessions | | Short and long-term reductions in IBs (*d* = .58 - *d* = 6.36) throughout the intervention which were maintained follow-up. | Reductions in psychophysiological outcomes and avoidance goals. Improvements in athletic performance.  Mixed findings for anxiety. Participants, head coach and sport psychologist reported an uptake of a rational philosophy, improved confidence, management of emotions and thoughts and performance. |
| Wood et al. (2020); UK | NRCT | N = 1 male (22 years), 100% retention  Athlete; Non-clinical | IBs; iPBI | Session-by-session basis as well as 6-month time-point | Sport performance | REBT + Motivational Interviewing (*n* = 1) | 8 sessions (duration not reported) | | Face-to-face 1:1 sessions | | Short and long-term reductions in IBs throughout the intervention which were maintained follow-up. | Substantial increases in self-determined motivation that was maintained at follow-up. Improved performance from pre to post. |
| Deen et al. (2017); Malaysia | MMS | N = 5 (*n* = 3 males) (19.7 years), 100% retention  Athletes; Non-clinical | IBs; iPBI | ≈32 time-points: Twice weekly through baseline, intervention and post-intervention and 4-week follow-up | Resilience; Social validity | REBT (*n* = 5) | 5 x 1hr sessions (duration not reported) | | Face-to-face 1:1 sessions | | Significant decreases in IBs from pre to post-intervention (*d* = 1.26 to 3.02). | Significant increases in resilient qualities for 3/5 participants from pre to post. Participants reported improvements in handling adversity, being flexible and rational. The intervention was viewed as interesting, unique and useful. |
| Kara et al. (2023); Turkey | MMS | N = 22 (*n* = 8 males) (19.9 years), 100% retention  Athletes; Non-clinical | IBs; B IBS (2) | 2 timepoints;  Pre-, mid- and post-intervention | Affect (anxiety); Social validity | REBT (n = 11) versus Wait list control (*n* = 11) | 8 x 1.5-hr weekly sessions (8 weeks) | | Face-to-face group sessions | | Significant decreases in irrational beliefs from pre to post for REBT group *(d* = 1.55). No significant changes for control group *(d* = .23). | Significant decreases in anxiety from pre to post for REBT group. No significant changes for control group. Participants reported that the intervention encouraged a rational philosophy and that the group format offered support and normalization of pre-competition anxiety. |

*Note:* *^a^* N is the initial sample size for the intervention and control condition (if relevant). *^b^* For the ‘Need for Approval’ subscale on SGABS, an additional time point was added, 4.5-month follow-up. *^c^* Intervention is based upon REBT principles.

General: IBs = Irrational beliefs; NRCT = Non-randomised control trial; MMS = mixed method study; PDMS = Personal Disclosure Mutual Sharing; RBs = Rational Beliefs; RBS = Rational beliefs; RCT = randomised control trial; REBT = Rational Emotive Behaviour Therapy; REPDMS = Rational Emotive Personal Disclosure Mutual Sharing.

Measures: B ISB (2); The Brief Irrational Beliefs Scale; iPBI = The Irrational Performance Beliefs Inventory; iPBI - 2 = The Irrational Performance Beliefs Inventory 2; SGABS = General Attitudes and Beliefs Scale- Short Form; USAQ = Unconditional Self-Acceptance Questionnaire.
