# Supplementary material for: A systematic review of the nature and efficacy of Rational Emotive Behaviour Therapy interventions
Source: PLoS One. 2024 Jul 9;19(7):e0306835. doi: 10.1371/journal.pone.0306835 (PMC11232995; doi:10.1371/journal.pone.0306835)
Supplement: S3 File — (DOCX) [file pone.0306835.s003.docx]

**S3 File**

**Methodological Appraisal**

**Table 13**

*Methodological Quality of the Included Randomised Controlled Trials (n = 82 studies)*

| **Study; Domain** | **1** | **2** | **3** | **4** | **5** | **MMAT score (%)** |
| --- | --- | --- | --- | --- | --- | --- |
| **Education** | | | | | | |
| Arnkoff (1986) | CT | CT | Yes | Yes | CT | 40 |
| Boutin and Tosi (1986) | CT | Yes | No | No | CT | 20 |
| Chukwuma et al. (2023) | Yes | Yes | CT | No | CT | 40 |
| Cramer and Fong (1991) | Yes | No | Yes | Yes | CT | 60 |
| Crum and College (2016) | CT | CT | Yes | CT | CT | 20 |
| David et al. (2018) | Yes | Yes | Yes | CT | CT | 60 |
| David et al. (2022) | CT | No | CT | No | Yes | 20 |
| Decker and Russell (1981) | No | Yes | Yes | No | CT | 40 |
| Ede et al. (2021) | CT | Yes | Yes | No | Yes | 60 |
| Horan (1996) | CT | No | Yes | Yes | CT | 40 |
| Hymen and Warren (1978) | CT | CT | Yes | CT | CT | 20 |
| Ifeanyieze et al. (2021) | Yes | Yes | Yes | CT | Yes | 80 |
| Jalali et al. (2014) | CT | Yes | Yes | CT | CT | 40 |
| Leaf, et al. (1986) | CT | CT | CT | CT | CT | 0 |
| Mahfar et al. (2014) | CT | CT | CT | CT | CT | 0 |
| Miller and Kassinove (1978) | CT | Yes | CT | CT | CT | 20 |
| Möller et al. (1993) | CT | No | CT | CT | CT | 0 |
| Nielsen et al. (1996) | CT | Yes | Yes | Yes | CT | 60 |
| Ofoegbu et al. (2020) | Yes | CT | Yes | CT | CT | 40 |
| Ogbuanya et al. (2018) | Yes | Yes | Yes | CT | CT | 60 |
| Onyemaechi et al. (2023) | Yes | CT | Yes | Yes | CT | 60 |
| Popa et al. (2022) | CT | Yes | Yes | No | CT | 40 |
| Roman (2011) | CT | Yes | Yes | Yes | CT | 60 |
| Rosenbaum et al. (1991) | CT | Yes | Yes | Yes | CT | 60 |
| Sahin and Türk (2021) | Yes | CT | Yes | CT | CT | 40 |
| Sheey and Horan (2004) | CT | Yes | Yes | CT | Yes | 60 |
| Sousa and Padovani (2021) | CT | Yes | Yes | CT | CT | 40 |
| Tomoiaga et al. (2022) | CT | CT | Yes | No | Yes | 40 |
| Trexler and Karst (1972) | CT | Yes | Yes | CT | CT | 40 |
| Türkum (2007) | CT | Yes | Yes | Yes | Yes | 80 |
| Ugwoke et al. (2021) | Yes | Yes | Yes | Yes | CT | 80 |
| Victor-Aigbodion et al. (2023) | Yes | Yes | Yes | No | CT | 60 |
| Warren et al. (1984) | Yes | CT | CT | Yes | CT | 40 |
| Wessel and Mersch (1994) | CT | Yes | Yes | CT | CT | 40 |
| Xu and Liu (2017) | Yes | Yes | Yes | CT | CT | 60 |
| **Education Overall Appraisal (*n* = 35)** | | | | | | **43** |
| **Forensic (*n* = 1)** | | | | | | |
| Aldahadha (2018) | CT | Yes | Yes | No | CT | 40 |
| **Hospital and Community Healthcare** | | | | | | |
| Cramer and Kupshik (1993) | Yes | Yes | Yes | CT | CT | 60 |
| Emmelkamp and Beens (1991) | CT | Yes | No | Yes | Yes | 60 |
| Emmelkamp et al. (1986) | CT | CT | No | Yes | CT | 20 |
| Emmelkamp, Mersch, Vissia and Van Der Helm (1985) | CT | CT | Yes | Yes | CT | 40 |
| Emmelkamp et al. (1988) | CT | Yes | Yes | Yes | Yes | 80 |
| Kassinove et al. (1980) | Yes | No | CT | CT | Yes | 40 |
| Komasi et al. (2017) | Yes | Yes | Yes | Yes | CT | 80 |
| Lipsky et al. (1980) and Smith (1983) | CT | No | CT | Yes | CT | 20 |
| Mattick and Peters (1988) | CT | Yes | Yes | Yes | Yes | 80 |
| Mattick et al. (1989) | CT | Yes | Yes | Yes | Yes | 80 |
| Moon et al. (2021) | Yes | Yes | Yes | Yes | Yes | 100 |
| Munjack et al. (1984) | CT | No | Yes | Yes | CT | 40 |
| Rezaeisharif (2021) | No | CT | CT | CT | CT | 0 |
| Szentagotai et al. (2008) | CT | Yes | Yes | Yes | Yes | 80 |
| **Hospital and Community Healthcare Overall Appraisal (*n* = 14)** | | | | | | **56** |
| **Organisational** | | | | | | |
| Ekwueme et al. (2023) | Yes | Yes | Yes | No | CT | 60 |
| Ifelunni et al. (2022) | CT | Yes | CT | CT | CT | 20 |
| Iremeka et al. (2021) | Yes | Yes | Yes | No | CT | 60 |
| Möller and Botha (1996) | CT | Yes | Yes | Yes | CT | 60 |
| Ogbuanya et al. (2017) | Yes | Yes | Yes | CT | CT | 60 |
| Onuigbo et al. (2018) | Yes | Yes | CT | Yes | CT | 60 |
| Otu and Omeje (2021) | Yes | Yes | Yes | Yes | CT | 80 |
| Stanton (1989) | No | Yes | CT | CT | CT | 20 |
| Thurman (1985a; 1985b) | CT | Yes | Yes | Yes | CT | 60 |
| Ugwoke et al. (2017) | Yes | Yes | Yes | Yes | CT | 80 |
| **Organisational Overall Appraisal (*n* = 10)** | | | | | | **56** |
| **Relationships** | | | | | | |
| Baucom and Lester (1986) | CT | CT | No | CT | CT | 0 |
| David (2014) | CT | CT | Yes | CT | CT | 20 |
| David et al. (2017) | CT | Yes | No | CT | No | 20 |
| Ede & Okeke (2022) | CT | Yes | Yes | Yes | CT | 60 |
| Gavita and Calin (2013) | Yes | Yes | Yes | CT | CT | 60 |
| Huber and Milstein (1985) | CT | Yes | Yes | CT | CT | 40 |
| Joyce (1995) | Yes | Yes | CT | CT | CT | 40 |
| **Relationships Overall Appraisal (*n* = 7)** | | | | | | **34** |
| **Self-identified Healthcare Need** | | | | | | |
| Artiran and DiGiuseppe (2021) | No | Yes | Yes | CT | CT | 40 |
| Biran and Wilson (1981) | CT | Yes | Yes | Yes | CT | 60 |
| Eseadi et al. (2016) | CT | Yes | Yes | CT | CT | 40 |
| Hovland (1995) | CT | Yes | No | CT | CT | 20 |
| Johnson and Ridley (1992) | Yes | Yes | CT | CT | CT | 40 |
| Kanter and Goldfried (1979) | CT | Yes | Yes | Yes | CT | 60 |
| Lake et al. (1979) | CT | CT | CT | CT | Yes | 20 |
| Mersch (1995) | CT | Yes | Yes | CT | CT | 40 |
| Omeje et al. (2018) | Yes | Yes | Yes | Yes | CT | 80 |
| Syzmanski and O'Donohue (1995) | No | CT | Yes | Yes | CT | 40 |
| Warren et al. (1988) | CT | Yes | Yes | CT | CT | 40 |
| **Self-identified Healthcare Need Overall Appraisal (*n* = 11)** | | | | | | **44** |
| **Sport and Exercise** | | | | | | |
| Nejati et al. (2022) | Yes | CT | Yes | CT | CT | 40 |
| Turner, Slater and Barker (2014a) | Yes | CT | CT | Yes | CT | 40 |
| Vertopoulos and Turner (2017) | Yes | Yes | Yes | No | Yes | 80 |
| Wood (2018) | Yes | Yes | Yes | Yes | Yes | 100 |
| **Sport and Exercise Overall Appraisal (*n* = 4)** | | | | | | **65** |
| **RCT Overall Appraisal (*n* = 82)** | | | | | | **47** |

*Note:* CT = Can’t tell whether criterion is met due to insufficient information; MMAT = Mixed Methods Appraisal Tool; No = Criterion not met; Yes = Criterion met; 20% = One criterion met; 40% = Two criteria met; 60% = Three criteria met; 80%= Four criteria met; 100% = Five criteria met; 1 = ‘Is randomisation appropriately performed?’; 2 = ‘Are the groups comparable at baseline?’; 3 = ‘Are there complete outcome data? (Yes if ≥ 80)’; 4 = ‘Are outcome assessors blinded to the intervention provided?’; 5 = ‘Did the participants adhere to the assigned intervention?’

**Table 14**

*Methodological Quality of the Included Non-randomised Controlled Trials (n = 77 studies)*

| **Study; Domain** | **1** | **2** | **3** | **4** | **5** | **MMAT score (%)** |
| --- | --- | --- | --- | --- | --- | --- |
| **Education** | | | | | | |
| Bedel, Ercan and Sahan (202) | CT | Yes | CT | CT | CT | 20 |
| Boutin (1978) | Yes | Yes | Yes | CT | CT | 60 |
| Caruso et al. (2018) | Yes | No | CT | CT | CT | 20 |
| Cristea et al. (2006; 2008) | Yes | Yes | CT | Yes | CT | 60 |
| David & Cobeanu (2016) | Yes | Yes | Yes | CT | CT | 60 |
| David et al. (2021) | Yes | Yes | CT | CT | Yes | 60 |
| David et al. (2022) | Yes | Yes | Yes | CT | Yes | 80 |
| Flanagan et al. (1998) | CT | Yes | Yes | CT | CT | 40 |
| Jacobs and Croake (1976) | No | Yes | Yes | No | CT | 40 |
| Kabasakal and Emiroğlu (2020) | Yes | Yes | CT | CT | CT | 40 |
| Lupu and Iftene (2009) | Yes | Yes | Yes | CT | CT | 60 |
| McCormick et al. (1991) | Yes | Yes | CT | CT | CT | 40 |
| Mio and Matsumuto (2018) | Yes | Yes | Yes | CT | CT | 60 |
| Morris (1993) | Yes | Yes | CT | CT | CT | 40 |
| Popa & Predatu (2019) | Yes | Yes | CT | CT | CT | 40 |
| Schenk et al. (2020) | Yes | Yes | CT | CT | CT | 40 |
| Thorpe et al. (1984) Study 1 | No | No | CT | Yes | CT | 20 |
| Thorpe et al. (1984) Study 2 | No | No | CT | Yes | CT | 20 |
| Thurman (1983) | Yes | Yes | Yes | Yes | CT | 80 |
| Trip et al. (2010) | No | Yes | CT | Yes | CT | 40 |
| Ulusoy and Duy (2013) | No | Yes | Yes | Yes | CT | 60 |
| Vaida et al. (2008) | Yes | Yes | Yes | Yes | CT | 80 |
| Wilde (1996) | CT | CT | CT | CT | CT | 0 |
| Wilde (1996; 1999) | CT | Yes | Yes | CT | CT | 40 |
| **Education Overall Appraisal (*n* = 24)** | | | | | | **46** |
| **Hospital and Community Healthcare** | | | | | | |
| Emmelkamp, Mersch, Vissia and Van Der Helm (1985) | CT | No | CT | CT | CT | 0 |
| Grove et al. (2021) | Yes | Yes | No | Yes | CT | 60 |
| Grove et al. (2023) | Yes | Yes | CT | Yes | CT | 60 |
| Hamberger and Lohr (1980) | Yes | No | Yes | CT | CT | 40 |
| Igna et al. (2014) | Yes | Yes | Yes | Yes | CT | 80 |
| Jacobsen et al. (1987) | Yes | CT | No | Yes | CT | 40 |
| Mersch et al. (1989; 1991) | Yes | No | No | Yes | CT | 40 |
| Neamtu and David (2016) | Yes | Yes | Yes | Yes | CT | 80 |
| Nottingham and Neimeyer (1992) | Yes | CT | CT | CT | CT | 20 |
| Ray (1984) | No | Yes | CT | CT | CT | 20 |
| Riggs and Meyer (1981) | No | No | No | No | CT | 0 |
| **Hospital and Community Healthcare Overall Appraisal (*n* = 11)** | | | | | | **40** |
| **Organisational** | | | | | | |
| Bora et al. (2013) | Yes | Yes | Yes | No | CT | 60 |
| David and Matu (2013) | Yes | Yes | No | No | CT | 40 |
| David et al. (2016) | Yes | Yes | CT | CT | CT | 40 |
| De Jesus and Conboy (2001) | Yes | CT | CT | CT |  | 20 |
| Ellis et al. (1989) | No | Yes | No | CT | CT | 20 |
| Forman and Forman (1980) | No | Yes | Yes | No | CT | 40 |
| Kushnir and Malkinson (1993) | Yes | Yes | Yes | CT | CT | 60 |
| Kushnir et al. (1998) | Yes | Yes | CT | CT | CT | 40 |
| Kushnir et al. (1994) | Yes | No | CT | CT | CT | 20 |
| Morris (1992) | Yes | No | Yes | CT | CT | 40 |
| Turner and Barker (2015) | CT | Yes | Yes | CT | CT | 40 |
| Wood et al. (2021) | Yes | Yes | CT | Yes | CT | 60 |
| Woods (1987) | Yes | CT | Yes | CT | CT | 40 |
| **Organisational Overall Appraisal (*n* = 13)** | | | | | | **40** |
| **Relationships** | | | | | | |
| Cramer (2005) | Yes | Yes | Yes | Yes | Yes | 100 |
| McNaughton-Cassil (2002) | Yes | Yes | Yes | Yes | CT | 80 |
| Mueller and Moskowitz (2020) | Yes | Yes | Yes | CT | Yes | 80 |
| Trip et al. (2019) | Yes | Yes | Yes | CT | CT | 60 |
| Yu & Schill (1976) | No | Yes | CT | CT | CT | 20 |
| **Relationships Overall Appraisal (*n* = 5)** | | | | | | **68** |
| **Self-identified Healthcare Need** | | | | | | |
| Adekoya et al. (2023) | CT | CT | CT | CT | CT | 0 |
| Keller et al. (1975) | Yes | Yes | CT | Yes | CT | 60 |
| Kirkby (1984) | CT | Yes | Yes | CT | Yes | 60 |
| McKnight (1984) | Yes | Yes | CT | Yes | CT | 60 |
| Newhouse and Schwager (1978) | No | Yes | CT | CT | CT | 20 |
| Pasarelu et al. (2021) | Yes | Yes | Yes | CT | Yes | 80 |
| **Self-identified Healthcare Need Overall Appraisal (*n* = 6)** | | | | | | **47** |
| **Sport and Exercise** | | | | | | |
| Bailey et al. (2023) | Yes | Yes | Yes | No | CT | 60 |
| Bowman et al. (2022) | Yes | Yes | Yes | CT | Yes | 80 |
| Chrysidis et al. (2020) | Yes | Yes | Yes | CT | CT | 60 |
| Cunningham and Turner (2016) | Yes | Yes | No | CT | CT | 40 |
| Davis and Turner (2019) | Yes | Yes | Yes | CT | CT | 60 |
| Jordana et al. (2022) | Yes | Yes | Yes | CT | CT | 60 |
| Knapp et al. (2023 | Yes | Yes | Yes | Yes | Yes | 100 |
| Maxwell-Keys (2022) | Yes | Yes | Yes | Yes | Yes | 100 |
| Outar et al. (2018) | Yes | Yes | Yes | CT | CT | 60 |
| Outar et al. (2021) | Yes | Yes | Yes | CT | Yes | 80 |
| Turner and Barker (2013) | Yes | Yes | Yes | Yes | Yes | 100 |
| Turner and Davis (2019) | Yes | Yes | Yes | Yes | CT | 80 |
| Turner et al. (2018) | Yes | Yes | Yes | CT | CT | 60 |
| Turner et al. (2014b) | Yes | Yes | Yes | CT | CT | 60 |
| Urfa et al. (2023) | Yes | Yes | CT | CT | CT | 40 |
| Wood et al. (2017) | Yes | Yes | Yes | CT | CT | 60 |
| Wood et al. (2018) | Yes | Yes | Yes | CT | Yes | 80 |
| Wood et al. (2020) | Yes | Yes | Yes | CT | Yes | 80 |
| **Sport and Exercise Overall Appraisal (*n* = 18)** | | | | | | **70** |
| **NRCT Overall Appraisal (*n* = 77)** | | | | | | **51** |

*Note:* CT = Can’t tell whether criterion is met due to insufficient information; MMAT = Mixed Methods Appraisal Tool; No = Criterion not met; NRCT = Non-randomised control trials; Yes = Criterion met; 20% = One criterion met; 40% = Two criteria met; 60% = Three criteria met; 80% = Four criteria met; 100% = Five criteria met; 1 = ‘Are the participants representative of the target population?’; 2 = ‘Are measurements appropriate regarding both the outcome and intervention (or exposure)?’; 3 = ‘Are there complete outcome data (Yes if ≥ 80)?’; 4 = ‘Are the confounders accounted for in the design and analysis?’; 5 = ‘During the study period, is the intervention administered (or exposure occurred) as intended?’

**Table 15**

*Methodological Quality of the Included Mixed Methods Studies (n = 3 studies)*

| **Study; domain** | **RCT** | | | | | **NRS** | | | | | **QS** | | | | | **MMS** | | | | | **MMAT score (%)** |
| --- | --- | --- | --- | --- | --- | --- | --- | --- | --- | --- | --- | --- | --- | --- | --- | --- | --- | --- | --- | --- | --- |
|  | **1** | **2** | **3** | **4** | **5** | **6** | **7** | **8** | **9** | **10** | **11** | **12** | **13** | **14** | **15** | **16** | **17** | **18** | **19** | **20** |  |
| **Organisational (*n* = 1)** | | | | | | | | | | | | | | | | | | | | | |
| Jones et al. (2020) | Yes | Yes | Yes | Yes | CT |  | | | | | Yes | Yes | Yes | Yes | Yes | Yes | Yes | Yes | Yes | Yes | 100 |
| **Sport and Exercise** | | | | | | | | | | | | | | | | | | | | | |
| Deen et al. (2017) |  | | | | | Yes | Yes | Yes | Yes | CT | Yes | Yes | Yes | Yes | Yes | Yes | Yes | Yes | Yes | Yes | 100 |
| Kara et al. (2023) |  | | | | | Yes | Yes | Yes | CT | CT | Yes | Yes | CT | No | Yes | Yes | Yes | Yes | Yes | No | 80 |
| **Sport and Exercise Overall Appraisal (*n* = 2)** | | | | | | | | | | | | | | | | | | | | | **90** |
| **MMS Overall Appraisal (*n* = 3)** | | | | | | | | | | | | | | | | | | | | | **93** |

*Note:* CT = Can’t tell whether criterion is met due to insufficient information; MMAT = Mixed Methods Appraisal Tool; MMS = Mixed methods study; No = Criterion not met; NRS = Non-randomised study; QS = Qualitative study; RCT = Randomised control trial; Yes = Criterion met; 20% = One criterion met; 40% = Two criteria met; 60% = Three criteria met; 80% = Four criteria met; 100% = Five criteria met; 1 = ‘Is randomisation appropriately performed?’; 2 = ‘Are the groups comparable at baseline?’; 3 = ‘Are there complete outcome data? (Yes if ≥ 80)’; 4 = ‘Are outcome assessors blinded to the intervention provided?’; 5 = ‘Did the participants adhere to the assigned intervention?’; 6 = ‘Are the participants representative of the target population?’; 7 = ‘Are measurements appropriate regarding both the outcome and intervention (or exposure)?’; 8 = ‘Are there complete outcome data (Yes if ≥ 80)?’; 9 = ‘Are the confounders accounted for in the design and analysis?’; 10 = ‘During the study period, is the intervention administered (or exposure occurred) as intended?’; 11 = ‘Is the qualitative approach appropriate to the research question?’; 12 = ‘Are the qualitative data collection methods adequate to address the research question?’; 13 = ‘Are the findings adequately derived from the data?’; 14 = ‘Is the interpretation of results sufficiently substantiated by data?’; 15 = ‘Is there coherence between qualitative data sources, collection, analysis and interpretation?’; 16 = ‘Is there adequate rationale for using a mixed methods design to address the research question?’; 17 = ‘Are the different components of the study effectively integrated to answer the research question?’; 18 = ‘Are the outputs of the integration of qualitative and quantitative components adequately interpreted?’; 19 = ‘Are divergencies and inconsistencies between quantitative and qualitative results adequately addressed?’; 20 = ‘Do the different components of the study adhere to the quality criteria of each tradition of the methods involved?’
