# Supplementary material for: A systematic review of the nature and efficacy of Rational Emotive Behaviour Therapy interventions
Source: PLoS One. 2024 Jul 9;19(7):e0306835. doi: 10.1371/journal.pone.0306835 (PMC11232995; doi:10.1371/journal.pone.0306835)
Supplement: S4 File — (DOCX) [file pone.0306835.s004.docx]

**S4 File**

**References Included in the Systematic Review**

Adekoya, A. F., Adams, A. B., Okueso, S. A., Ajani, O. E., Okparaeke, M. I., Onyekwere, O. K., Ugwu, N. D., Oloyede, A. O., & Alade, T. T. (2023). Promoting parent-to-child sex education in Nigerian homes through rational-emotive health education programme: A pilot study of Imojo Community Ofekiti State, Nigeria. *Journal of Home Economics Research*, *30*(1), 160-173.

Aldahadha, B. (2018). Disputing irrational beliefs among convicted terrorists and extremist beliefs. *Journal of Rational-Emotive and Cognitive-Behavior Therapy, 36*(4), 404-417. <https://doi.org/10.1007/s10942-018-0293-7>

Arnkoff, D. B. (1986). A comparison of the coping and restructuring components of cognitive restructuring. *Cognitive Therapy and Research*, *10*(2), 147-158. <https://doi.org/10.1007/BF01173721>

Artiran, M., & DiGiuseppe, R. (2022). Rational emotive behavior therapy compared to client-centered therapy for outpatients: A randomized clinical trial with a three months follow up. *Journal of Rational-Emotive and Cognitive-Behavior Therapy*, *40*(2), 206-233. <https://doi.org/10.1007/s10942-021-00408-0>

Bailey, R., & Turner, M. (2023). The effects of a brief online rational emotive behavioural therapy (REBT) on coach wellbeing and coaching behaviour. *The Sport Psychologist* *37*(4), 266-273. <https://doi.org/10.1123/tsp.2023-0009>

Baucom, D. H., & Lester, G. W. (1986). The usefulness of cognitive restructuring as an adjunct to behavioral marital therapy. *Behavior Therapy, 17*(4), 385-403. <https://doi.org/10.1016/S0005-7894(86)80070-3>

Bedel, A., Ercan, A. B., & Şahan, B. (2020). The effect of test anxiety psychoeducation program on test anxiety and irrational beliefs. *Education and Science, 45*(203), 275-286. <https://doi.org/10.15390/EB.2020.8570>

Biran, M., & Wilson, G. T. (1981). Treatment of phobic disorders using cognitive and exposure methods: A self-efficacy analysis. *Journal of Consulting and Clinical Psychology, 49*(6), 886-899. <https://doi.org/10.1037/0022-006X.49.6.886>

Bora, C. H., Vernon, A., & Trip, S. (2013). Effectiveness of a rational emotive behavior education program in reducing teachers' emotional distress. *Journal of Cognitive and Behavioral Psychotherapies, 13*(2), 585-604.

Boutin, G. E. (1978). Treatment of test anxiety by rational stage directed hypnotherapy: A case study. *American Journal of Clinical Hypnosis, 21*(1), 52-57. <https://doi.org/10.1080/00029157.1978.10403958>

Boutin, G. E., & Tosi, D. J. (1983). Modification of irrational ideas and test anxiety through rational stage directed hypnotherapy [RSDH]. *Journal of Clinical Psychology, 39*(3), 382-391. [https://doi.org/10.1002/1097-4679(198305)39:3<382::AID-JCLP2270390312>3.0.CO;2-L](https://doi.org/10.1002/1097-4679(198305)39:3%3c382::AID-JCLP2270390312%3e3.0.CO;2-L)

Bowman, A. W., & Turner, M. J. (2022). When time is of the essence: The use of rational emotive behavior therapy (REBT) informed single-session therapy (SST) to alleviate social and golf-specific anxiety, and improve wellbeing and performance, in amateur golfers. *Psychology of Sport and Exercise, 60*, 102167. <https://doi.org/10.1016/j.psychsport.2022.102167>

Caruso, C., Angelone, L., Abbate, E., Ionni, V., Biondi, C., Di Agostino, C., Mobili, A., Verità, R., Navarra, R., Ruggiero, G. M., & Mezzaluna, C. (2018). Effects of a REBT based training on children and teachers in primary school. *Journal of Rational-Emotive and Cognitive-Behavior Therapy, 36*(1), 1-14. <https://doi.org/10.1007/s10942-017-0270-6>

Chrysidis, S., Turner, M. J., & Wood, A. G. (2020). The effects of REBT on irrational beliefs, self-determined motivation, and self-efficacy in American Football*. Journal of Sports Sciences, 38*(19), 2215-2224. <https://doi.org/10.1080/02640414.2020.1776924>

Chukwuma, J. N., Areji, A. C., Obumse, N. A., Eze, E., Amadi, K., Agbo, C. O., Nweze, C. A., Eze, C. F., Omeh, S. O., Elom, C. O., Ari, A. O., Obeagu, E. I., & Omeje, G. N. (2023). Impact of exposure to rational career reflective training on work-related ethics among student-philosophers. *Medicine*, *102*(42), e35608. <https://doi.org/10.1097/MD.0000000000035608>

Cramer, D. (2005). Effect of four aspects of rational statements on expected satisfaction with a close relationship. *British Journal of Guidance and Counselling, 33*(2), 227-238. <https://doi.org/10.1080/03069880500132763>

Cramer, D., & Fong, J. (1991). Effect of rational and irrational beliefs on intensity and “inappropriateness” of feelings: A test of rational-emotive theory. *Cognitive Therapy and Research, 15*(4), 319-329. <https://doi.org/10.1007/BF01205176>

Cramer, D., & Kupshik, G. (1993). Effect of rational and irrational statements on intensity and ‘inappropriateness' of emotional distress and irrational beliefs in psychotherapy patients. *British Journal of Clinical Psychology, 32*(3), 319-325. <https://doi.org/10.1111/j.2044-8260.1993.tb01062.x>

Cristea, I. A., Benga, O., & Opre, A. (2006). The comparative efficiency of a rational emotive educational intervention for anxiety in 3rd grade children: An analysis of relevant developmental constraints. *Cognition, Brain, Behavior, 10*(4), 637-657.

Cristea, I. A., Benga, O., & Opre, A. (2008). The implementation of a rational-emotive educational intervention for anxiety in a 3rd grade classroom: An analysis of relevant procedural and developmental constraints. *Journal of Evidence-Based Psychotherapies, 8*(1), 31-51.

Crum, J. I., & James, E. (2016). Effects of priming dialectic rational beliefs on irrational beliefs. *Psi Chi Journal of Psychological Research, 21*(1), 46-53.

Cunningham, R., & Turner, M. J. (2016). Using rational emotive behavior therapy (REBT) with mixed martial arts (MMA) athletes to reduce irrational beliefs and increase unconditional self-acceptance. *Journal of Rational-Emotive and Cognitive-Behavior Therapy, 34*(4), 28-309. <https://doi.org/10.1007/s10942-016-0240-4>

David, O. A. (2014). The rational positive parenting program for child externalizing behavior: Mechanisms of change analysis. *Journal of Evidence-Based Psychotherapies, 14*(1) 21-38.

David, O. A., & Cobeanu, O. (2016). Evidence-based training in cognitive-behavioural coaching: Can personal development bring less distress and better performance? *British Journal of Guidance and Counselling, 44*(1), 12-25. <https://doi.org/10.1080/03069885.2014.1002384>

David, O. A., & David, D. (2022). How can we best use technology to teach children to regulate emotions? Efficacy of the cognitive reappraisal strategy based on robot versus cartoons versus written statements in regulating test anxiety. *Journal of Rational-Emotive and Cognitive-Behavior Therapy, 40*(4), 793-802. <https://doi.org/10.1007/s10942-021-00440-0>

David, O. A., & Matu, S. A. (2013). How to tell if managers are good coaches and how to help them improve during adversity? The managerial coaching assessment system and the rational managerial coaching program. *Journal of Cognitive and Behavioral Psychotherapies, 13*(2), 259-274.

David, O. A., Capris, D., & Jarda, A. (2017). Online coaching of emotion-regulation strategies for parents: Efficacy of the online rational positive parenting program and attention bias modification procedures. *Frontiers in Psychology, 8*(500), 1-10. <https://doi.org/10.3389/fpsyg.2017.00500>

David, O. A., Cardoș, R. A., & Matu, S. (2019). Changes in irrational beliefs are responsible for the efficacy of the REThink therapeutic game in preventing emotional disorders in children and adolescents: Mechanisms of change analysis of a randomized clinical trial. *European Child and Adolescent Psychiatry, 28*, 307-318. <https://doi.org/10.1007/s00787-018-1195-z>

David, O. A., Ionicioiu, I., Imbăruş, A. C., & Sava, F. A. (2016). Coaching banking managers through the financial crisis: Effects on stress, resilience, and performance. *Journal of Rational-Emotive and Cognitive-Behavior therapy, 34*(4), 267-281. <https://doi.org/10.1007/s10942-016-0244-0>

David, O. A., Magurean, S., & Tomoiagă, C. (2022). Do improvements in therapeutic game-based skills transfer to real life improvements in children's emotion-regulation abilities and mental health? A pilot study that offers preliminary validity of the REThink in-game performance scoring. *Frontiers in Psychiatry, 13*, 828481. <https://doi.org/10.3389/fpsyt.2022.828481>

David, O. A., Predatu, R., & Cardoș, R. A. (2021). Effectiveness of the REThink therapeutic online video game in promoting mental health in children and adolescents. *Internet Interventions, 25*(100391), 1-5. <https://doi.org/10.1016/j.invent.2021.100391>

Davis, H., & Turner, M. J. (2020). The use of rational emotive behavior therapy (REBT) to increase the self-determined motivation and psychological well-being of triathletes. *Sport, Exercise, and Performance Psychology, 9*(4), 489-505. <https://doi.org/10.1037/spy0000191>

De Jesus, S. N., & Conboy, J. (2001). A stress management course to prevent teacher distress. *International Journal of Educational Management, 15*(3), 131-137

Decker, T. W., & Russell, R. K. (1981). Comparison of cue-controlled relaxation and cognitive restructuring versus study skills counseling in treatment of test-anxious college underachievers. *Psychological Reports, 49*(2), 459–469. <https://doi.org/10.2466/pr0.1981.49.2.459>

Deen, S., Turner, M. J., & Wong, R. S. K. (2017). The effects of REBT, and the use of credos, on irrational beliefs and resilience qualities in athletes. *The Sport Psychologist, 31*(3), 249–263. <https://doi.org/10.1123/tsp.2016-0057>

Ede, M. O., & Okeke, C. I. (2022). Testing the impacts of rational-emotive couple intervention in a sample of parents seeking divorce. *Psychological Reports, 0*, 1-26. <https://doi.org/10.1177/00332941221139994>

Ede, M. O., Okeke, C. I., & Chukwu, C. L. (2021). Assessing the efficacy of rational emotive behavior intervention for visually impaired upper basic school children with negative self-belief/personal value system. *Journal of Rational-Emotive and Cognitive-Behavior Therapy, 40*, 452-473. <https://doi.org/10.1007/s10942-021-00419-x>

Ekwueme, H. U., Ede, M. O., Eze, E. C., Mezieobi, D. I., Aroh, P. N., Oneli, J. O., Nweke, P. O., & Enyi, C. (2023). Impact of occupational health coaching on irrational career beliefs and workplace deviant behaviors among school employees: Implications for Educational administrative policy. *Medicine*, *102*(22), e33685. <https://doi.org/10.1097/MD.0000000000033685>

Ellis, A., Sichel, J., Leaf, R. C., & Mass, R. (1989). Countering perfectionism in research on clinical practice I: Surveying rationality changes after a single intensive RET intervention. *Journal of Rational-Emotive and Cognitive-Behavior Therapy, 7*(4), 197–218. <https://doi.org/10.1007/BF01073808>

Emmelkamp, P. M., & Beens, H. (1991). Cognitive therapy with obsessive-compulsive disorder: A comparative evaluation. *Behaviour Research and Therapy, 29*(3), 29-300.  <https://doi.org/10.1016/0005-7967(91)90120-R>

Emmelkamp, P. M., Brilman, E., Kuiper, H., & Mersch, P. (1986). The treatment of agoraphobia: A comparison of self-instructional training, rational emotive therapy, and exposure in vivo. *Behavior Modification, 10*(1), 37–53. <https://doi.org/10.1177/01454455860101003>

Emmelkamp, P. M., Mersch, P.-P., & Vissia, E. (1985). The external validity of analogue outcome research: Evaluation of cognitive and behavioral interventions. *Behaviour Research and Therapy, 23*(1), 83–86. <https://doi.org/10.1016/0005-7967(85)90146-9>

Emmelkamp, P. M., Mersch, P.-P., Vissia, E., & Van der Helm, M. (1985). Social phobia: A comparative evaluation of cognitive and behavioral interventions. *Behaviour Research and Therapy, 23*(3), 365–369. <https://doi.org/10.1016/0005-7967(85)90015-4>

Emmelkamp, P. M., Visser, S., & Hoekstra, R. J. (1988). Cognitive therapy vs exposure in vivo in the treatment of obsessive-compulsives. *Cognitive Therapy and Research, 12*(1), 103–114.  <https://doi.org/10.1007/BF01172784>

Eseadi, C., Anyanwu, J. I., Ogbuabor, S. E., & Ikechukwu-Ilomuanya, A. B. (2016). Effects of cognitive restructuring intervention program of rational-emotive behavior therapy on adverse childhood stress in Nigeria. *Journal of Rational-Emotive and Cognitive-Behavior Therapy, 34*(1), 51–72. <https://doi.org/10.1007/s10942-015-0229-4>

Flanagan, R., Povall, L., Dellino, M., & Byrne, L. (1998). A comparison of problem solving with and without rational emotive behavior therapy to improve children's social skills. *Journal of Rational-Emotive and Cognitive-Behavior Therapy, 16*(2), 125–134. <https://doi.org/10.1023/A:1024986327879>

Forman, S. G., & Forman, B. D. (1980). Rational‐emotive staff development. *Psychology in the Schools, 17*(1), 90-96. [https://doi.org/10.1002/1520-6807(198001)17:1<90::AID-PITS2310170116>3.0.CO;2-2](https://doi.org/10.1002/1520-6807(198001)17:1%3c90::AID-PITS2310170116%3e3.0.CO;2-2)

Gavita, O. A., & Calin, A. (2013). Retman rational stories versus rational parenting program for the treatment of child psychopathology: Efficacy of two formats of rational-emotive behavior therapy. *Journal of Evidence-Based Psychotherapies, 13*(1), 33-56.

Grove, A. B., Kurtz, E. D., Wallace, R. E., Sheerin, C. M., & Scott, S. M. (2021). Effectiveness of a rational emotive behavior therapy (REBT)-informed group for post-9/11 Veterans with posttraumatic stress disorder (PTSD). *Military Psychology, 33*(4), 217-227. <https://doi.org/10.1080/08995605.2021.1897496>

Grove, A. B., Sheerin, C. M., Wallace, R. E., Green, B. A., Minnich, A. H., & Kurtz, E. D. (2023). The effect of a reduction in irrational beliefs on Posttraumatic Stress Disorder (PTSD), depression, and anxiety symptoms in a group treatment for post-9/11 Veterans. *Military Psychology*, 1–11. <https://doi.org/10.1080/08995605.2023.2236924>

Hamberger, K., & Lohr, J. M. (1980). Rational restructuring for anger control: A quasi-experimental case study. *Cognitive Therapy and Research*, *4*(1), 99–102. <https://doi.org/10.1007/BF01173359>

Horan, J. J. (1996). Effects of computer-based cognitive restructuring on rationally mediated self-esteem. *Journal of Counseling Psychology, 43*(4), 371–375. <https://doi.org/10.1037/0022-0167.43.4.371>

Hovland, O. J. (1995). Self-defeating anxiety explored: The contribution of terror management theory and rational-emotive therapy. *Anxiety, Stress and Coping: An International Journal, 8*(2), 161–182. <https://doi.org/10.1080/10615809508249371>

Huber, C. H., & Milstein, B. (1985). Cognitive restructuring and a collaborative set in couples' work. *American* *Journal of Family Therapy, 13*(2), 17–27. <https://doi.org/10.1080/01926188508250218>

Hymen, S. P., & Warren, R. (1978). An evaluation of rational-emotive imagery as a component of rational-emotive therapy in the treatment of test anxiety. *Perceptual and Motor Skills, 46*(3), 847–853. <https://doi.org/10.2466/pms.1978.46.3.847>

Ifeanyieze, F. O., Ede, M. O., Ejiofor, T. E., Ekenta, L. U., Onah, O., Okechukwu, F. C., Isiwu, E. C., Ogbonna, E., Azunku, F., Nwankwo, C. U., Ezebuiro, F., Onah, F. C., Mezieobi, D., Ede, K., Onyeanusi, O. C., Asogwa, V. O., Omeje, B., Abubakar, I., & Samuel, M. (2021). Irrational career beliefs in agricultural education students, demographics, impacts, and rational career education intervention. *Medicine, 100*(22), e26168.

Ifelunni, C. O., Ede, M. O., & Okeke, C. I. (2022). Rational emotive intervention for work-family conflict and female primary school teachers’ well-being. *Current Psychology*, 1-14. <https://doi.org/10.1007/s12144-022-03704-9>

Igna, R., Ştefan, S., Onac, I., Onac, I., Ungur, R. A., & Szentagotai Tatar, A. (2014). Mindfulness-based cognitive-behavior therapy (MCBT versus virtual reality (VR) enhanced CBT, versus treatment as usual for chronic back pain. A clinical trial. *Journal of Evidence-Based* *Psychotherapies, 14*(2), 229–247.

Iremeka, F. U., Okeke, S. A. C., Agu, P. U., Isilebo, N. C., Aneke, M., Ezepue, E. I., Ezenwaji, I. O., Ezenwaji, C. O., Edikpa, E., Chukwu, C. J., Eze, A. F., Omeje, H. O., Okereke, G. K. O., Ogidi, C. I., & Chukwuji, C. (2021). Intervention for stress management among skilled construction workers. *Medicine, 100*(28), e26621. <https://doi.org/10.1097/MD.0000000000026621>

Jacobs, E., & Croake, J. W. (1976). Rational emotive theory applied to groups. *Journal of College Student Personnel, 17*(2), 127–129.

Jacobsen, R. H., Tamkin, A. S., & Blount, J. B. (1987). The efficacy of rational-emotive group therapy in psychiatric inpatients. *Journal of Rational-Emotive Therapy, 5*(1), 22–31. <https://doi.org/10.1007/BF01080517>

Jalali, M. D., Moussavi, M. S., Amin Yazdi, S. A., & Salehi Fadardi, J. (2014). Effectiveness of rational emotive behavior therapy on psychological well-being of people with late blindness. *Journal of Rational-Emotive and Cognitive-Behavior Therapy, 32*, 233-247. <https://10.1007/s10942-014-0191-6>

Johnson, W. B., & Ridley, C. R. (1992). Brief Christian and non-Christian rational-emotive therapy with depressed Christian clients: An exploratory study. *Counseling and Values, 36*(3), 220–229. <https://doi.org/10.1002/j.2161-007X.1992.tb00790.x>

Jones, J. K., Turner, M. J., & Barker, J. B. (2021). The effects of a cognitive–behavioral stress intervention on the motivation and psychological well-being of senior U.K. police personnel. *International Journal of Stress Management, 28*(1), 46–60. <https://doi.org/10.1037/str0000218>

Jordana, A., Torregrossa, M., & Ramis, Y. (2022). Una intervención TREC para la recuperación de actividad física saludable en exdeportistas de élite [A REBT intervention for the recovery of healthy physical activity in former elite athletes] *Cuadernos de Psicología del Deporte/Sport Psychology Notebooks, 22*(3), 25-40.

Joyce, M. R. (1995). Emotional relief for parents: Is rational-emotive parent education effective? *Journal of Rational-Emotive and Cognitive-Behavior Therapy, 13*(1), 55–75. <https://doi.org/10.1007/BF02354557>

Kabasakal, E., & Emiroğlu, O. N. (2021). The effect of rational‐emotive education on irrational thinking, subjective wellbeing and self‐efficacy of typically developing students and social acceptance of disabled students. *Child: Care, Health and Development, 47*(4), 411–421. <https://doi.org/10.1111/cch.12819>

Kanter, N. J., & Goldfried, M. R. (1979). Relative effectiveness of rational restructuring and self-control desensitization in the reduction of interpersonal anxiety. *Behavior Therapy, 10*(4), 472–490. <https://doi.org/10.1016/S0005-7894(79)80051-9>

Kara, E., Türküm, A. S., & Turner, M. J. (2023). The effects of rational emotive behaviour therapy (REBT) group counselling program on competitive anxiety of student-athletes. *Journal of Rational-Emotive and Cognitive-Behavior Therapy*, *41,* 362 – 379. <https://doi.org/10.1007/s10942-023-00497-z>

Kassinove, H., Miller, N., & Kalin, M. (1980). Effects of pretreatment with rational emotive bibliotherapy and rational emotive audiotherapy on clients waiting at community mental health center. *Psychological Reports, 46*(3), 851–857. <https://doi.org/10.2466/pr0.1980.46.3.851>

Keller, J. F., Croake, J. W., & Brooking, J. Y. (1975). Effects of a program in rational thinking on anxieties in older persons. *Journal of Counseling Psychology, 22*(1), 54–57. <https://doi.org/10.1037/h0076144>

Kirkby, R. J. (1994). Changes in premenstrual symptoms and irrational thinking following cognitive-behavioral coping skills training. *Journal of Consulting and Clinical Psychology, 62*(5), 1026–1032. <https://doi.org/10.1037/0022-006X.62.5.1026>

Knapp, S., Miller, A., Outar, L., & Turner, M. (2023). Psychological well-being and exercise addiction: The treatment effects of an REBT intervention for females. *Psychology of Sport and Exercise, 64*, 102298. <https://doi.org/10.1016/j.psychsport.2022.102298>

Komasi, S., Saeidi, M., Zakiei, A., Amiri, M. M., & Soltani, B. (2017). Cognitive restructuring based on metaphor therapy to challenge the irrational beliefs of drug addicts undergoing buprenorphine treatment. *International Journal of High Risk Behaviors and Addiction, 6*(1), e31450. <https://doi.org/10.5812/ijhrba.31450>

Kushnir, T., & Malkinson, R. (1993). A rational-emotive group intervention for preventing and coping with stress among safety officers. *Journal of Rational-Emotive and Cognitive-Behavior Therapy, 11*(4), 195–206. <https://doi.org/10.1007/BF01089775>

Kushnir, T., Malkinson, R., & Ribak, J. (1994). Teaching stress management skills to occupational and environmental health physicians and practitioners: A graduate-level practicum. *Journal of Occupational Medicine, 36*(12), 1335-1340.

Kushnir, T., Malkinson, R., & Ribak, J. (1998). Rational thinking and stress management in health workers: A psychoeducational program. *International Journal of Stress Management, 5*, 169-178. <https://doi.org/10.1023/A:1022941031900>

Lake, A., Rainey, J., & Papsdorf, J. D. (1979). Biofeedback and rational-emotive therapy in the management of migraine headache. *Journal of Applied Behavior Analysis, 12*(1), 127–140. <https://doi.org/10.1901/jaba.1979.12-127>

Leaf, R. C., Gross, P. H., Todres, A. K., Marcus, S., & Bradford, B. (1986). Placebo-like effects of education about rational-emotive therapy. *Psychological Reports, 58*(2), 351–370. <https://doi.org/10.2466/pr0.1986.58.2.351>

Lipsky, M. J., Kassinove, H., & Miller, N. J. (1980). Effects of rational-emotive therapy, rational role reversal, and rational-emotive imagery on the emotional adjustment of community mental health center patients. *Journal of Consulting and Clinical Psychology, 48*(3), 366–374. <https://doi.org/10.1037/0022-006X.48.3.366>

Lupu, V., & Iftene, F. (2009). The impact of rational emotive behaviour education on anxiety in teenagers. *Journal of Cognitive and Behavioral Psychotherapies, 9*(1), 95-105.

Mahfar, M., Aslan, A. S., Noah, S. M., Ahmad, J., & Jaafar, W. M. W. (2014). Effects of rational emotive education module on irrational beliefs and stress among fully residential school students in Malaysia. *Procedia-Social and Behavioral Sciences, 114*, 239-243. <https://doi.org/10.1016/j.sbspro.2013.12.692>

Mattick, R. P., & Peters, L. (1988). Treatment of severe social phobia: Effects of guided exposure with and without cognitive restructuring. *Journal of Consulting and Clinical Psychology, 56*(2), 251–260. <https://doi.org/10.1037/0022-006X.56.2.251>

Mattick, R. P., Peters, L., & Clarke, J. C. (1989). Exposure and cognitive restructuring for social phobia: A controlled study. *Behavior Therapy, 20*(1), 3–23. <https://doi.org/10.1016/S0005-7894(89)80115-7>

Maxwell-Keys, C., Wood, A. G., & Turner, M. J. (2022). Developing decision making in Rugby Union match officials using rational emotive behavior therapy (REBT). *Psychology of Sport and Exercise, 58*, 102098. <https://doi.org/10.1016/j.psychsport.2021.102098>

McCormick, N., Tooke, W., Winston, S., & Kjellander, C. (1991). RET in the college classroom. *Journal of Rational-Emotive & Cognitive-Behavior Therapy, 9*(2), 95–111. <https://doi.org/10.1007/BF01062113>

McKnight, D. L., Nelson, R. O., Hayes, S. C., & Jarrett, R. B. (1984). Importance of treating individually assessed response classes in the amelioration of depression. *Behavior Therapy, 15*(4), 315–335. <https://doi.org/10.1016/S0005-7894(84)80001-5>

McNaughton-Cassill, M. E., Bostwick, J. M., Arthur, N. J., Robinson, R. D., & Neal, G. S. (2002). Efficacy of brief couples support groups developed to manage the stress of in vitro fertilization treatment. *Mayo Clinic Proceedings, 77*(10), 1060-1066. <https://doi.org/10.4065/77.10.1060>

Mersch, P. P. A. (1995). The treatment of social phobia: The differential effectiveness of exposure in vivo and an integration of exposure in vivo, rational emotive therapy and social skills training. *Behaviour Research and therapy, 33*(3), 259-269. <https://doi.org/10.1016/0005-7967(94)00038-L>

Mersch, P. P. A., Emmelkamp, P. M., Bögels, S. M., & Van der Sleen, J. (1989). Social phobia: Individual response patterns and the effects of behavioral and cognitive interventions. *Behaviour Research and Therapy, 27*(4), 421–434. <https://doi.org/10.1016/0005-7967(89)90013-2>

Mersch, P. P. A., Emmelkamp, P. M., & Lips, C. (1991). Social phobia: Individual response patterns and the long-term effects of behavioral and cognitive interventions. A follow-up study. *Behaviour Research and Therapy, 29*(4), 357–362. <https://doi.org/10.1016/0005-7967(91)90072-B>

Miller, N., & Kassinove, H. (1978). Effects of lecture, rehearsal, written homework, and IQ on the efficacy of a rational emotive school mental health program. *Journal of Community Psychology, 6*(4), 366–373.

Mio, M. G., & Matsumuto, Y. (2018). A single-session universal mental health promotion program in Japanese schools: A pilot study. S*ocial Behavior and Personality: An International Journal, 46*(10), 1727-1743. <https://doi.org/10.2224/sbp.7157>

Möller, A. T., & Botha, H. C. (1996). Effects of a group rational-emotive behavior therapy program on the Type A behavior pattern. *Psychological Reports, 78*, 947–961. <https://doi.org/10.2466/pr0.1996.78.3.947>

Möller, A. T., Kotzé, H. F., & Sieberhagen, K. J. (1993). Comparison of the effects of auditory subliminal stimulation and rational-emotive therapy, separately and combined, on self-concept. *Psychological Reports, 72*(1), 131-145.

Moon, J. R., Huh, J., Song, J., Kang, I. S., Park, S. W., Chang, S. A., Chang, S., Yang, J., Jun, T., & Han, J. S. (2021). The effects of rational emotive behavior therapy for depressive symptoms in adults with congenital heart disease. *Heart and Lung, 50*(6), 906-913. <https://doi.org/10.1016/j.hrtlng.2021.07.011>

Morris, G. B. (1992). R.A.D.A.R.: A five-session approach for referrals of employee assistance programs. *Journal of Cognitive Psychotherapy, 6*(4), 259–276.

Morris, G. B. (1993). A rational-emotive treatment program with conduct disorder and attention-deficit hyperactivity disorder adolescents. *Journal of Rational-Emotive and Cognitive-Behavior Therapy, 11*(3), 123-134.

Mueller, R., & Moskowitz, L. J. (2020). Positive family intervention for children with ASD: Impact on parents’ cognitions and stress. *Journal of Child and Family Studies*, *29*(12), 3536-3551. <https://doi.org/10.1007/s10826-020-01830-1>

Munjack, D. J., Schlaks, A., Sanchez, V. C., Usigli, R., Zulueta, A., & Leonard, M. (1984). Rational-emotive therapy in the treatment of erectile failure: An initial study. *Journal of Sex and Marital Therapy, 10*(3), 170-175. <https://doi.org/10.1080/00926238408405942>

Neamtu, G. M., & David, O. A. (2016). Coaching emotional abilities in fostered adolescents through rational emotive and cognitive-behavioral education: Efficacy and mechanisms of change of using therapeutic stories. *Journal of Evidence-Based Psychotherapies, 16*(1), 33-56.

Nejati, M., Farsi, A., Moteshareie, E., Miller, A., & Turner, M. J. (2022). The effects of rational emotive behaviour therapy on performance under pressure in adolescent soccer athletes: A randomised control design. *International* *Journal of Sport and Exercise Psychology*, 1-18. <https://doi.org/10.1080/1612197X.2022.2152852>

Newhouse, R. C., & Schwager, H. (1978). Rational behavior therapy as related to self-concept of disadvantaged adults. *Journal of Instructional Psychology, 5*(1), 35-38.

Nielsen, D. M., Horan, J. J., Keen, B., St Peter, C. C., Ceperich, S. D., & Ostlund, D. (1996). An Attempt to Improve Self-Esteem by Modifying Specific Irrational Beliefs. *Journal of Cognitive Psychotherapy, 10*(2), 137-149.

Nottingham IV, E. J., & Neimeyer, R. A. (1992). Evaluation of a comprehensive inpatient rational-emotive therapy program: Some preliminary data. *Journal of Rational-Emotive and Cognitive-Behavior Therapy, 10*(2), 57-81.

Ofoegbu, T. O., Asogwa, U. D., Eseadi, C., Ogbonna, C. S., Eskay, M., Obiyo, N. O., Nji, G. C., Ngwoke, O. R., Agboti, C. I., Uwakwe, R. C., & Ogbuabor, S. E. (2021). Effect of rational digital storytelling intervention on depression among adolescent-athletes with special educational needs. *Journal of Rational-Emotive & Cognitive-Behavior Therapy, 39*(2), 217–237. <https://doi.org/10.1007/s10942-020-00366-z>

Ogbuanya, T. C., Eseadi, C., Orji, C. T., Anyanwu, J. I., Joachim, O. C., & Otu, M. S. (2018). The effect of rational emotive behavior therapy on irrational career beliefs of students of electrical electronics and other engineering trades in technical colleges in Nigeria. *Journal of Rational-Emotive and Cognitive-Behavior Therapy, 36*, 201-219. <https://doi.org/10.1007/s10942-017-0282-2>

Ogbuanya, T. C., Eseadi, C., Orji, C. T., Ohanu, I. B., Bakare, J., & Ede, M. O. (2017). Effects of rational emotive behavior coaching on occupational stress and work ability among electronics workshop instructors in Nigeria. *Medicine, 96*(19), e6891. <https://doi.org/10.1097/MD.0000000000006891>

Omeje, J. C., Otu, M. S., Aneke, A. O., Adikwu, V. O., Nwaubani, O. O., Chigbu, E. F., Onuigbo, L. N., Udom, I. E., Aye, N. E., Akaneme, I. N., Egeonu, D. C., Ezema, V. C., Okpanachi, G. O., Ohabuenyi, A. G., Eseadi, E., & Eze, N. M. (2018). Effect of rational emotive health therapy on alcohol use among community-dwelling, HIV-positive patients. *Medicine, 97* (35), e11688. <https://doi.org/10.1097/MD.0000000000011688>

Onuigbo, L. N., Eseadi, C., Ugwoke, S. C., Nwobi, A. U., Anyanwu, J. I., Okeke, F. C., Agu, P. U., Oboegbulem, A. I., Chinweuba, N. H., Agundu, U., Ololo, K.O., Okpoko, C., Nwankwor, P. P., Eze, U, N., & Eze, P. (2018). Effect of rational emotive behavior therapy on stress management and irrational beliefs of special education teachers in Nigerian elementary schools, *Medicine, 97* (37), e12191. <https://doi.org/10.1097/MD.0000000000012191>

Onyemaechi Ede, M., Okeke, C. I., & Oneli, J. O. (2023). Raising the self-esteem and reducing irrational beliefs of schoolchildren: The moderating and main effect study. *Medicine*, *102*(27), e34168. <https://doi.org/10.1097/MD.0000000000034168>

Otu, M. S., & Omeje, J. C. (2021). The effect of rational emotive career coaching on dysfunctional career beliefs in recent university graduates. *Journal of Rational-Emotive and Cognitive-Behavior Therapy, 39*, 555–577. <https://doi.org/10.1007/s10942-020-00383-y>

Outar, L., Turner, M. J., Wood, A. G., & Lowry, R. (2018). “I need to go to the gym”: Exploring the use of rational emotive behaviour therapy upon exercise addiction, irrational and rational beliefs. *Performance Enhancement and Health, 6*(2), 82-93. <https://doi.org/10.1016/j.peh.2018.05.001>

Outar, L., Turner, M. J., Wood, A. G., & O'Connor, H. (2021). Muscularity rationality: An examination of the use of rational emotive behaviour therapy (REBT) upon exercisers at risk of muscle dysmorphia. *Psychology of Sport and Exercise, 52*, 101813. <https://doi.org/10.1016/j.psychsport.2020.101813>

Păsărelu, C. R., Dobrean, A., Andersson, G., & Zaharie, G. C. (2021). Feasibility and clinical utility of a transdiagnostic internet-delivered rational emotive and behavioral intervention for adolescents with anxiety and depressive disorders. *Internet Interventions, 26*, 100479. <https://doi.org/10.1016/j.invent.2021.100479>

Popa, C., & Predatu, R. (2019). The effect of an integrative CBT/REBT intervention in improving emotional functioning and emotional stability in Romanian medical students. *Journal of Evidence-Based Psychotherapies, 19*(1), 59–71. <https://doi.org/10.24193/jebp.2019.1.4>

Popa, C. O., Sava, F. A., Muresan, S., Schenk, A., Cojocaru, C. M., Muntean, L. M., & Olah, P. (2022). Standard CBT versus integrative and multimodal CBT assisted by virtual-reality for generalized anxiety disorder. *Frontiers in Psychology, 13*, 1008981. <https://doi.org/10.3389/fpsyg.2022.1008981>

Ray, J. B., Freidlander, R. B., & Solomon, G. S. (1984). Changes in rational beliefs among treated alcoholics. *Psychological Reports, 55*(3), 883–886. <https://doi.org/10.2466/pr0.1984.55.3.883>

Rezaeisharif, A., Karimi, A., & Naeim, M. (2021). Effectiveness of the cognitive restructuring approach on irrational beliefs and hopelessness in individuals with a substance abuse disorder: A randomized controlled trial. *Addictive Disorders and Their Treatment*, *20*(4), 326-335. <https://doi.org/10.1097/ADT.0000000000000264>

Riggs, R. C., & Meyer, R. L. (1981). Training paraprofessional group counseling leaders in the federal prison system. *Journal for Specialists in Group Work, 6*(2), 96-99. <https://doi.org/10.1080/01933928108411372>

Roman, O. T. (2011). Rational emotive behaviour therapy features on intrinsically religiously oriented people. *Journal of Psychological and Educational Research, 19*(2), 67-95.

Rosenbaum, T., McMurray, N. E., & Campbell, I. M. (1991). The effects of rational emotive education on locus of control, rationality and anxiety in primary school children. *Australian Journal of Education, 35*(2), 187-200.

Şahin, H., & Türk, F. (2021). The impact of cognitive-behavioral group psycho-education program on psychological resilience, irrational beliefs, and well-being. *Journal of Rational-Emotive and Cognitive-Behavior Therapy, 39*(4), 672-694. <https://doi.org/10.1007/s10942-021-00392-5>

Schenk, A., Popa, C. O., Olah, P., Suciu, N., & Cojocaru, C. (2020). The efficacy of rational emotive behavior therapy intervention in generalized anxiety disorder. *Acta Marisiensis-Seria Medica, 66*(4), 148-151.

Sheehy, R., & Horan, J. J. (2004). Effects of Stress Inoculation Training for 1st-Year Law Students. *International Journal of Stress Management, 11*(1), 41–55. <https://doi.org/10.1037/1072-5245.11.1.41>

Smith, T. W. (1983). Change in irrational beliefs and the outcome of rational-emotive psychotherapy. *Journal of Consulting and Clinical Psychology, 51*(1), 156–157. <https://doi.org/10.1037/0022-006X.51.1.156>

Sousa, C. R. D., & Padovani, R. D. C. (2021). Assertive skills: A comparison of two group interventions with Brazilian university students. *Psicologia: Reflexão e Crítica, 34*, 1-9. <https://doi.org/10.1186/s41155-021-00188-7>

Stanton H. E. (1989). Hypnosis and rational-emotive therapy-a de-stressing combination: A brief communication. *The International Journal of Clinical and Experimental Hypnosis, 37*(2), 95–99. <https://doi.org/10.1080/00207148908410538>

Syzmanski, J., & O'Donohue, W. (1995). The potential role of state-dependent learning in cognitive therapy with spider phobics. *Journal of Rational-Emotive and Cognitive-Behavior Therapy, 13*(2), 131–150. <https://doi.org/10.1007/BF02354458>

Szentagotai, A., David, D., Lupu, V., & Cosman, D. (2008). Rational emotive behavior therapy versus cognitive therapy versus pharmacotherapy in the treatment of major depressive disorder: Mechanisms of change analysis. Psyc*hotherapy: Theory, Research, Practice, Training, 45*(4), 523–538. <https://doi.org/10.1037/a0014332>

Thorpe, G. L., Freedman, E. G., & McGalliard, D. W. (1984). Components of rational-emotive imagery: Two experiments with nonassertive students. *Journal of Rational Emotive Therapy, 2*(2), 11-19.

Thurman, C. W. (1983). Effects of a rational–emotive treatment program on Type A behavior among college students. *Journal of College Student Personnel, 24*(5), 417–423.

Thurman, C. W. (1985a). Effectiveness of cognitive-behavioral treatments in reducing Type A behavior among university faculty. *Journal of Counseling Psychology, 32*(1), 74 – 83. <https://doi.org/10.1037/0022-0167.32.1.74>

Thurman, C. W. (1985b). Effectiveness of cognitive–behavioral treatments in reducing Type A behavior among university faculty: One year later. *Journal of Counseling Psychology, 32*(3), 445 – 448. <https://doi.org/10.1037/0022-0167.32.3.445>

Tomoiagă, C., & David, O. (2022). The efficacy of guided and unguided game-based cognitive-behavioral therapy in reducing distress in college students. *Games for Health Journal, 11*(6), 403-413. <https://doi.org/10.1089/g4h.2021.0195>

Trexler, L. D., & Karst, T. O. (1972). Rational-emotive therapy, placebo, and no-treatment effects on public-speaking anxiety. *Journal of Abnormal Psychology, 79*(1), 60–67. <https://doi.org/10.1037/h0032336>

Trip, S., Dume, C., Bora, C. H., McMahon, J., & Baroll, B. (2019). The efficiency of rational-emotive behavioral education for parents. *Journal of Psychological and Educational Research, 27*(1), 61-84.

Trip, S., McMahon, J., Bora, C., & Chipea, F. (2010). The efficiency of a rational emotive and behavioral education program in diminishing dysfunctional thinking, behaviors and emotions in children. *Journal of Cognitive and Behavioral Psychotherapies, 10*(2), 173–186.

Türküm, A. S. (2007). Differential effects between group counselling and group guidance in conducting a 'Coping with Stress Training Program' for Turkish university students. *International Journal for the Advancement of Counselling, 29*(1), 69–81. <https://doi.org/10.1007/s10447-006-9029-y>

Turner, M., & Barker, J. B. (2013). Examining the efficacy of rational-emotive behavior therapy (REBT) on irrational beliefs and anxiety in elite youth cricketers. *Journal of Applied Sport Psychology, 25*(1), 131–147. <https://doi.org/10.1080/10413200.2011.574311>

Turner, M. J., & Barker, J. B. (2015). Examining the effects of rational emotive behavior therapy (REBT) on the irrational beliefs of blue-chip professionals. *Journal of Rational-Emotive and Cognitive-Behavior Therapy, 33*(1), 17–36. <https://doi.org/10.1007/s10942-014-0200-9>

Turner, M. J., Barker, J. B., & Slater, M. J. (2014). The season-long effects of rational emotive behavior therapy on the irrational beliefs of professional academy soccer athletes. *International Journal of Sport Psychology, 45*(5), 429–451.

Turner, M. J., & Davis, H. S. (2019). Exploring the effects of rational emotive behaviour therapy on the irrational beliefs and self-determined motivation of triathletes. *Journal of Applied Sport Psychology, 31*, 253-272. <https://doi.org/10.1080/10413200.2018.1446472>

Turner, M. J., Ewen, D., & Barker, J. B. (2020). An idiographic single-case study examining the use of rational emotive behavior therapy (REBT) with three amateur golfers to alleviate social anxiety. *Journal of Applied Sport Psychology, 32*(2), 186–204. <https://doi.org/10.1080/10413200.2018.1496186>

Turner, M. J., Slater, M. J., & Barker, J. B. (2014). Not the end of the world: The effects of rational-emotive behavior therapy (REBT) on irrational beliefs in elite soccer academy athletes. *Journal of Applied Sport Psychology, 26*(2), 144-156. <https://doi.org/10.1080/10413200.2013.812159>

Ugwoke, E. O., Edeh, N. I., Abanyam, E. F., Azubuike, R. N., Agbo, S. U., Madu, M. A., Naboth-Odums, A., Akaeze, P., Onah, F. C., Onah, O., Isiwu, E. A., Madusaba, B. M., Nwangwu, E., Kuranen-Joko, D., & Ezebuiro, F. N. (2021). Effects of rational emotive psychotherapy consort on business education students' perception about image of technical vocational education and training in South-East Nigeria. *Medicine, 100*(32), e26758. <https://doi.org/10.1097/MD.0000000000026758>

Ugwoke, S. C., Eseadi, C., Igbokwe, C. C., Chiaha, G. T. U., Nwaubani, O. O., Orji, C. T., Ugwuanyi, L. T., Chukwuma, I. S., Edikpa, E. C., Ogakwu, V. N., Onu, E. A., Agu, P., Nwobi, U. A., Omeke, F., Okeke, F. C., Ezema, R. N., & Abugu, L. I. (2017). Effects of a rational-emotive health education intervention on stress management and irrational beliefs among technical college teachers in Southeast Nigeria. *Medicine, 96*(31), e7658. <https://doi.org/10.1097/MD.0000000000007658>

Ulusoy, Y., & Duy, B. (2013). Effectiveness of a psycho-education program on learned helplessness and irrational beliefs. *Educational Sciences: Theory and Practice, 13*(3), 1440-1446.

Urfa, O., & Aşçı, F. H. (2023). The effects of rational emotive behavior therapy and motivational interviewing on emotions, automatic thoughts, and perceived performance of elite female volleyball players. *Psychology of Sport and Exercise*, *69*, 102497. <https://doi.org/10.1016/j.psychsport.2023.102497>

Vaida, S., Kállay, E., & Opre, A. (2008). Counseling in schools: A rational emotive behavior therapy (REBT) Based Intervention. *Romanian Association for Cognitive Science, 10*(1), 57–69.

Vertopoulos, E., & Turner, M. J. (2017). Examining the effectiveness of a rational emotive personal-disclosure mutual-sharing (REPDMS) intervention on the irrational beliefs and rational beliefs of Greek adolescent athletes. *The Sport Psychologist, 31*(3), 264–274. <https://doi.org/10.1123/tsp.2016-0071>

Victor-Aigbodion, V., Eseadi, C., Ardi, Z., Sewagegn, A. A., Ololo, K., Abonor, L. B., Aloh, H. E., Falade, T. A., & Effanga, O. A. (2023). Effectiveness of rational emotive behavior therapy in reducing depression among undergraduate medical students. *Medicine*, *102*(4), e32724. <https://doi.org/10.1097/MD.0000000000032724>

Warren, R., McLellarn, R. W., & Ponzoha, C. (1988). Rational-emotive therapy vs general cognitive-behavior therapy in the treatment of low self-esteem and related emotional disturbances. *Cognitive Therapy and Research, 12*(1), 21–37. <https://doi.org/10.1007/BF01172778>

Warren, R., Smith, G., & Velten, E. (1984). Rational-emotive therapy and the reduction of interpersonal anxiety in junior high school students. *Adolescence, 19*(76), 893–902.

Wessel, I., & Mersch, P. P. A. (1994). A cognitive-behavioural group treatment for test-anxious adolescents. *Anxiety, Stress and Coping: An International Journal, 7*(2), 149–160. <https://doi.org/10.1080/10615809408249341>

Wilde, J. (1996a). The efficacy of short-term rational-emotive education with fourth-grade students. *Elementary School Guidance and Counseling, 31*(2), 131-138.

Wilde, J. (1999). The efficacy of short-term rational-emotive education: A follow-up evaluation. *Journal of Cognitive Psychotherapy*, *13*(2), 133-143. <https://doi.org/10.1891/0889-8391.13.2.133>

Wilde, J. K. (1996b). The relationship between rational thinking and intelligence in children. *Journal of Rational-Emotive and Cognitive-Behavior Therapy, 14*(3), 187-192.

Wood, A. G., Barker, J. B., & Turner, M. J. (2017). Developing performance using rational emotive behavior therapy (REBT): A case study with an elite archer. *The Sport Psychologist, 31*(1), 78-87. <https://doi.org/10.1123/tsp.2015-0083>

Wood, A. G., Barker, J. B., Turner, M. J., & Sheffield, D. (2018). Examining the effects of rational emotive behavior therapy on performance outcomes in elite paralympic athletes. *Scandinavian Journal of Medicine and Science in Sports, 28*(1), 329–339. <https://doi.org/10.1111/sms.12926>

Wood, A. G., Barker, J. B., Turner, M., & Thomson, P. (2018). Exploring the effects of a single rational emotive behavior therapy workshop in elite blind soccer players. *The Sport Psychologist, 32*(4), 321-332. <https://doi.org/10.1123/tsp.2017-0122>

Wood, A., Mack, R., & Turner, M. (2020). Developing self-determined motivation and performance with an elite athlete: Integrating motivational interviewing with rational emotive behavior therapy. *Journal of Rational-Emotive and Cognitive-Behavior Therapy, 38*(4), 540–567. <https://doi.org/10.1007/s10942-020-00351-6>

Wood, A. G., Wilkinson, A., Turner, M. J., Haslam, C. O., & Barker, J. B. (2021). Into the fire: Applying rational emotive behavioral coaching (REBC) to reduce irrational beliefs and stress in fire service personnel. *International Journal of Stress Management, 28*(3), 232–243. <https://doi.org/10.1037/str0000228>

Woods, P. J. (1987). Reductions in Type A behavior, anxiety, anger, and physical illness as related to changes in irrational beliefs: Results of a demonstration project in industry. *Journal of Rational Emotive Therapy, 5*(4), 213-237.

Xu, L., & Liu, H. (2017). Effects of rational emotive behavior therapy (REBT) intervention program on mental health in female college students. *Neuroquantology, 15*(4), 156-162.

Yu, A., & Schill, T. (1976). Rational-emotive therapy as a treatment in reducing vulnerability to criticism. Rational Living, 11(2), 12–14.
